# Iodine-mediated oxythiolation of *o*-vinylanilides with disulfides for the synthesis of benzoxazines†

**DOI:** 10.1039/d2ra01078j

**Published:** 2022-03-04

**Authors:** Yu Yang, Liyan Liu, Kuiliang Li, Zhenggen Zha, Qi Sun, Zhiyong Wang

**Affiliations:** School of Chemistry and Chemical Engineering, Hefei Normal University Hefei 230601 China; Hefei National Laboratory for Physical Sciences at Microscale, CAS Key Laboratory of Soft Matter Chemistry, Department of Chemistry, University of Science and Technology of China Hefei Anhui 230026 P. R. China

## Abstract

An efficient iodine-mediated oxythiolation of *o*-vinylanilides with disulfides was developed. By virtue of this method, a series of thio-tethered benzoxazine derivatives were synthesized in good to excellent yields. The reaction features high yields, is metal-free, and has a wide substrate scope.

## Abstract 2

An efficient iodine-mediated oxythiolation of *o*-vinylanilides with disulfides was developed. The reaction features high yields, is metal-free, and has a wide substrate scope.

## Graphical abstract

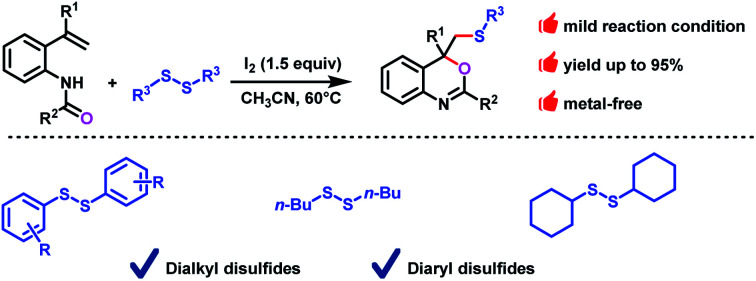

As one of the most basic organic skeletons, alkenes are important starting materials in organic synthesis. It is convenient to introduce functional groups into the carbon chain by addition reaction.^1,2^ Furthermore, heterocyclic compounds also could be built efficiently from alkenes by addition–cyclization with reasonable design of starting materials.^3–5^ For example, benzo[*d*][1,3]oxazine, an essential heterocyclic skeleton which is widely found in natural products and biological active molecule,^6–10^ could be synthesized by the intramolecular cyclization of *N*-(2-(1-phenylvinyl)phenyl)-benzamides (Scheme 1).^11–23^ In the 1980s, Capozzi and co-workers synthesized methylthio-benzo[*d*][1,3]oxazine with this strategy for the first time, using Me_3_S_3_SbCl_6_ as the methylthiolating agent (Scheme 1a).^19^ Besides, this was the only way to generate this kind of compound for the next three decades. In 2018, Anbarasan and co-workers found that *N*-(arylthio)-succinimides could also be used as an electrophilic reagent to react with *N*-(2-vinylphenyl)amides, generating a series of arylthio benzo[*d*][1,3]-oxazine derivatives (Scheme 1b).^20^ Recently, different sulfonyl sources, such as sulfur dioxides,^21^ sodium sulfonates^22^ and sulfonylhydrazines,^23^ were developed in this methodology to afford sulfonated benzo-[*d*][1,3]oxazine. The studies mentioned above provided a series of tools to build sulfur-containing benzo[*d*][1,3]oxazines. However, there are still some limitations in these procedures: (1) the involvement of expensive metals or a large amount of acid; (2) limited substrate applicability. In view of the importance of sulfur-containing compounds,^24–31^ it is still highly desirable to develop a simple and efficient method to build thio-tethered compounds with good reaction compatibility under mild conditions.

**Scheme 1:**
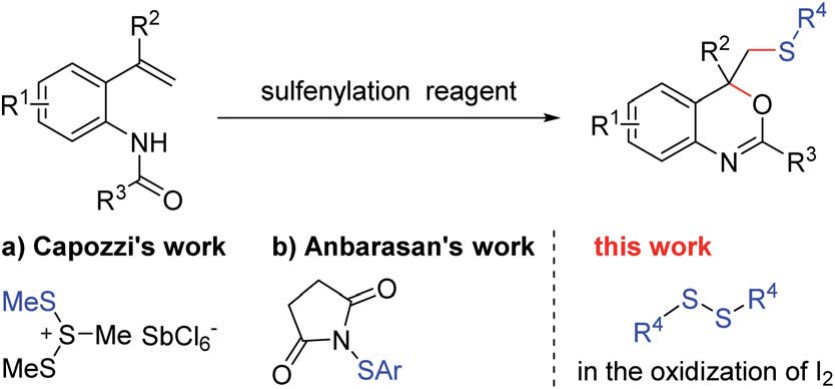
Building sulfur-containing benzo[*d*][1,3]oxazines from *o*-vinylanilides.

Our group have been focusing on green synthetic methods for a long time.^3,4^ It was reported that disulfide, a sulfenylation reagent, could generate two sulfur synthons by the cleavage of S–S bond without any by-product.^32–39^ Based on these previous studies, we are going to develop a more efficient oxythiolation reaction to build sulfur-containing heterocyclic compounds. Herein we disclose an iodine mediated cyclization of *o*-vinylanilides with disulfides to form thio-benzo[*d*][1,3]oxazines, in which both aryl and alkyl disulfides could performed well to afford corresponding products with good yield under mild condition.

At the outset of this study, *N*-(2-(1-phenylvinyl)phenyl)-benzamide (1a) and 1,2-diphenyldisulfide (2a) were employed as the substrates to optimize the reaction conditions (Table 1). Initially, 1a (1 equiv.) and 2a (0.5 equiv.) were treated with I_2_ (1.0 equiv.) in DCE at 50 °C for 12 hours. To our delight, the cyclization product 3aa was obtained in 57% yield under the promotion of I_2_ (entry 1). When other oxidants, such as [bis(trifluoroacetoxy)iodo]benzene (PIFA) or (diacetoxyiodo)benzene (PIDA), were used instead of I_2_, no corresponding product was observed (entries 2 and 3). Next, the efficiency of different solvents was tested. Screening of solvent showed CH_3_CN to be the optimal solvent, affording 3aa in 63% yield (entries 4**–**9). It was found that the yield of product 3aa could be increased to 81% by increasing the dosage of 2a and I_2_ from 0.5 equiv. to 0.75 equiv. and 1.0 equiv. to 1.5 equiv., respectively (entry 10). However, continuously increasing the amount of 2a and I_2_ had no effect on the yield (entry 11). On the other hand, the reaction temperature had a great influence on the reaction. For instance, the yield of 3aa was enhanced to 87% by elevating the reaction temperature to 60 °C. Further increase in temperature led to a decrease in yield, perhaps due to the oxidation of 2a and the volatilization of molecular iodine at 70 or 80 °C (entries 13 and 14).

**Table 1:** Optimization of the reaction conditions^a^

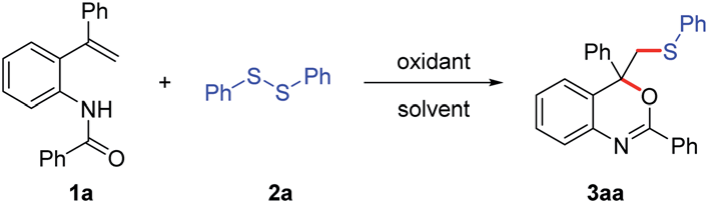				
Entry	Oxidant	Solvent	Temp. (°C)	Yield^b^ (%)
1	I_2_	DCE	50	57
2	PIDA	DCE	50	n.d.^c^
3	PIFA	DCE	50	n.d.
4	I_2_	DMSO	50	n.d.
5	I_2_	DMF	50	n.d.
6	I_2_	CH_3_CN	50	63
7	I_2_	1,4-Dioxane	50	35
8	I_2_	EtOH	50	<5
9	I_2_	Toluene	50	<5
10^d^	I_2_	CH_3_CN	50	81
11^e^	I_2_	CH_3_CN	50	81
12^d^	I_2_	CH_3_CN	60	87
13^d^	I_2_	CH_3_CN	70	72
14^d^	I_2_	CH_3_CN	80	60

a Reaction condition: 1a (0.2 mmol, 1 equiv.), 2a (0.1 mmol, 0.5 equiv.), oxidant (0.2 mmol, 1 equiv.), 12 h.

b Isolated yield.

c n.d. = not detected.

d 2a (0.75 equiv.) and I_2_ (1.5 equiv.) were used.

e 2a (1.0 equiv.) and I_2_ (2.0 equiv.) were used.

With a set of optimized conditions in hand, we studied the scope of this reaction. A series of *N*-(2-vinylphenyl) amides 1a–1q were subjected to the reaction with disulfide 2a (Scheme 2). Most of the *N*-(2-vinylphenyl) amides were found to be applicable, affording the corresponding products in good to excellent yields. The effect of the substituent R^1^ in the aryl moiety of substrates 1 was studied. When the R^1^ was halide, the reaction could be carried out smoothly (3ba–3da). When R^1^ was NO_2_, the corresponding product 3ea could also be isolated in 70% yield. Substrates 1 bearing electro-donating groups, such as OMe, Me and *n*-Bu, were suitable for this transformation, affording the cyclization product 3fa–3ha with excellent yields (90–95%). *N*-(4-(*tert*-Butyl)-2-(1-phenylvinyl)phenyl)-benzamide (1i) bearing a bulky *tert*-butyl group, moreover, also worked well in this reaction in spite of a slightly lower yield (3ia). In general, the substrates with electron-donating groups (R = Me, OMe, *n*-Bu, *t*-Bu) on the phenyl ring gave slightly higher yields than that bearing electron-withdrawing groups (R = F, Cl, Br, NO_2_). For different aromatic substituents R^2^ on the vinylic double bond, both halide and methyl were tolerated under the reaction conditions, affording the corresponding product 3ka–3na in good yields (68–83%). Substrate 1 with different protecting groups were also proceeded smoothly in this reaction to give the corresponding product 3oa and 3pa with the yields of 95% and 67%, respectively. It was noted that when R^3^ was methyl instead of aryl, the cyclization product 3pa was also obtained in 67% yield.

**Scheme 2:**
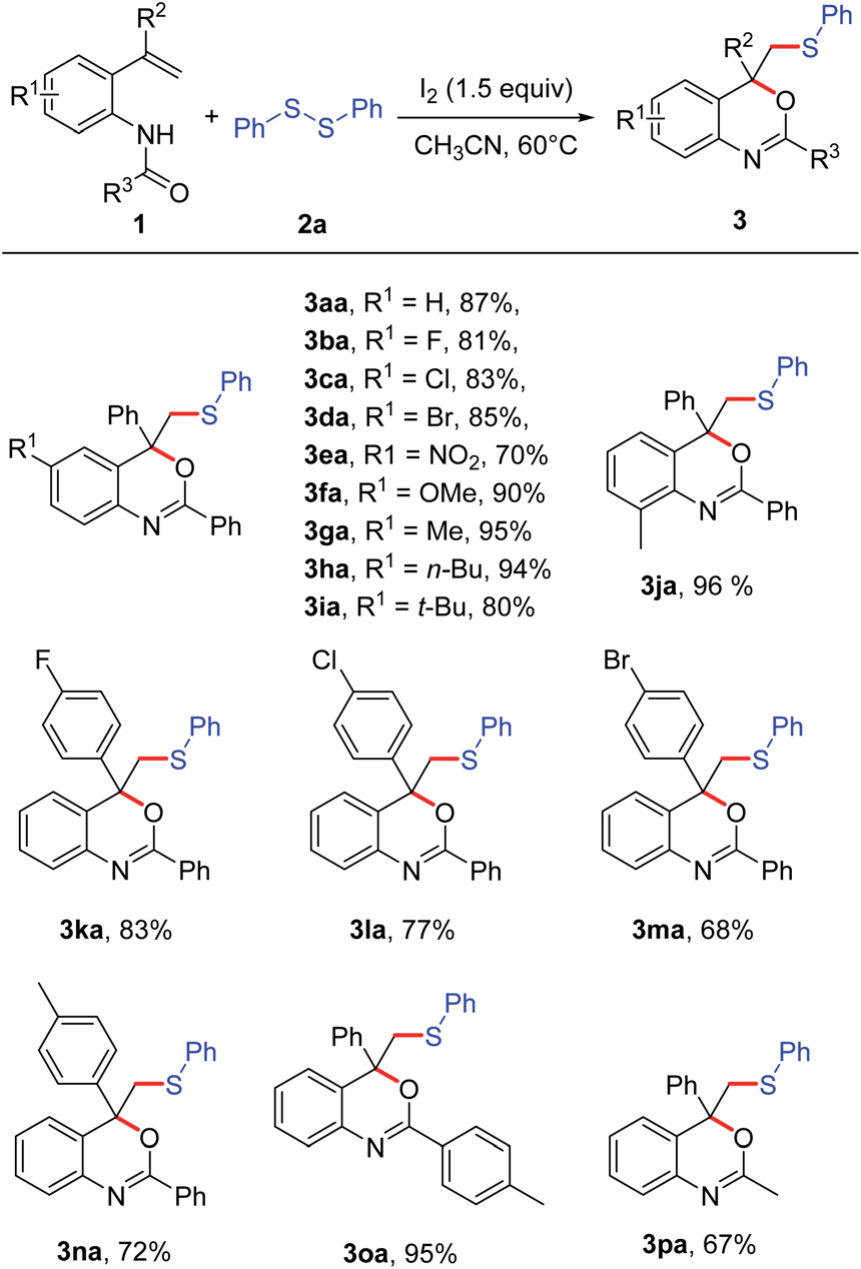
Substrate scope of amide derivatives. The reaction was carried out with 1 (0.2 mmol), 2a (0.75 equiv.), I_2_ (1.5 equiv.), in CH_3_CN (2 mL) at 60 °C for 12 h; isolated yields of all products.

To further study the scope of the reaction, different disulfides were subsequently examined (Scheme 3). Generally, the diaryl disulfides bearing either electro-withdrawing groups or electro-donating groups were all suitable substrates in this reaction, affording the benzoxazine derivatives (3ab–3ai) in good yields to excellent yields (80–96%). Especially, the strong electro-deficient substrate, 1,2-bis(4-nitrophenyl)disulfide, could perform well in this transformation to afford 3ae in 80% yield. It was found that the position of methyl on the aromatic ring affected the yield slightly, which might be attributed to the steric hindrance (3ag–3ai). Furthermore, when dialkyl disulfides were used as the substrates, the corresponding products 3aj and 3ak could be isolated in good yields.

**Scheme 3:**
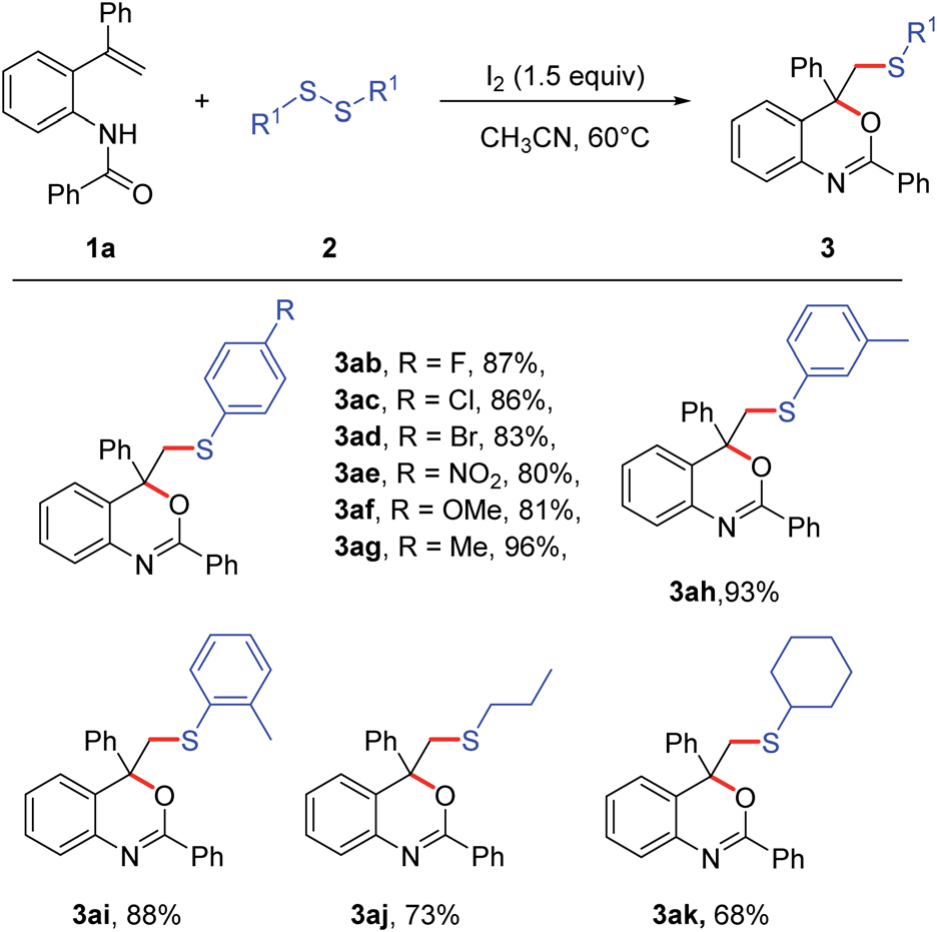
Substrate scope of disulfides. The reaction was carried out with 1a (0.2 mmol), 2 (0.75 equiv.), I_2_ (1.5 equiv.), in CH_3_CN (2 mL) at 60 °C for 12 h; isolated yields of all products.

In order to gain insight into the mechanism of the reaction, several control experiments were conducted (Scheme 4). First, radical trapping experiment was conducted to elucidate whether the reaction involved radical species. When the radical scavenger 2,2,6,6-tetramethyl-1-piperidinyloxy (TEMPO; 2 equiv.) was employed under the standard conditions (Scheme 4a), the yield of 3aa decreased to 45%, implying that part of product were formed involving a free radical process. Iodine was essential in this transformation since 3aa could not be obtained in the absence of I_2_ (Scheme 4b). To figure out the way iodine promoted this reaction, 1a was treated with molecular iodine under standard reaction condition, but no addition product was observed (Scheme 4c). Thus, it is reasonable to hypothesize that the reaction should not involve an iodonium ion intermediate. Further investigation showed that I_2_ could promoted the cleavage and formation of S–S bond. As show in Scheme 4d, mixing 2a and 2g could afford disulfide 5 slowly, with only 5% of product 5 obtained after 1 h. But this process could be accelerated significantly in the present of I_2_, affording 5 with the yields of 50% after 5 minutes.

**Scheme 4:**
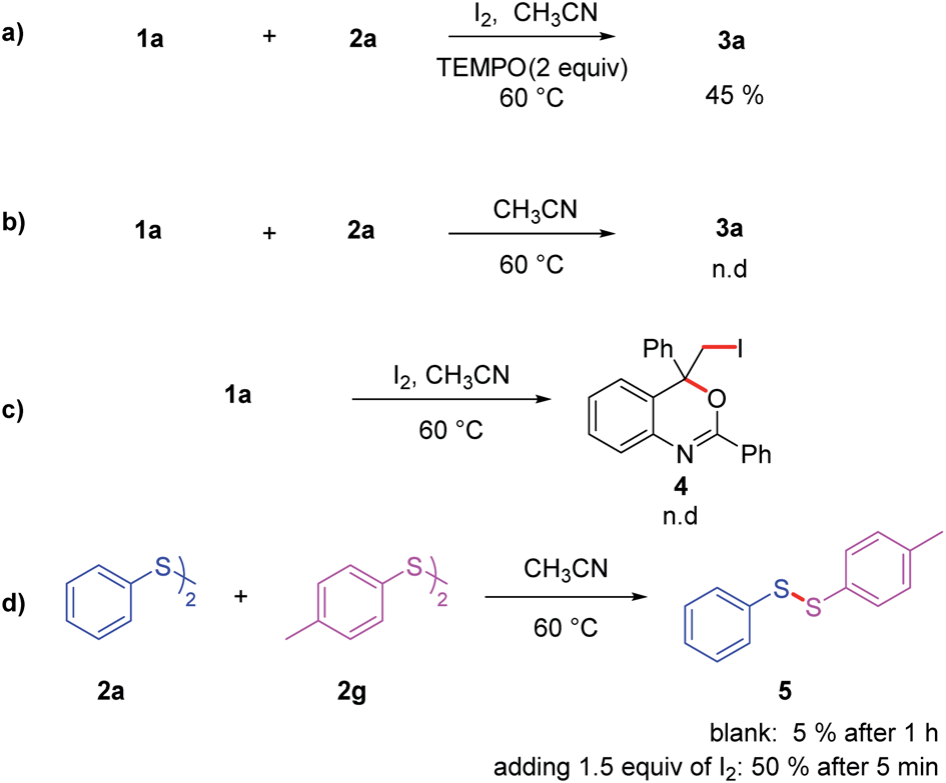
The reaction was carried out in CH_3_CN (2 mL) at 60 °C for 12 h. (a) 1a (0.25 mmol), 2a (2 eqiuv.), I_2_ (2 eqiuv.), TEMPO (2 equiv.); (b) 1a (0.25 mmol), 2a (2 eqiuv.); (c) 1a (0.25 mmol), 2a (2 eqiuv.), I_2_ (2 eqiuv.); (d) 2a (0.25 mmol), 2g (0.25 mmol).

Based on the control experiments and previous studies,^40–52^ two plausible reaction paths were outlined in Scheme 5. In the promotion of I_2_, PhS· and PhSI could be generated *in situ* from 2a. Subsequently, PhS· performed with 1a*via* a radical addition to generate carbon radical intermediate A, which is oxidized into carbon cation intermediate B in path a. Meanwhile, intermediate B also could be generated from the electrophilic addition of 1a and PhSI directly in path b. Finally, an intramolecular nucleophilic attack of B occurs to form intermediate C, followed by loss of proton to afford the thio-tethered benzoxaine 3aa. Additionally, to investigate possible process of path b further, DFT calculation were carried out (Scheme 6). Calculation results show that the BED of S–I bond in PhSI is 31.1 kcal mol^−1^ while the energy barrier (Δ*G*) of the addition between 1a and PhSI is 15.2 kcal mol^−1^. It suggests that PhSI reacting with alkene *via* an electrophilic addition directly is more likely to happen than that of generating PhS· *via* the cleavage of S–I bond in path b. Two paths proposed and computational results agree with the control experimental data that the yield of 3aa decreases by nearly half when the radical scavenger is employed.

**Scheme 5:**
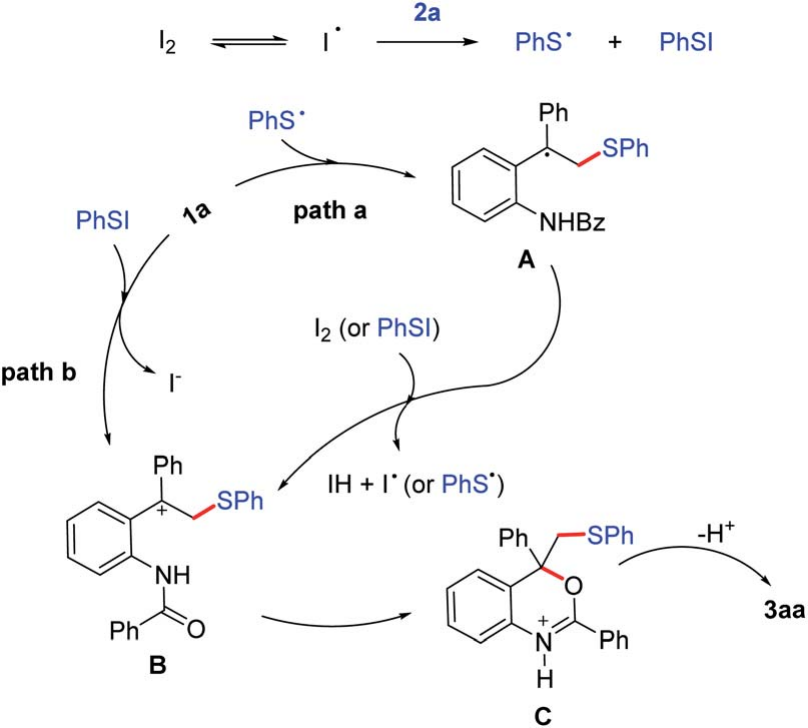
Proposed mechanism.

**Scheme 6:**
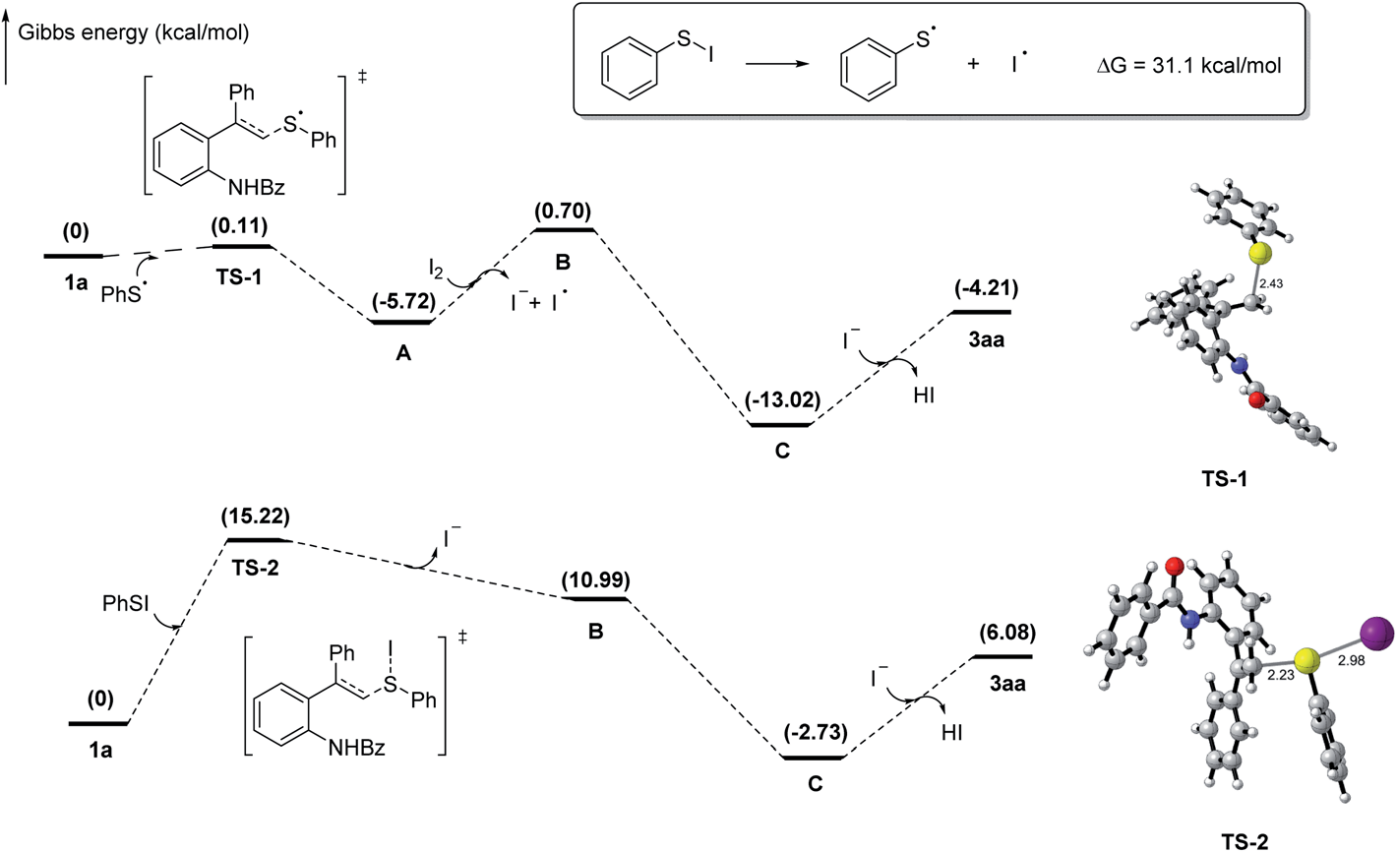
Gibbs energy profiles for proposed mechanism.

## Conclusions

In summary, we developed an efficient synthesis of thio-benzo[*d*][1,3]oxazine derivatives from *o*-vinylanilides and disulfides mediated by iodine. A wide range of substrates were tolerated in this transformation. This reaction can be carried out smoothly under metal-free and mild conditions with a variety of thio-benzo[*d*][1,3]oxazine derivatives obtained in good to excellent yields by virtue of this developed conditions. Research to develop more efficient and green methods along this line is ongoing in our laboratory.

## Conflicts of interest

There are no conflicts to declare.

## Supplementary Material

RA-012-D2RA01078J-s001
