# Supplementary material for: Iodine-mediated oxythiolation of o-vinylanilides with disulfides for the synthesis of benzoxazines
Source: RSC Adv. 2022 Mar 4;12(12):7347–51. doi: 10.1039/d2ra01078j (PMC8982212; doi:10.1039/d2ra01078j)

Electronic Supplementary Material (ESI) for RSC Advances.
This journal is © The Royal Society of Chemistry 2022

Supporting Information

Iodine-mediated oxythiolation of o-vinylanilides with disulfides for the synthesis of benzoxazines

*Yu Yang, ‡ [a,b] Liyan Liu, ‡ [b] Kuiliang Li, [b] Zhenggen Zha, [b] Qi Sun, * [b] and Zhiyong Wang * [b]*

[a] *School of Chemistry and Chemical Engineering, Hefei Normal University, Hefei, 230601, China.*

[b] *Hefei National Laboratory for Physical Sciences at Microscale, CAS Key Laboratory of Soft Matter Chemistry and Department of Chemistry, University of Science and Technology of China, Hefei, Anhui, 230026, P. R. China*

E-mail: zwang3@ustc.edu.cn.

Contents

1. General Information 1
2. General Procedures of this transformation 2
3. Control Experiment 4
4. Characterization Data for the Products 5
5. DFT calculations 19
6. Reference 35
7. NMR Spectra of Product 37

1. General Information

Unless otherwise indicated, all commercial reagents were used without additional purification. All Substances were synthesized according to the previous literature.[1] ^{1}H NMR, ^{19}F NMR and ^{13}C NMR were recorded on a Bruker-400 MHz Spectrometer (^{1}H NMR: 400 MHz, ^{19}F NMR: 376 MHz, ^{13}C NMR: 100 MHz). All chemical shifts (δ) were reported in ppm and coupling constants (*J*) in Hz. All chemical shifts were reported relative to tetramethylsilane (0 ppm for ^{1}H), and $CDCl_3$ (77 ppm for ^{13}C), respectively. HRMS (ESI) were recorded on a Water TM Q-TOF Premier Mass Spectrometer.

2. General Procedures of this transformation

2.1 Optimization of reaction conditions

Table 1. Optimization of the reaction conditions[a].

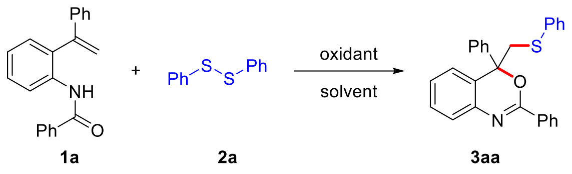

Entry	Oxidant	Solvent	Temp.(°C)	Yield (%)[b]
1	I_2	DCE	50	57
2	PIDA	DCE	50	n.d.[c]
3	PIFA	DCE	50	n.d.
4	I_2	DMSO	50	n.d.
5	I_2	DMF	50	n.d.
6	I_2	CH_3CN	50	63
7	I_2	1,4-dioxane	50	35
8	I_2	EtOH	50	<5
9	I_2	MeOH	50	<5
10	I_2	toluene	50	<5
11	I_2	PhCl	50	<5
12	I_2	H_2O	50	<5
13	I_2	CH_3CN/H_2O=2:1	50	46
14	I_2	CH_3CN/H_2O=1:1	50	38
15	I_2	CH_3CN/H_2O=1:2	50	25
16[d]	I_2	CH_3CN	50	27
17[e]	I_2	CH_3CN	50	45
18[f]	I_2	CH_3CN	50	81
19[g]	I_2	CH_3CN	50	81
20[f]	I_2	CH_3CN	60	87
21[f]	I_2	CH_3CN	70	72
22[f]	I_2	CH_3CN	80	60
22[h]	I_2	CH_3CN	60	<5
23[i]	I_2	CH_3CN	60	<5

24[j]	I_2	CH_3CN	60	<5
25[k]	I_2	DCE	60	64

[a] Reaction condition: **1a** (0.2 mmol, 1 equiv), **2a** (0.1 mmol, 0.5 euqiv), oxidant (0.2 mmol, 1 equiv), 12 h. [b] Isolated yield. [c] n.d. = not detected. [d] **2a** (0.75 equiv) and I_2 (0.2 equiv) were used. [e] **2a** (0.75 equiv) and I_2 (0.5 equiv) were used. [f] **2a** (0.75 equiv) and I_2 (1.5 equiv) were used. [g] **2a** (1 equiv) and I_2 (2 equiv) were used. [h] 0.04 (0.2 equiv) mmol I_2 was used as catalyst and 0.6 mmol (3 equiv) TBHP was used as oxidant. [i] 0.04 (0.2 equiv) mmol I_2 was used as catalyst and 0.6 mmol (3 equiv) H_2O_2 was used as oxidant. [j] 0.04 (0.2 equiv) mmol I_2 was used as catalyst and 0.6 mmol (3 equiv) $K_2S_2O_4$ was used as oxidant. [k] I_2 (1.5 equiv) were used.

Table 2. Optimization of the reaction time[a].

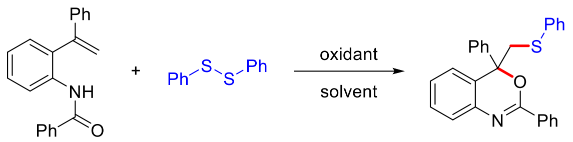

1a **2a** **3aa**

Reaction time（h）	Yield（%）
0.5	9
1	25
2	47
4	65
6	72
8	78
10	84
12	87
18	87
24	87

[a] Reaction condition: **1** (0.2 mmol), **2a** (0.75 equiv), I_2 (1.5 equiv), in CH_3CN (2 mL) at 60 °C.

2.2 General Procedures of this transformation

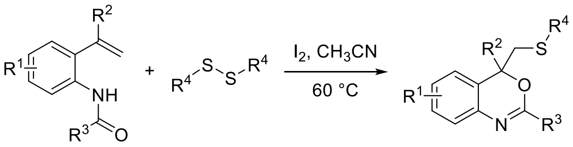

To a 10 mL tube was added *o*-vinylanilide **1** (0.2 mmol), disulfide **2** (0.15 mmol, 0.75 equiv), I_2 (0.3 mmol, 1.5 equiv) and CH_3CN (2 mL). After heating at 60 °C for 12 hours, the reaction was worked up with saturated $Na_2S_2O_3$ solution and extracted with EtOAc (2 mL × 3). The combined organic phase was

washed with brine and dried over anhydrous Na_2SO_4. After the solvent had been completely removed, the residue was purified by column chromatography on silica gel (petroleum ether/ethyl acetate = 20:1 to 6:1) to give the product **3**.

3. Control Experiment

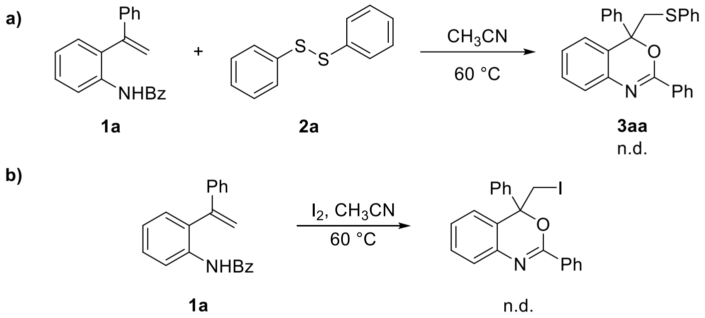

Reaction condition a: **1a** (0.2 mmol, 1 equiv), **2a** (0.2 mmol, 1 equiv), CH_3CN (2 mL), 60 °C, 12 h;

Reaction condition b: **1a** (0.2 mmol, 1 equiv), I_2 (0.3 mmol, 1.5 equiv), CH_3CN (2 mL), 60 °C, 12 h;

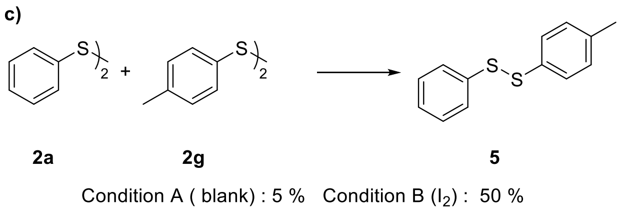

In the absence of iodine, only a small amount of **2a** and **2g** were transformed into mixed disulfide **5** after 1 h, which may be promoted by light or heat. While in the presence of iodine, half of the disulfide compound is the mixed disulfide **5** after 5 min, indicating that iodine can promote the break and formation of the S-S bond.

4. Characterization Data for the Products

2,4-diphenyl-4-((phenylthio)methyl)-4*H*-benzo[*d*][1,3]oxazine (3aa)

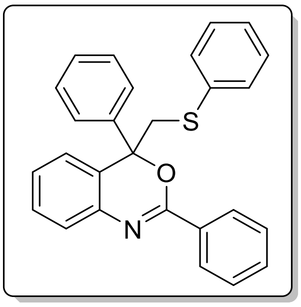

The title compound was prepared according to the general working procedure and purified by column chromatography (PE:EA = 20:1~6:1) to give the product as a colorless oil: 87 % yield (71 mg). ^{1}H NMR (400 MHz, Chloroform-*d*) δ 8.16 – 8.09 (m, 2H), 7.48 – 7.42 (m, 1H), 7.42 – 7.33 (m, 6H), 7.32 – 7.23 (m, 5H), 7.21 – 7.07 (m, 5H), 3.96 (d, *J* = 13.9 Hz, 1H), 3.90 (d, *J* = 13.9 Hz, 1H). ^{13}C NMR (101 MHz, $CDCl_3$) δ 156.1, 141.8, 139.5, 136.7, 132.2, 131.4, 130.3, 129.3, 128.8, 128.3, 128.2, 128.1, 128.0, 126.9, 126.4, 126.3, 126.1, 125.5, 124.8, 83.6, 45.1. HRMS (ESI) m/z calcd for. $C_{27}H_{22}NOS$ $[M+H]^+$ 408.1417, found 408.1435.

6-fluoro-2,4-diphenyl-4-((phenylthio)methyl)-4*H*-benzo[*d*][1,3]oxazine (3ba)

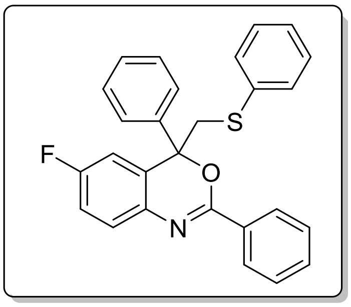

The title compound was prepared according to the general working procedure and purified by column chromatography (PE:EA = 20:1~6:1) to give the product as a colorless oil: 81 % yield (69 mg). ^{1}H NMR (400 MHz, Chloroform-*d*) δ 8.15 – 8.04 (m, 2H), 7.49 – 7.42 (m, 1H), 7.42 – 7.35 (m, 4H), 7.35 – 7.25 (m, 6H), 7.19 – 7.08 (m, 3H), 7.06 – 6.99 (m, 1H), 6.82 (dd, *J* = 8.7, 2.8 Hz, 1H), 3.89 (s, 2H). ^{13}C NMR (101 MHz, $CDCl_3$) δ 161.5 (d, *J*= 246.7 Hz), 155.5 (d, *J* = 1.88 Hz) 141.3, 136.3, 135.8 (d, *J* = 2.78), 132.0, 131.4, 130.5, 128.9, 128.5, 128.4, 128.3, 128.2, 127.9, 127.0 (d, *J* = 8.8 Hz), 126.6, 126.0, 116.0

(d, *J* = 22.0 Hz), 112.0 (d, *J* = 24.8 Hz), 83.3 (d, *J* = 2.0 Hz), 45.1. ^{19}F NMR (376 MHz, $CDCl_3$) δ -114.29. HRMS (ESI) m/z calcd for $C_{27}H_{21}FNOS$ $[M+H]^+$ 426.1323, found 426.1337.

6-chloro-2,4-diphenyl-4-((phenylthio)methyl)-4*H*-benzo[*d*][1,3]oxazine (3ca)

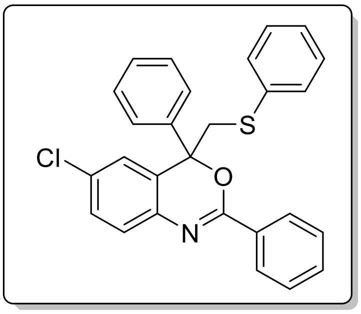

The title compound was prepared according to the general working procedure and purified by column chromatography (PE:EA = 20:1~6:1) to give the product as a colorless oil: 83 % yield (73 mg). ^{1}H NMR (400 MHz, Chloroform-*d*) δ 8.15 – 8.05 (m, 2H), 7.51 – 7.43 (m, 1H), 7.43 – 7.35 (m, 4H), 7.33 – 7.24 (m, 7H), 7.20 – 7.09 (m, 3H), 7.06 – 7.05 (m, 1H), 3.89 (s, 2H). ^{13}C NMR (101 MHz, $CDCl_3$) δ 156.3, 141.2, 138.2, 136.2, 131.8, 131.6, 131.4, 130.6, 129.3, 128.9, 128.5, 128.3, 128.2, 128.0, 126.8, 126.6, 126.0, 125.0, 83.5, 45.2. HRMS (ESI) m/z calcd for $C_{27}H_{21}ClNOS$ $[M+H]^+$ 442.1027, found 442.1036.

6-bromo-2,4-diphenyl-4-((phenylthio)methyl)-4*H*-benzo[*d*][1,3]oxazine (3da)

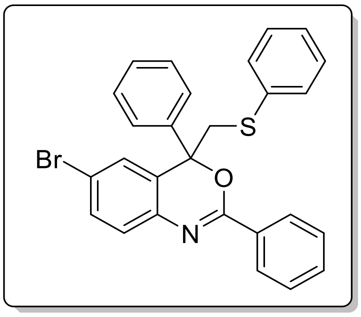

The title compound was prepared according to the general working procedure and purified by column chromatography (PE:EA = 20:1~6:1) to give the product as a colorless oil: 85 % yield (82 mg). ^{1}H NMR (400 MHz, Chloroform-*d*) δ 8.11 – 8.09 (m, 2H), 7.49 – 7.47 (m, 1H), 7.46 (dd, *J* = 8.4, 2.2 Hz, 1H), 7.44 – 7.40 (m, 4H), 7.39 – 7.30 (m, 5H), 7.28 (d, *J* = 7.5 Hz, 1H), 7.25 – 7.13 (m, 4H), 3.90 (s, 2H). ^{13}C NMR (101 MHz, $CDCl_3$) δ 156.5, 141.2, 138.6, 136.2, 132.4, 131.8, 131.7, 130.7, 128.9, 128.7, 128.6,

128.3, 128.1, 127.9, 127.1, 126.7, 126.0, 119.3, 83.5, 45.3. HRMS (ESI) m/z calcd for $C_{27}H_{21}BrNOS$ $[M+H]^+$ 486.0522, found 486.0525.

6-nitro-2,4-diphenyl-4-((phenylthio)methyl)-4*H*-benzo[*d*][1,3]oxazine (3ea)

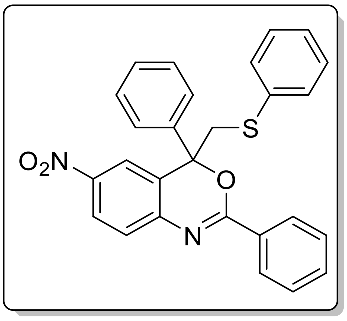

The title compound was prepared according to the general working procedure and purified by column chromatography (PE:EA = 20:1~6:1) to give the product as a colorless oil: 70 % yield (63 mg). ^{1}H NMR (400 MHz, $CDCl_3$) δ 8.10 (d, *J* = 7.6 Hz, 2H), 7.47 (t, *J* = 7.2 Hz, 1H), 7.43 – 7.32 (m, 6H), 7.31 – 7.10 (m, 9H), 3.93 (d, *J* = 13.9 Hz, 1H), 3.84 (d, *J* = 13.9 Hz, 1H). ^{13}C NMR (101 MHz, $CDCl_3$) δ 156.0, 140.9, 139.5, 136.5, 132.1, 131.6, 131.5, 130.5, 129.6, 129.0, 128.4, 128.1, 126.7, 126.6, 125.8, 124.7, 122.6, 83.2, 45.0. HRMS (ESI) m/z calcd for $C_{27}H_{21}N_2O_3S$ $[M+H]^+$ 453.1268, found 453.1602.

6-methoxy-2,4-diphenyl-4-((phenylthio)methyl)-4*H*-benzo[*d*][1,3]oxazine (3fa)

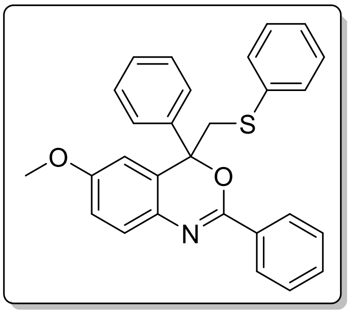

The title compound was prepared according to the general working procedure and purified by column chromatography (PE:EA = 20:1~6:1) to give the product as a colorless oil: 90 % yield (78 mg). ^{1}H NMR (400 MHz, Chloroform-*d*) δ 8.18 – 8.02 (m, 2H), 7.46 – 7.34 (m, 5H), 7.33 – 7.20 (m, 6H), 7.19 – 7.12 (m, 2H), 7.12 – 7.06 (m, 1H), 6.92 – 6.82 (m, 1H), 6.67 (dd, *J* = 2.8, 1.0 Hz, 1H), 3.96 – 3.86 (m, 2H), 3.73 (s, 3H). ^{13}C NMR (101 MHz, $CDCl_3$) δ 158.0, 154.4, 141.7, 136.7, 133.1, 132.3, 131.0, 130.3, 128.8,

128.3, 128.2, 128.1, 128.0, 127.7, 126.6, 126.3, 126.0, 113.6, 111.1, 83.4, 55.4, 45.1. HRMS (ESI) m/z calcd for $C_{28}H_{24}NO_2S$ $[M+H]^+$ 438.15222, found 426.1543.

6-methyl-2,4-diphenyl-4-((phenylthio)methyl)-4*H*-benzo[*d*][1,3]oxazine (3ga)

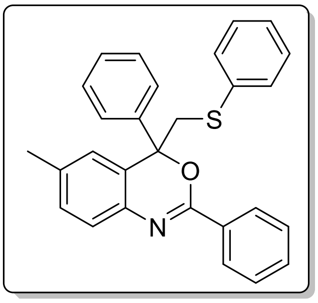

The title compound was prepared according to the general working procedure and purified by column chromatography (PE:EA = 20:1~6:1) to give the product as a colorless oil: 95 % yield (80 mg). ^{1}H NMR (400 MHz, Chloroform-*d*) δ 8.17 – 8.05 (m, 2H), 7.47 – 7.34 (m, 5H), 7.33 – 7.20 (m, 6H), 7.19 – 7.05 (m, 4H), 6.89 (d, *J* = 1.9 Hz, 1H), 3.94 (d, *J* = 13.9 Hz, 1H), 3.89 (d, *J* = 13.9 Hz, 1H), 2.30 (s, 3H). ^{13}C NMR (101 MHz, $CDCl_3$) δ 155.4, 142.0, 137.1, 136.8, 136.2, 132.3, 131.2, 130.3, 129.9, 128.8, 128.3, 128.2, 128.1, 127.8, 126.6, 126.3, 126.3, 126.1, 125.3, 83.6, 45.2, 21.4. HRMS (ESI) m/z calcd for $C_{28}H_{24}NOS$ $[M+H]^+$ 442.1573, found 442.1598.

6-butyl-2,4-diphenyl-4-((phenylthio)methyl)-4*H*-benzo[*d*][1,3]oxazine (3ha)

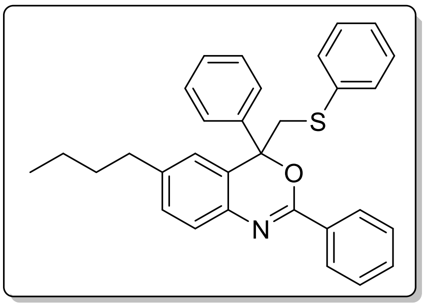

The title compound was prepared according to the general working procedure and purified by column chromatography (PE:EA = 20:1~6:1) to give the product as a yellow oil: 94 % yield (87 mg). ^{1}H NMR (400 MHz, Chloroform-*d*) δ 8.07 – 7.97 (m, 2H), 7.39 – 7.33 (m, 1H), 7.33 – 7.27 (m, 4H), 7.23 – 7.13 (m, 6H), 7.11 – 7.05 (m, 3H), 7.05 – 6.99 (m, 1H), 6.84 (s, 1H), 3.88 (d, *J* = 13.8 Hz, 1H), 3.82 (d, *J* = 12.8 Hz, 1H), 2.49 (t, *J* = 7.8 Hz, 2H), 1.55 – 1.42 (m, 2H), 1.31- 1.20 (m, 2H), 0.83 (t, *J* = 7.3 Hz, 3H).

^{13}C NMR (101 MHz, $CDCl_3$) δ 155.4, 142.0, 141.3, 137.3, 136.9, 132.4, 131.1, 130.3, 129.2, 128.8, 128.3, 128.2, 128.1, 127.9, 126.5, 126.3, 126.1, 125.3, 124.7, 82.6, 45.2, 35.5, 33.5, 22.3, 13.9. HRMS (ESI) m/z calcd for $C_{31}H_{30}NOS$ $[M+H]^+$ 464.2043, found 464.2035.

6-(tert-butyl)-2,4-diphenyl-4-((phenylthio)methyl)-4*H*-benzo[*d*][1,3]oxazine (3ia)

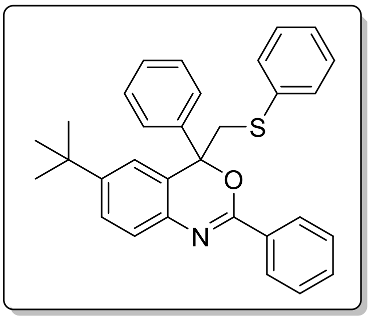

The title compound was prepared according to the general working procedure and purified by column chromatography (PE:EA = 20:1~6:1) to give the product as a colorless oil: 80 % yield (74 mg). ^{1}H NMR (400 MHz, Chloroform-*d*) δ 8.12 (d, *J* = 6.7 Hz, 2H), 7.45 – 7.41 (m, 1H), 7.41 – 7.35 (m, 5H), 7.33 – 7.23 (m, 6H), 7.19 – 7.12 (m, 3H), 7.12 – 7.05 (m, 1H), 3.98 (d, *J* = 13.8 Hz, 1H), 3.94 (s, 1H), 1.28 (s, 9H). ^{13}C NMR (101 MHz, $CDCl_3$) 155.6, 149.5, 142.0, 137.1, 136.9, 132.4, 131.2, 130.3, 128.8, 128.3, 128.2, 128.1, 127.9, 126.3, 126.2, 126.1, 126.0, 125.0, 121.7, 83.8, 45.25, 34.7, 31.3. HRMS (ESI) m/z calcd for $C_{31}H_{30}NOS$ $[M+H]^+$ 464.2043, found 464.2035.

8-methyl-2,4-diphenyl-4-((phenylthio)methyl)-4*H*-benzo[*d*][1,3]oxazine (3ja)

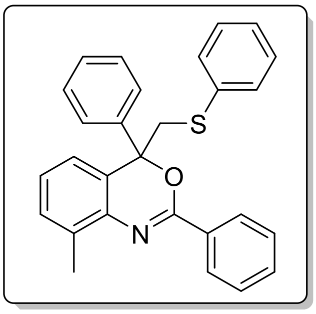

The title compound was prepared according to the general working procedure and purified by column chromatography (PE:EA = 20:1~6:1) to give the product as a colorless oil: 96 % yield (81 mg). ^{1}H NMR (400 MHz, Chloroform-*d*) δ 8.16 (d, *J* = 8.2, 1.5 Hz, 2H), 7.50 – 7.44 (m, 1H), 7.43 – 7.37 (m, 4H), 7.34 – 7.29 (m, 2H), 7.29 – 7.21 (m, 4H), 7.19 (t, *J* = 7.6 Hz, 2H), 7.15 – 7.08 (m, 2H), 7.01 (d, *J* = 7.6 Hz,

1H), 3.96 (d, J = 13.8 Hz, 1H), 3.89 (d, J = 13.8 Hz, 1H), 2.53 (s, 3H). ^{13}C NMR (101 MHz, $CDCl_3$) δ 155.0, 142.0, 137.7, 136.9, 134.1, 132.6, 131.2, 130.6, 130.2, 128.8, 128.3, 128.2, 128.0, 126.8, 126.3, 126.1, 125.7, 122.3, 83.5, 45.1, 17.2. HRMS (ESI) m/z calcd for $C_{28}H_{24}NOS$ $[M+H]^+$ 442.1573, found 442.1598.

4-(4-fluorophenyl)-2-phenyl-4-((phenylthio)methyl)-4*H*-benzo[*d*][1,3]oxazine (3ka)

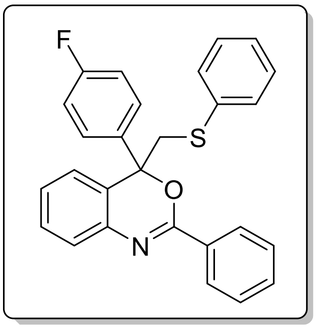

The title compound was prepared according to the general working procedure and purified by column chromatography (PE:EA = 20:1~6:1) to give the product as a colorless oil: 83 % yield (70 mg). ^{1}H NMR (400 MHz, Chloroform-*d*) δ 8.10 (d, J = 7.8 Hz, 2H), 7.46 (d, J = 7.3 Hz, 1H), 7.43 – 7.32 (m, 6H), 7.29 (d, J = 7.5 Hz, 2H), 7.23 – 7.10 (m, 5H), 6.95 (t, J = 8.6 Hz, 2H), 3.94 (d, J = 13.9 Hz, 1H), 3.87 (d, J = 13.9 Hz, 1H). ^{13}C NMR (101 MHz, $CDCl_3$) δ 162.4(d, J = 248.9 Hz), 156.01, 139.40, 137.60 (d, J = 3.1 Hz), 136.45, 132.03, 131.50, 130.34, 129.44, 128.86, 128.23, 128.14, 128.05, 127.95, 126.76, 126.49, 125.59, 124.63, 115.2 (d, J = 21.6 Hz), 83.10, 45.11. ^{19}F NMR (376 MHz, $CDCl_3$) δ -113.62. HRMS (ESI) m/z calcd for $C_{27}H_{21}FNOS$ $[M+H]^+$ 426.1323, found 426.1337.

4-(4-chlorophenyl)-2-phenyl-4-((phenylthio)methyl)-4*H*-benzo[*d*][1,3]oxazine (3ma)

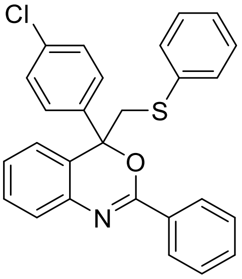

The title compound was prepared according to the general working procedure and purified by column chromatography (PE:EA = 20:1~6:1) to give the product as a colorless oil: 77 % yield (68 mg). ^{1}H NMR (400 MHz, Chloroform-*d*) δ 8.10 (d, J = 7.6 Hz, 2H), 7.51 – 7.45 (m, 1H), 7.44 – 7.36 (m, 4H), 7.34 –

7.23 (m, 7H), 7.18 (t, *J* = 7.6 Hz, 2H), 7.13 (t, *J* = 7.3 Hz, 2H), 3.94 (d, *J* = 13.9 Hz, 1H), 3.86 (d, *J* = 13.9 Hz, 1H). ^{13}C NMR (101 MHz, $CDCl_3$) δ 156.0, 140.2, 139.3, 136.4, 134.3, 131.9, 131.6, 130.4, 129.5, 128.9, 128.5, 128.3, 128.0, 127.7, 126.6, 126.5, 125.6, 124.6, 83.2, 45.0. HRMS (ESI) m/z calcd for $C_{27}H_{21}ClNOS$ $[M+H]^+$ 442.1027, found 464.0846.

4-(4-bromophenyl)-2-phenyl-4-((phenylthio)methyl)-4H-benzo[d][1,3]oxazine (3na)

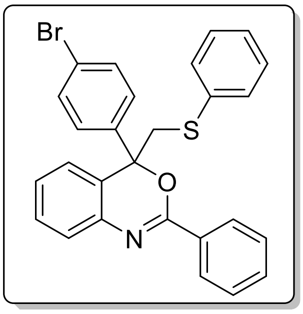

The title compound was prepared according to the general working procedure and purified by column chromatography (PE:EA = 20:1~6:1) to give the product as a colorless oil: 68 % yield (66 mg). ^{1}H NMR (400 MHz, Chloroform-*d*) δ 8.19 – 8.08 (m, 3H), 7.94 (s, 1H), 7.52 (t, *J* = 7.4 Hz, 1H), 7.46 – 7.38 (m, 3H), 7.38 – 7.28 (m, 5H), 7.27 – 7.22 (m, 2H), 7.17 – 7.07 (m, 3H), 3.97 (s, 2H). ^{13}C NMR (101 MHz, $CDCl_3$) δ 158.9, 145.4, 145.2 141.0, 135.6, 132.5, 131.2, 130.9, 128.9, 128.9, 128.8, 128.5, 128.4, 127.2, 126.9, 126.0, 125.7, 124.9, 121.2, 84.2, 45.6. HRMS (ESI) m/z calcd for $C_{27}H_{21}BrNOS$ $[M+H]^+$ 486.0522, found 486.0525.

2-phenyl-4-((phenylthio)methyl)-4-(p-tolyl)-4*H*-benzo[*d*][1,3]oxazine (3oa)

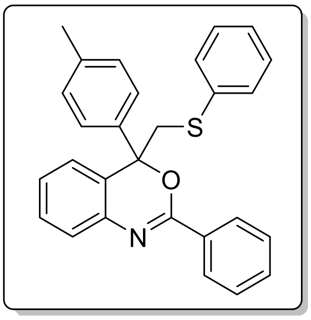

The title compound was prepared according to the general working procedure and purified by column chromatography (PE:EA = 20:1~6:1) to give the product as a colorless oil: 72 % yield (61 mg). ^{1}H NMR (400 MHz, Chloroform-*d*) δ 8.11 (d, J = 8.1 Hz, 2H), 7.47 – 7.41 (m, 1H), 7.41 – 7.32 (m, 4H), 7.30 –

7.25 (m, 4H), 7.22 – 7.04 (m, 7H), 3.94 (d, J = 13.9 Hz, 1H), 3.89 (d, J = 13.9 Hz, 1H), 2.27 (s, 3H). 13C NMR (101 MHz, CDCl3) δ 156.2, 139.5, 139.0, 138.1, 136.7, 132.2, 131.3, 130.2, 129.2, 129.0, 128.8, 128.1, 128.0, 127.1, 126.3, 126.3, 126.0, 125.4, 124.8, 83.5, 45.0, 21.0. HRMS (ESI) m/z calcd for $C_{28}H_{24}NOS$ $[M+H]^+$ 442.1573, found 442.1556.

4-phenyl-4-((phenylthio)methyl)-2-(p-tolyl)-4*H*-benzo[*d*][1,3]oxazine (3pa)

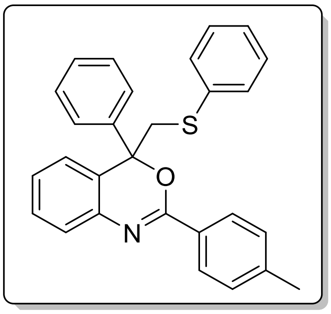

The title compound was prepared according to the general working procedure and purified by column chromatography (PE:EA = 20:1~6:1) to give the product as a colorless oil: 95 % yield (80 mg). ^{1}H NMR (400 MHz, Chloroform-*d*) δ 8.02 (d, *J* = 8.0 Hz, 2H), 7.38 (d, *J* = 6.8 Hz, 2H), 7.35 – 7.31 (m, 2H), 7.31 – 7.23 (m, 5H), 7.21 – 7.07 (m, 7H), 3.95 (d, *J* = 13.8 Hz, 1H), 3.88 (d, *J* = 13.8 Hz, 1H), 2.37 (s, 3H). ^{13}C NMR (101 MHz, $CDCl_3$) δ 156.3, 141.8, 141.7, 139.6, 136.7, 130.2, 129.4, 129.2, 128.9, 128.8, 128.3, 128.2, 128.0, 126.9, 126.3, 126.1, 126.0, 125.3, 124.7, 83.4, 45.0, 21.6. HRMS (ESI) m/z calcd for $C_{28}H_{24}NOS$ $[M+H]^+$ 442.1573, found 442.1598.

2-methyl-4-phenyl-4-((phenylthio)methyl)-4*H*-benzo[*d*][1,3]oxazine (3qa)

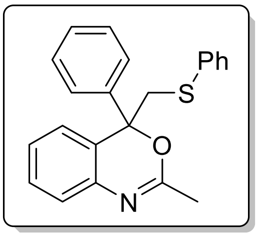

The title compound was prepared according to the general working procedure and purified by column chromatography (PE:EA = 20:1~6:1) to give the product as a colorless oil: 67 % yield (46 mg). ^{1}H NMR (400 MHz, Chloroform-*d*) δ 7.38 – 7.26 (m, 8H), 7.23 (t, *J* = 7.0 Hz, 2H), 7.20 – 7.11 (m, 3H), 7.01 (d, *J* = 7.6 Hz, 1H), 3.82 (s, 2H), 2.03 (s, 3H). ^{13}C NMR (101 MHz, $CDCl_3$) δ 159.6, 142.3, 138.8,

136.6, 130.2, 129.2, 128.8, 128.4, 128.3, 126.4, 126.1, 126.0, 125.9, 125.0, 124.5, 83.2, 45.1, 21.5. HRMS (ESI) m/z calcd for $C_{22}H_{20}NOS$ $[M+H]^+$ 346.1260, found 346.1266.

4-(((4-fluorophenyl)thio)methyl)-2,4-diphenyl-4*H*-benzo[*d*][1,3]oxazine (3ab)

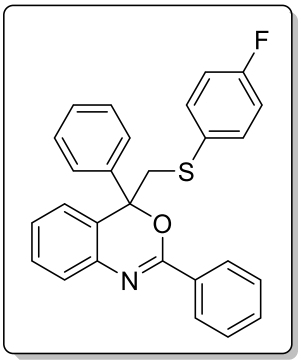

The title compound was prepared according to the general working procedure and purified by column chromatography (PE:EA = 20:1~6:1) to give the product as a colorless oil: 87 % yield (74 mg). ^{1}H NMR (400 MHz, Chloroform-*d*) δ 8.10 (d, *J* = 7.6 Hz, 2H), 7.47 (t, *J* = 7.3 Hz, 1H), 7.43 – 7.33 (m, 6H), 7.31 – 7.21 (m, 5H), 7.22 – 7.16 (m, 1H), 7.09 (d, *J* = 7.6 Hz, 1H), 6.83 (t, *J* = 8.5 Hz, 2H), 3.90 (d, *J* = 14.1 Hz, 1H), 3.84 (d, *J* = 14.1 Hz, 1H). ^{13}C NMR (101 MHz, $CDCl_3$) δ 161.9 (d, *J* = 247.1 Hz), 155.98, 141.87, 139.45, 133.39 (d, *J* = 8.1 Hz), 132.11, 131.55 (d, *J* = 3.5 Hz), 131.44, 129.27, 128.37, 128.26, 128.18, 127.90, 126.74, 126.39, 126.02, 125.54, 124.76, 115.86 (d, *J* = 21.9 Hz), 83.73, 46.45. ^{19}F NMR (376 MHz, $CDCl_3$) δ -114.97. HRMS (ESI) m/z calcd for $C_{27}H_{20}FNOS$ $[M+H]^+$ 426.1323, found 426.1337.

4-(((4-chlorophenyl)thio)methyl)-2,4-diphenyl-4*H*-benzo[*d*][1,3]oxazine (3ac)

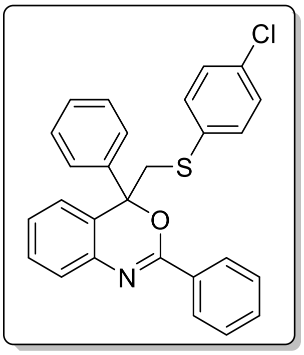

The title compound was prepared according to the general working procedure and purified by column chromatography (PE:EA = 20:1~6:1) to give the product as a colorless oil: 86 % yield (76 mg). 1H NMR

(400 MHz, Chloroform-d) *δ* 7.98 (d, *J* = 7.7 Hz, 2H), 7.38 (t, *J* = 7.3 Hz, 1H), 7.34 – 7.23 (m, 6H), 7.23 – 7.15 (m, 3H), 7.11 – 7.09 (m, 3H), 7.00 (d, *J* = 8.4 Hz, 3H), 3.82 (d, *J* = 14.1 Hz, 1H), 3.76 (d, *J* = 14.0 Hz, 1H). 13C NMR (101 MHz, CDCl3) δ 155.9, 141.8, 139.4, 135.1, 132.5, 132.0, 131.8, 131.4, 129.3, 128.9, 128.4, 128.3, 128.2, 127.8, 126.7, 126.4, 126.0, 125.6, 124.7, 83.6, 45.4. HRMS (ESI) m/z calcd for $C_{27}H_{20}ClNOS$ $[M+H]^+$ 442.1027, found 442.1036.

4-(((4-bromophenyl)thio)methyl)-2,4-diphenyl-4*H*-benzo[*d*][1,3]oxazine (3ad)

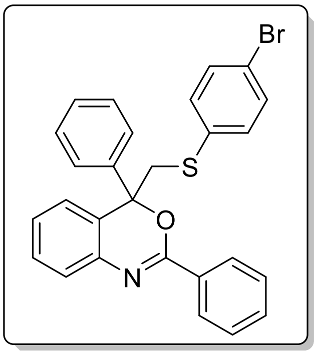

The title compound was prepared according to the general working procedure and purified by column chromatography (PE:EA = 20:1~6:1) to give the product as a colorless oil: 83 % yield (80 mg).. ^{1}H NMR (400 MHz, $CDCl_3$) δ 8.22 – 8.08 (m, 3H), 7.95 (s, 1H), 7.53 (t, *J* = 7.3 Hz, 1H), 7.49 – 7.39 (m, 3H), 7.39 – 7.29 (m, 5H), 7.29 – 7.24 (m, 2H), 7.19 – 7.04 (m, 3H), 3.97 (s, 2H). ^{13}C NMR (101 MHz, $CDCl_3$) δ 159.0, 145.4, 145.2, 141.0, 135.6, 132.5, 131.2, 130.9, 129.0, 128.9, 128.8, 128.5, 128.4, 127.2, 126.9, 126.0, 125.7, 124.9, 121.2, 84.2, 45.6. HRMS (ESI) m/z calcd for $C_{27}H_{21}BrNOS$ $[M+H]^+$ 486.0522, found 486.0525.

4-(((4-nitrophenyl)thio)methyl)-2,4-diphenyl-4*H*-benzo[*d*][1,3]oxazine (3ae)

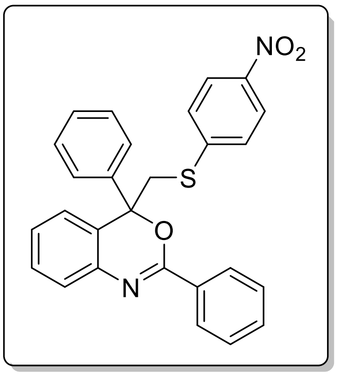

The title compound was prepared according to the general working procedure and purified by column chromatography (PE:EA = 20:1~6:1) to give the product as a yellow oil: 80 % yield (72 mg). ^{1}H NMR (400 MHz, Chloroform-*d*) δ 8.11 – 8.03 (m, 2H), 8.00 – 7.91 (m, 2H), 7.47 – 7.40 (m, 3H), 7.39 – 7.26 (m, 9H), 7.23 – 7.18 (m, 1H), 7.15 – 7.09 (m, 1H), 4.00 (s, 2H). ^{13}C NMR (101 MHz, $CDCl_3$) δ 155.7, 146.6, 145.2, 141.2, 139.2, 131.8, 131.7, 129.6, 128.6, 128.5, 128.2, 127.8, 127.5, 126.6, 126.4, 126.0, 125.7, 124.6, 123.7, 83.1, 42.9. HRMS (ESI) m/z calcd for $C_{27}H_{21}N_2O_3S$ $[M+H]^+$ 453.1268, found 453.1297.

4-(((4-methoxyphenyl)thio)methyl)-2,4-diphenyl-4*H*-benzo[*d*][1,3]oxazine (3af)

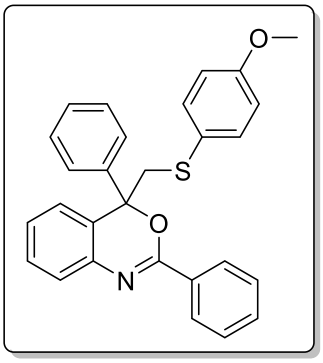

The title compound was prepared according to the general working procedure and purified by column chromatography (PE:EA = 20:1~6:1) to give the product as a colorless oil: 81 % yield (71 mg). ^{1}H NMR (400 MHz, Chloroform-*d*) δ 8.17 – 8.04 (m, 2H), 7.50 – 7.44 (m, 1H), 7.43 – 7.33 (m, 6H), 7.29 – 7.22 (m, 5H), 7.22 – 7.16 (m, 1H), 7.13 – 7.07 (m, 1H), 6.72 – 6.57 (m, 2H), 3.86 (d, *J* = 14.0 Hz, 1H), 3.80 (d, *J* = 14.1 Hz, 1H), 3.68 (s, 3H). ^{13}C NMR (101 MHz, $CDCl_3$) δ 158.0, 155.2, 141.1, 138.4, 132.9,

131.2, 130.4, 128.2, 127.3, 127.2, 127.1, 127.0, 125.9, 125.9, 125.4, 125.0, 124.4, 123.9, 113.4, 83.0, 54.2, 46.2. HRMS (ESI) m/z calcd for $C_{28}H_{24}NO_2S$ $[M+H]^+$ 438.1522, found 438.1543.

2,4-diphenyl-4-((p-tolylthio)methyl)-4*H*-benzo[*d*][1,3]oxazine (3ag)

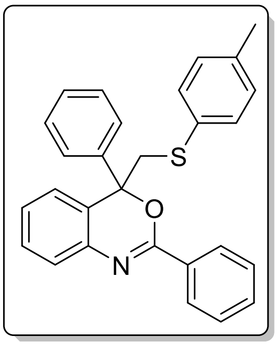

The title compound was prepared according to the general working procedure and purified by column chromatography (PE:EA = 20:1~6:1) to give the product as a colorless oil: 96 % yield (81 mg). Colorless oil. ^{1}H NMR (400 MHz, Chloroform-*d*) δ 8.16 – 8.06 (m, 2H), 7.48 – 7.42 (m, 1H), 7.41 – 7.32 (m, 6H), 7.30 – 7.22 (m, 3H), 7.22 – 7.16 (m, 3H), 7.15 – 7.09 (m, 1H), 6.99 – 6.90 (m, 2H), 3.91 (d, *J* = 13.9 Hz, 1H), 3.86 (d, *J* = 14.0 Hz, 1H), 2.23 (s, 3H). ^{13}C NMR (101 MHz, $CDCl_3$) δ 156.1, 142.0, 139.5, 136.5, 133.0, 132.2, 131.3, 131.0, 129.6, 129.2, 128.3, 128.2, 128.1, 129.0, 126.9, 126.3, 126.0, 125.4, 124.8, 83.7, 45.8, 20.9. HRMS (ESI) m/z calcd for $C_{28}H_{23}NOS$ $[M+H]^+$ 422.1573, found 422.1598.

2,4-diphenyl-4-((m-tolylthio)methyl)-4*H*-benzo[*d*][1,3]oxazine (3ah)

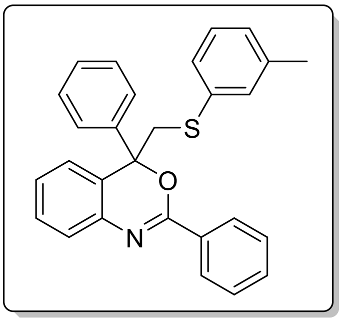

The title compound was prepared according to the general working procedure and purified by column chromatography (PE:EA = 20:1~6:1) to give the product as a colorless oil: 93 % yield (78 mg). ^{1}H NMR (400 MHz, Chloroform-*d*) δ 8.13 (d, *J* = 8.0 Hz, 2H), 7.45 (t, *J* = 7.2 Hz, 1H), 7.42 – 7.31 (m, 6H), 7.30 – 7.22 (m, 3H), 7.22 – 7.15 (m, 1H), 7.15 – 7.01 (m, 4H), 6.90 (d, *J* = 7.3 Hz, 1H), 3.94 (d, *J* = 14.0 Hz, 1H), 3.89 (d, *J* = 13.9 Hz, 1H), 2.17 (s, 3H). ^{13}C NMR (101 MHz, $CDCl_3$) δ 156.1, 141.9, 139.5, 138.6,

136.3, 132.2, 131.3, 131.1, 129.2, 128.6, 128.3, 128.2, 128.1, 128.0, 127.4, 127.3, 126.9, 126.4, 126.1, 125.5, 124.8, 83.6, 45.2, 21.2. HRMS (ESI) m/z calcd for $C_{28}H_{23}NOS$ $[M+H]^+$ 422.1573, found 422.1598.

2,4-diphenyl-4-((o-tolylthio)methyl)-4*H*-benzo[*d*][1,3]oxazine (3ai)

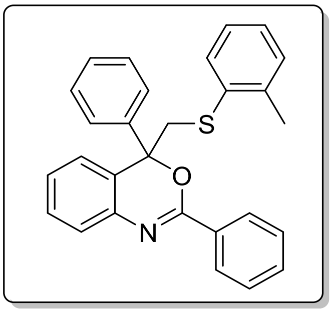

The title compound was prepared according to the general working procedure and purified by column chromatography (PE:EA = 20:1~6:1) to give the product as a colorless oil: 88 % yield (74 mg). ^{1}H NMR (400 MHz, Chloroform-*d*) δ 8.13 (d, *J* = 8.1 Hz, 2H), 7.47 (t, *J* = 7.2 Hz, 1H), 7.43 – 7.34 (m, 6H), 7.32 – 7.19 (m, 5H), 7.13 (d, *J* = 7.7 Hz, 1H), 7.08 – 7.02 (m, 3H), 3.90 (d, *J* = 13.7 Hz, 1H), 3.84 (d, *J* = 13.7 Hz, 1H), 2.25 (s, 3H). ^{13}C NMR (101 MHz, $CDCl_3$) δ 156.2, 141.9, 139.5, 138.9, 135.9, 132.2, 131.4, 130.5, 130.1, 129.2, 128.3 128.2, 128.1, 128.0, 126.8, 126.5, 126.4, 126.3, 126.0, 125.5, 124.9, 83.7, 44.8, 20.6. HRMS (ESI) m/z calcd for $C_{28}H_{23}NOS$ $[M+H]^+$ 422.1573, found 422.1598.

2,4-diphenyl-4-((propylthio)methyl)-4*H*-benzo[*d*][1,3]oxazine (3aj)

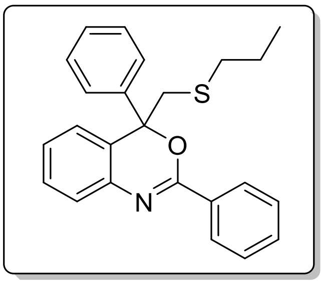

The title compound was prepared according to the general working procedure and purified by column chromatography (PE:EA = 20:1~6:1) to give the product as a colorless oil: 73 % yield (54 mg). ^{1}H NMR (400 MHz, $CDCl_3$) δ 8.26 (d, *J* = 7.3 Hz, 2H), 7.56 – 7.39 (m, 5H), 7.36 (d, *J* = 4.0 Hz, 2H), 7.33 – 7.19 (m, 5H), 7.13 (d, *J* = 7.6 Hz, 1H), 3.57 – 3.41 (m, 2H), 2.52 – 2.33 (m, 2H), 1.62 – 1.43 (m, 2H), 0.86 (t, *J* = 7.3 Hz, 3H). ^{13}C NMR (101 MHz, $CDCl_3$) δ 156.2, 142.2, 139.5, 132.4, 131.5, 129.1, 128.3, 128.2,

128.0, 127.4, 126.3, 126.2, 125.4, 124.9, 84.1, 42.2, 36.2, 22.9, 13.3. HRMS (ESI) m/z calcd for $C_{24}H_{24}NOS$ [M+H]$^+$ 374.1573, found 374.1577.

4-((cyclohexylthio)methyl)-2,4-diphenyl-4*H*-benzo[*d*][1,3]oxazine (3ak)

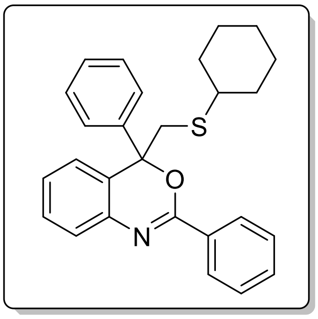

The title compound was prepared according to the general working procedure and purified by column chromatography (PE:EA = 20:1~6:1) to give the product as a colorless oil: 68 % yield (56 mg). ^{1}H NMR (400 MHz, $CDCl_3$) δ 8.26 (d, *J* = 7.5 Hz, 2H), 7.53 – 7.39 (m, 5H), 7.38 – 7.25 (m, 5H), 7.20 (t, *J* = 6.9 Hz, 1H), 7.10 (d, *J* = 7.6 Hz, 1H), 3.50 (s, 2H), 2.50 (t, *J* = 10.6 Hz, 1H), 1.92 (d, *J* = 12.4 Hz, 1H), 1.83 (d, *J* = 12.5 Hz, 1H), 1.65 (t, *J* = 12.4 Hz, 2H), 1.57 – 1.45 (m, 1H), 1.31 – 1.08 (m, 5H). ^{13}C NMR (101 MHz, $CDCl_3$) δ 156.3, 142.1, 139.4, 132.4, 131.5, 129.1, 128.3, 128.1, 128.0, 127.6, 126.3, 125.3, 125.0, 83.9, 45.1, 39.9, 33.6, 33.5, 26.0, 25.9, 25.6. HRMS (ESI) m/z calcd for $C_{27}H_{28}NOS$ [M+H]$^+$ 414.1886, found 414.1887.

5. DFT calculations

5.1 Gibbs energy profiles

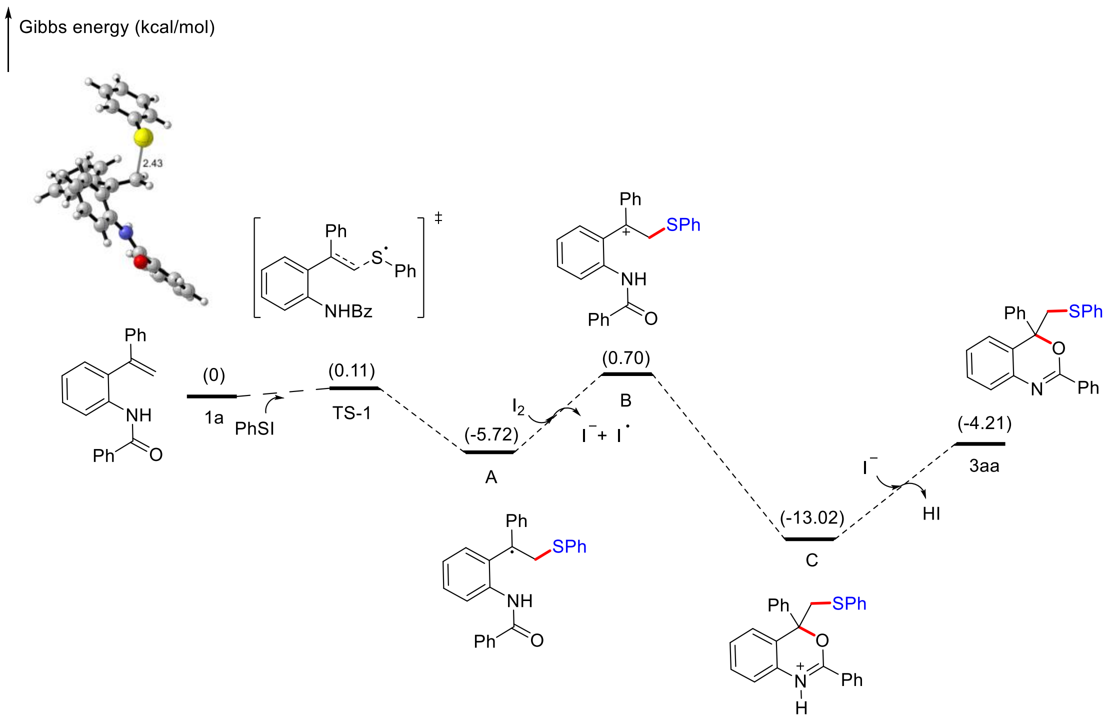

Fig. S1 Gibbs energy profiles for the radical pathway.

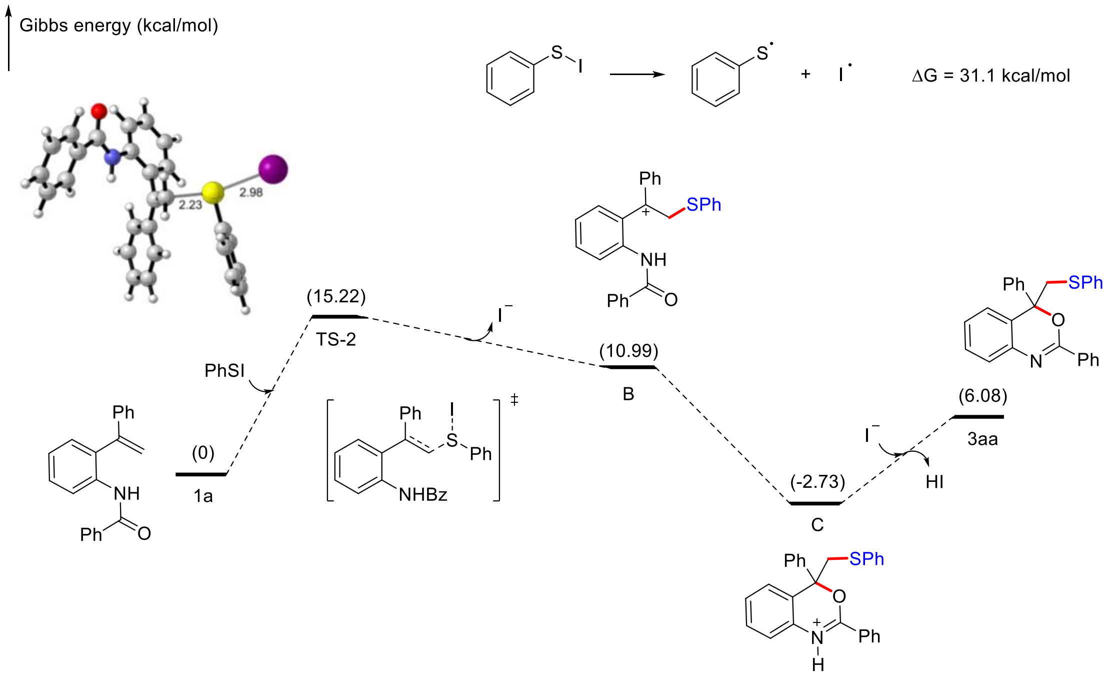

Fig. S2 Gibbs energy profiles for the reaction pathway involved PhSI.

5.2 Computational Methods and results

All the calculations were performed with the Gaussian 09 RevB.01 program suite.[2] The geometry optimization and energy calculations of the reactants, translation states, intermediates and products in this manuscript were fully optimized by the DFT/B3LYP method with the 6-311G (d, p) basis set (LanL2DZ for iodine) in solution phase using CPCM(Conductro-like Polarizable Continuum Model) in acetonitrile. Vibrational frequency analysis was calculated at the same level of theory to validate each structure as either a minimum or a transition state. For each transition state, the intrinsic reaction coordinate (IRC) analysis was conducted to ensure that it connects the right reactant and product. 3D structures were generated by CYLview, key bond distances shown in units of Å.

5.3 Gibbs free energy and Cartesian coordinate of optimized structures

Compound 1a

EE+TCG: -940.420155 Hartree

Cartesian coordinate of optimized structures:

C	-0.22681300	3.60022400	-0.66561100
C	-1.11108600	2.84962500	-1.43641900
C	-1.05485700	1.46000900	-1.40066300
C	-0.13514500	0.80537500	-0.57673500
C	0.77205200	1.55344800	0.20362800
C	0.70865400	2.95121700	0.13565300
C	1.76565500	0.89844900	1.10917500
C	1.77128500	1.19524200	2.41676000
C	2.76407900	-0.03602500	0.51563300
N	-0.09405700	-0.61502900	-0.53545700
C	3.23066000	-1.15299100	1.22837600
C	4.18989800	-1.99906500	0.68062500
C	4.69958000	-1.75312400	-0.59471900
C	4.23708600	-0.65633200	-1.31930600
C	3.27543700	0.19107500	-0.77241600

C	-1.12169800	-1.52682500	-0.53349100
C	-2.48246500	-1.09562600	-0.07246600
O	-0.90961900	-2.69285000	-0.85265200
C	-3.59909900	-1.67116800	-0.68899000
C	-4.88216900	-1.36300000	-0.24755400
C	-5.05903400	-0.50147700	0.83554800
C	-3.94888100	0.05249200	1.47225800
C	-2.66483400	-0.23560200	1.01657900
H	-0.25518900	4.68307100	-0.69586000
H	-1.83317900	3.34044700	-2.07837500
H	-1.72798000	0.87337600	-2.01309300
H	1.41127900	3.52990500	0.72365300
H	2.51410800	0.78460100	3.09057400
H	1.03425400	1.86459400	2.84403900
H	0.81030000	-1.04560000	-0.68985200
H	2.82219900	-1.36890300	2.20855700
H	4.53210700	-2.85875300	1.24565900
H	5.44352600	-2.41554000	-1.02203600
H	4.62525600	-0.45696200	-2.31170800
H	2.92821000	1.04517600	-1.34252500
H	-3.44977200	-2.35765700	-1.51340600
H	-5.74243600	-1.79977800	-0.74144100
H	-6.05787700	-0.26811800	1.18619300
H	-4.08209000	0.70840500	2.32474600
H	-1.80719300	0.19420200	1.51909000

Compound 2a

EE+TCG: -1259.707927 Hartree

Cartesian coordinate of optimized structures:

C	2.67963100	1.86465700	0.72908300

C	1.74219600	0.86992400	1.00244200
C	1.86184000	-0.38479700	0.39501500
C	2.92446300	-0.64182800	-0.47913200
C	3.85258400	0.35876800	-0.75312000
C	3.73205200	1.61135200	-0.14918000
H	2.58559200	2.83567400	1.20130300
H	0.92208500	1.06168500	1.68330000
H	3.01899700	-1.61814100	-0.93881200
H	4.67382600	0.15880300	-1.43162300
H	4.45928400	2.38654700	-0.36127400
S	0.69395500	-1.68081400	0.81248000
S	-0.69395500	-1.68081400	-0.81248000
C	-1.86184000	-0.38479700	-0.39501500
C	-2.92446300	-0.64182800	0.47913100
C	-1.74219600	0.86992400	-1.00244100
C	-3.85258500	0.35876900	0.75311900
H	-3.01899800	-1.61814100	0.93881200
C	-2.67963100	1.86465700	-0.72908300
H	-0.92208500	1.06168500	-1.68329900
C	-3.73205200	1.61135200	0.14917900
H	-4.67382700	0.15880300	1.43162300
H	-2.58559100	2.83567400	-1.20130300
H	-4.45928400	2.38654700	0.36127400

Compound I_2

EE+TCG: -22.796874 Hartree

Cartesian coordinate of optimized structures:

I	0.00000000	0.00000000	1.43308000
I	0.00000000	0.00000000	-1.43308000

Compound PhSI

EE+TCG: -641.252344 Hartree

Cartesian coordinate of optimized structures:

C	-3.21564700	-1.21085500	-0.40572700
C	-1.96991300	-1.21592200	0.21199900
C	-1.34391000	-0.00009900	0.52619300
C	-1.96982500	1.21582900	0.21226500
C	-3.21561500	1.21100200	-0.40536300
C	-3.83811200	0.00012900	-0.71353900
H	-3.70226000	-2.14898700	-0.64461900
H	-1.47794300	-2.14925900	0.45542700
H	-1.47781100	2.14908200	0.45592900
H	-3.70217100	2.14922100	-0.64402100
H	-4.80981600	0.00018900	-1.19344800
S	0.17234600	-0.00018900	1.44005800
I	1.99491700	0.00004300	-0.34009700

Compound PhS·

EE+TCG: -629.820917 Hartree

Cartesian coordinate of optimized structures:

C	-1.54333700	-1.20343200	0.00000400
C	-0.15032900	-1.21319000	0.00000300
C	0.55175700	-0.00000300	0.00001000
C	-0.15032600	1.21318900	0.00000200
C	-1.54333300	1.20343400	0.00000500
C	-2.24679600	0.00000200	0.00001200
H	-2.07704400	-2.14719100	0.00000100
H	0.38352700	-2.15605800	-0.00000500
H	0.38353300	2.15605400	-0.00000600
H	-2.07703900	2.14719400	0.00000300

H	-3.33017400	0.00000400	0.00001300
S	2.32571200	0.00000000	-0.00001400

Compound I·

EE+TCG: -11.381837 Hartree

Cartesian coordinate of optimized structures:

I	0.00000000	0.00000000	0.00000000

Compound I^-

EE+TCG: -11.588041 Hartree

Cartesian coordinate of optimized structures:

I	0.00000000	0.00000000	0.00000000

Compound HI

EE+TCG: -12.004155 Hartree

Cartesian coordinate of optimized structures:

I	0.00000000	0.00000000	0.02989600
H	0.00000000	0.00000000	-1.58449200

Compound TS-1

EE+TCG: -1570.240889 Hartree

Cartesian coordinate of optimized structures:

C	0.05132700	4.17279300	-0.21839600
C	1.42425100	4.27345000	-0.43203600
C	2.22996000	3.14007300	-0.41736900
C	1.66574600	1.87487700	-0.19906800
C	0.26915500	1.75685900	0.00383300
C	-0.50982900	2.92085900	0.00596200
C	-0.40123100	0.43500400	0.18501900
C	-0.38389600	-0.46195200	-0.87307900
N	2.45110300	0.70711300	-0.11494400

C	3.75800500	0.51409100	-0.49179400
O	4.44781000	1.37511100	-1.02509100
C	4.30038000	-0.86150900	-0.22121100
C	5.37121000	-1.29614500	-1.01183300
C	5.93187900	-2.55250400	-0.81075500
C	5.44056200	-3.38379700	0.19652500
C	4.38827500	-2.95120800	1.00169300
C	3.81798700	-1.69710800	0.79460000
S	-2.18272600	0.09293300	-2.40948800
C	-1.10278900	0.15657800	1.44281400
C	-2.03406500	-0.89858000	1.56141300
C	-2.65974000	-1.16798400	2.77099600
C	-2.37443400	-0.39990500	3.90194500
C	-1.45530700	0.64481200	3.80737100
C	-0.83145900	0.92260100	2.59699100
C	-3.63317900	-0.61126100	-1.70019500
C	-3.89827700	-1.98997600	-1.81864700
C	-5.05860600	-2.53390600	-1.27964900
C	-5.97809200	-1.71570600	-0.61897200
C	-5.72907800	-0.34737500	-0.49837000
C	-4.56659400	0.20201800	-1.02815600
H	-0.57578600	5.05620700	-0.22863100
H	1.87980100	5.24173200	-0.60542600
H	3.29346900	3.21934300	-0.57561800
H	-1.57725700	2.82822900	0.16789400
H	-0.69959300	-1.48768600	-0.73952300
H	0.29832100	-0.29127300	-1.69510400
H	1.95173300	-0.11530000	0.19082000
H	5.75088800	-0.63676700	-1.78207600
H	6.75257000	-2.88331300	-1.43658700

H	5.88014700	-4.36142200	0.35732100
H	4.01456900	-3.58564700	1.79675300
H	3.02153800	-1.37008700	1.45324500
H	-2.28649600	-1.49951200	0.69812600
H	-3.37733900	-1.97802400	2.83233100
H	-2.86385600	-0.61409500	4.84491600
H	-1.22166400	1.24405100	4.67990600
H	-0.11536700	1.73225700	2.54210600
H	-3.18955600	-2.62267300	-2.33942900
H	-5.25062400	-3.59625600	-1.37878700
H	-6.88336500	-2.14216400	-0.20265900
H	-6.44097500	0.29119400	0.01216900
H	-4.37073500	1.26300400	-0.93240800

Compound TS-2

EE+TCG: -1581.648249 Hartree

Cartesian coordinate of optimized structures:

C	-0.40859200	-1.10437500	3.89623700
C	-1.60426100	-1.81923500	3.85245000
C	-2.48118900	-1.67253400	2.78445600
C	-2.16594400	-0.81410800	1.72194900
C	-0.94463300	-0.09739500	1.74708900
C	-0.09557900	-0.24233400	2.85424500
C	-0.51797800	0.76643100	0.61163700
C	-0.31966800	0.14588000	-0.63050300
N	-3.06518200	-0.58757100	0.66076500
C	-4.09745800	-1.39282000	0.23026000
O	-4.34782700	-2.48650100	0.71806800
C	-4.89837400	-0.84073800	-0.91395700
C	-5.58035300	-1.75890000	-1.72191000

C	-6.34886500	-1.31896400	-2.79373200
C	-6.45975800	0.04625800	-3.06020000
C	-5.80047900	0.96820500	-2.24899600
C	-5.02101300	0.52906400	-1.18140300
S	1.75120200	-0.63802000	-0.36159100
C	-0.31844700	2.19565500	0.82660300
C	0.20302300	3.03839700	-0.18320300
C	0.32993400	4.40277700	0.02282900
C	-0.06353100	4.97202100	1.23632400
C	-0.59117900	4.16309500	2.24226700
C	-0.71789900	2.79550000	2.04326300
C	2.67924800	0.78419600	-0.89449400
C	2.75096200	1.11129400	-2.25673800
C	3.49244900	2.21405200	-2.66628900
C	4.17567100	2.98765600	-1.72528200
C	4.11218700	2.66085800	-0.37116400
C	3.36301700	1.56497100	0.04775900
H	0.27355400	-1.21869600	4.72966100
H	-1.86628200	-2.49158600	4.66111300
H	-3.41095600	-2.21799700	2.76028800
H	0.83711700	0.30767900	2.86935100
H	-0.22095000	0.73491600	-1.53194100
H	-0.74702000	-0.83656900	-0.77912900
H	-2.87907100	0.22727600	0.09646400
H	-5.49449300	-2.81460800	-1.49780200
H	-6.86293500	-2.03865100	-3.42006200
H	-7.06258300	0.39020800	-3.89270200
H	-5.89736200	2.03018400	-2.44123600
H	-4.54317000	1.26617400	-0.54626800
H	0.51730000	2.62703600	-1.13159200

H	0.73584200	5.02746100	-0.76353200
H	0.03406300	6.03998400	1.39199700
H	-0.91084500	4.60016100	3.18054300
H	-1.14743500	2.18491700	2.82506300
H	2.23383700	0.49840900	-2.98498000
H	3.54563000	2.46573200	-3.71898200
H	4.75799300	3.84242200	-2.04905700
H	4.64269000	3.26037300	0.35914200
H	3.31147800	1.30456300	1.09745100
I	4.00651900	-2.57897200	-0.46443800

Compound A

EE+TCG: -1570.250191 Hartree

Cartesian coordinate of optimized structures:

C	-0.16270014	4.19698528	0.13393852
C	1.20483067	4.34195333	-0.08957369
C	2.03166963	3.22565600	-0.16266677
C	1.49080660	1.94076424	-0.02647147
C	0.09899835	1.77368499	0.17104557
C	-0.70051139	2.92099309	0.26745048
C	-0.52297591	0.42309477	0.22859784
C	-0.53005323	-0.35341497	-1.04469167
N	2.30014230	0.78392361	-0.00927611
C	3.56535750	0.61766019	-0.51838310
O	4.16217872	1.48684434	-1.14240571
C	4.17999178	-0.73176593	-0.27364201
C	5.17553116	-1.15748173	-1.16149015
C	5.79478097	-2.39031425	-0.98757687
C	5.43918264	-3.20561596	0.08772010
C	4.46418223	-2.78048780	0.98835353

C	3.83529184	-1.55040791	0.80959575
S	-1.81595615	0.22208182	-2.30537977
C	-1.14687562	-0.05327540	1.42903916
C	-1.90113990	-1.25978477	1.47196561
C	-2.46528755	-1.71636240	2.65255656
C	-2.30716649	-1.00125188	3.84359347
C	-1.56782216	0.18556345	3.83250650
C	-0.99950171	0.65183823	2.65827320
C	-3.34480022	-0.50590124	-1.71110241
C	-3.60899749	-1.86587035	-1.91669818
C	-4.81344425	-2.41585627	-1.48448572
C	-5.76891620	-1.61115684	-0.86262645
C	-5.51594550	-0.25364099	-0.67292722
C	-4.30682516	0.29949926	-1.09239322
H	-0.80599672	5.06671547	0.19676043
H	1.63869765	5.32949418	-0.19762918
H	3.09279601	3.33784565	-0.32167550
H	-1.76486827	2.79728722	0.42899845
H	-0.66526678	-1.42437490	-0.91081040
H	0.39241210	-0.19849751	-1.60550239
H	1.85584166	-0.03690912	0.37665189
H	5.45043770	-0.51014918	-1.98467270
H	6.55580254	-2.71518182	-1.68755106
H	5.92487529	-4.16459985	0.22673078
H	4.19724597	-3.40137552	1.83544770
H	3.10310982	-1.22674550	1.54030632
H	-2.05891273	-1.83182694	0.56822941
H	-3.03872808	-2.63659475	2.64654500
H	-2.75045945	-1.36326209	4.76373936
H	-1.43082196	0.74631959	4.75044152

H	-0.41978306	1.56551358	2.67649588
H	-2.87445309	-2.48734482	-2.41532407
H	-5.00980250	-3.47009886	-1.64324204
H	-6.70807726	-2.04014580	-0.53281533
H	-6.25663172	0.37648180	-0.19389137
H	-4.10632641	1.35298624	-0.94043144

Compound B

EE+TCG: -1570.066954 Hartree

Cartesian coordinate of optimized structures:

C	0.25932000	4.57904500	0.31655800
C	1.57895300	4.41591600	0.76091900
C	2.19461800	3.17766200	0.73168900
C	1.51026800	2.03365700	0.28963500
C	0.12014500	2.16582500	-0.09753300
C	-0.43670300	3.47864800	-0.11757600
C	-0.71985500	1.07498800	-0.49816300
C	-1.76267900	1.30008300	-1.53292200
N	2.19307900	0.83321100	0.13556700
C	3.56947800	0.65079500	-0.03217800
O	4.36767200	1.56976800	-0.05880700
C	3.99500900	-0.77324900	-0.20536000
C	5.16512600	-1.00987900	-0.93839900
C	5.62738200	-2.30788600	-1.11997500
C	4.93826400	-3.38098500	-0.55295100
C	3.78612600	-3.15075400	0.19670600
C	3.31168300	-1.85270500	0.36816500
S	-3.38025600	1.64623500	-0.64473100
C	-0.64830200	-0.23358500	0.12074800
C	-0.93524600	-1.41765300	-0.60034400

C	-0.91217800	-2.64911100	0.03508200
C	-0.64879000	-2.72543600	1.40369300
C	-0.38699400	-1.56525100	2.13956100
C	-0.37250900	-0.33361300	1.50847300
C	-4.06957800	0.02339400	-0.35660200
C	-4.49931200	-0.77698600	-1.42378100
C	-5.10378400	-2.00250200	-1.16499100
C	-5.30232000	-2.42352400	0.15153900
C	-4.89093900	-1.61861600	1.21245700
C	-4.26867600	-0.39730000	0.96324700
H	-0.21346300	5.55216700	0.33423100
H	2.13636900	5.27016900	1.12612600
H	3.22544600	3.08272000	1.02777000
H	-1.47060000	3.60150900	-0.40566400
H	-1.91785900	0.43454800	-2.16984200
H	-1.56977700	2.16923300	-2.15413000
H	1.63495200	0.01948300	-0.07282200
H	5.69725800	-0.16838600	-1.36362800
H	6.52543800	-2.48402800	-1.70013700
H	5.30223900	-4.39264200	-0.68955200
H	3.25914700	-3.97922200	0.65463900
H	2.43436800	-1.69175900	0.98312200
H	-1.11768700	-1.37761000	-1.66550300
H	-1.10320400	-3.55040600	-0.53321100
H	-0.65055300	-3.68849800	1.89983700
H	-0.20526300	-1.62643500	3.20515400
H	-0.19981600	0.56684100	2.08389900
H	-4.36911300	-0.44004000	-2.44532300
H	-5.43243200	-2.62308900	-1.99019800
H	-5.78095400	-3.37568300	0.34795600

H	-5.04671400	-1.94224000	2.23472600
H	-3.93765600	0.22711900	1.78356300

Compound C

EE+TCG: -1570.088824 Hartree

Cartesian coordinate of optimized structures:

C	3.68044800	-1.61811300	-2.35750300
C	3.56891700	-0.54477700	-3.23946800
C	2.66508400	0.47904300	-2.97983200
C	1.89107400	0.41547000	-1.82385900
C	1.99718700	-0.64152000	-0.91915400
C	2.89756700	-1.66637300	-1.20566100
N	0.92999000	1.42011900	-1.56080400
C	-0.05870900	1.25459500	-0.70281600
O	-0.06607700	0.21911100	0.08710800
C	1.19133100	-0.57038800	0.36155600
C	0.66437400	-1.93143400	0.84482700
C	-1.18506600	2.17603200	-0.61142900
C	-1.50466800	3.04010000	-1.67314400
C	-2.57725300	3.91181800	-1.55433300
C	-3.34055100	3.92652500	-0.38561700
C	-3.03498300	3.06262600	0.66602700
C	-1.96468500	2.18546600	0.55713600
C	1.91123000	0.15543600	1.50349300
C	1.19563900	0.48522200	2.66412300
C	1.82741500	1.11879300	3.72849300
C	3.18628700	1.42540600	3.65505500
C	3.90400700	1.09941400	2.50829600
C	3.27096100	0.47137600	1.43583100
S	-0.29544900	-2.91544000	-0.38086400

C	-2.00028600	-2.48630400	0.00656600
C	-2.77387600	-1.83021000	-0.95467300
C	-4.11815900	-1.56026100	-0.70011700
C	-4.68964600	-1.92961900	0.51608000
C	-3.91669200	-2.58306500	1.47624900
C	-2.57890100	-2.87475300	1.21965500
H	4.38172600	-2.41774300	-2.56025400
H	4.17927200	-0.50567700	-4.13287600
H	2.55280700	1.30992200	-3.66594000
H	3.01378800	-2.49770400	-0.52267700
H	0.94599500	2.26012800	-2.12558900
H	1.51594800	-2.54236000	1.14167700
H	0.04643400	-1.79016500	1.72903200
H	-0.94771500	3.01823000	-2.60178200
H	-2.82532900	4.57168000	-2.37598200
H	-4.17763400	4.60877400	-0.29815600
H	-3.62985400	3.07408800	1.57064900
H	-1.72110300	1.51749800	1.37113000
H	0.13942900	0.25776800	2.73892600
H	1.25835300	1.37111100	4.61548800
H	3.67923700	1.91622800	4.48604600
H	4.95936800	1.33551300	2.43986200
H	3.84645300	0.23004900	0.55224900
H	-2.32496900	-1.53054400	-1.89367900
H	-4.71386200	-1.05322300	-1.45045000
H	-5.73300400	-1.71338900	0.71425400
H	-4.35830700	-2.88118900	2.42020800
H	-1.98943700	-3.40870400	1.95578200

Compound 3aa

EE+TCG: -1569.658670 Hartree

Cartesian coordinate of optimized structures:

C	0.83452000	-3.20279700	2.86020600
C	1.50309200	-2.34074800	3.73145700
C	1.82096400	-1.05185300	3.32316900
C	1.47215800	-0.60363400	2.04092800
C	0.80089000	-1.47213600	1.16719400
C	0.49107300	-2.76696700	1.58254900
N	1.81174800	0.70690400	1.68069400
C	1.55905600	1.09139900	0.48519300
O	0.97970500	0.34625600	-0.48436200
C	0.39831500	-0.97359100	-0.21765900
C	-1.12551000	-0.80392800	-0.34839300
C	1.92034800	2.44736200	0.01313200
C	1.60365500	2.87313300	-1.28429100
C	1.95220500	4.15377500	-1.70578400
C	2.61742700	5.02088600	-0.84084700
C	2.93549700	4.60201500	0.45274900
C	2.59118700	3.32482900	0.87788900
C	0.97244400	-1.88574600	-1.31424300
C	0.18244300	-2.71245200	-2.11650500
C	0.76710200	-3.53281800	-3.08390600
C	2.14625700	-3.53457200	-3.26401700
C	2.94314700	-2.71024800	-2.46806800
C	2.36128100	-1.89670500	-1.50252200
S	-1.82334800	0.31397000	0.95102800
C	-3.48432600	0.53200000	0.29237600
C	-3.75241700	1.56950400	-0.60670500
C	-5.04662800	1.75533700	-1.08915600

C	-6.07728100	0.91309000	-0.67290600
C	-5.81244100	-0.11766000	0.22785500
C	-4.51934700	-0.30928300	0.71238200
H	0.58482500	-4.21010000	3.17175700
H	1.77615200	-2.67556400	4.72554300
H	2.34124700	-0.36555800	3.98094700
H	-0.01713000	-3.44470100	0.90555800
H	-1.35615600	-0.37327000	-1.32313000
H	-1.61708100	-1.77117600	-0.25371400
H	1.08738100	2.20163700	-1.95649200
H	1.70283300	4.47412200	-2.71078600
H	2.88735000	6.01740500	-1.17132700
H	3.45337300	5.27252400	1.12878400
H	2.83376600	2.98889700	1.87736300
H	-0.89367100	-2.73215700	-2.00572000
H	0.13617500	-4.16677600	-3.69626100
H	2.59878600	-4.17054100	-4.01607400
H	4.01919800	-2.70197200	-2.59925700
H	2.99269500	-1.26312400	-0.89139100
H	-2.95145900	2.22673700	-0.92327000
H	-5.24920100	2.56066600	-1.78591000
H	-7.08372100	1.06165500	-1.04691800
H	-6.61130100	-0.77256300	0.55644500
H	-4.31102000	-1.10718600	1.41498700

6. Reference

1. Wang YM, Wu J, Hoong C, et al. *J Am Chem Soc*, 2012, 134: 12928-12931;

2. M. J. Frisch, G. W. Trucks, H. B. Schlegel, G. E. Scuseria, M. A. Robb, J. R. Cheeseman, G. Scalmani, V. Barone, B. Mennucci, G. A. Petersson, H. Nakatsuji, M. Caricato, X. Li, H. P. Hratchian, A. F.

Izmaylov, J. Bloino, G. Zheng, J. L. Sonnenberg, M. Hada, M. Ehara, K. Toyota, R. Fukuda, J. Hasegawa, M. Ishida, T. Nakajima, Y. Honda, O. Kitao, H. Nakai, T. Vreven, J. A. Montgomery, Jr., J. E. Peralta, F. Ogliaro, M. Bearpark, J. J. Heyd, E. Brothers, K. N. Kudin, V. N. Staroverov, T. Keith, R. Kobayashi, J. Normand, K. Raghavachari, A. Rendell, J. C. Burant, S. S. Iyengar, J. Tomasi, M. Cossi, N. Rega, J. M. Millam, M. Klene, J. E. Knox, J. B. Cross, V. Bakken, C. Adamo, J. Jaramillo, R. Gomperts, R. E. Stratmann, O. Yazyev, A. J. Austin, R. Cammi, C. Pomelli, J. W. Ochterski, R. L. Martin, K. Morokuma, V. G. Zakrzewski, G. A. Voth, P. Salvador, J. J. Dannenberg, S. Dapprich, A. D. Daniels, O. Farkas, J. B. Foresman, J. V. Ortiz, J. Cioslowski, and D. J. Fox, Gaussian, Inc., Wallingford CT, 2010.

3. Legault, C. Y. CYLview, 1.0b; Université de Sherbrooke, 2009; http://www.cylview.org.

7. NMR Spectra of Product

2,4-diphenyl-4-((phenylthio)methyl)-4*H*-benzo[*d*][1,3]oxazine (3aa)

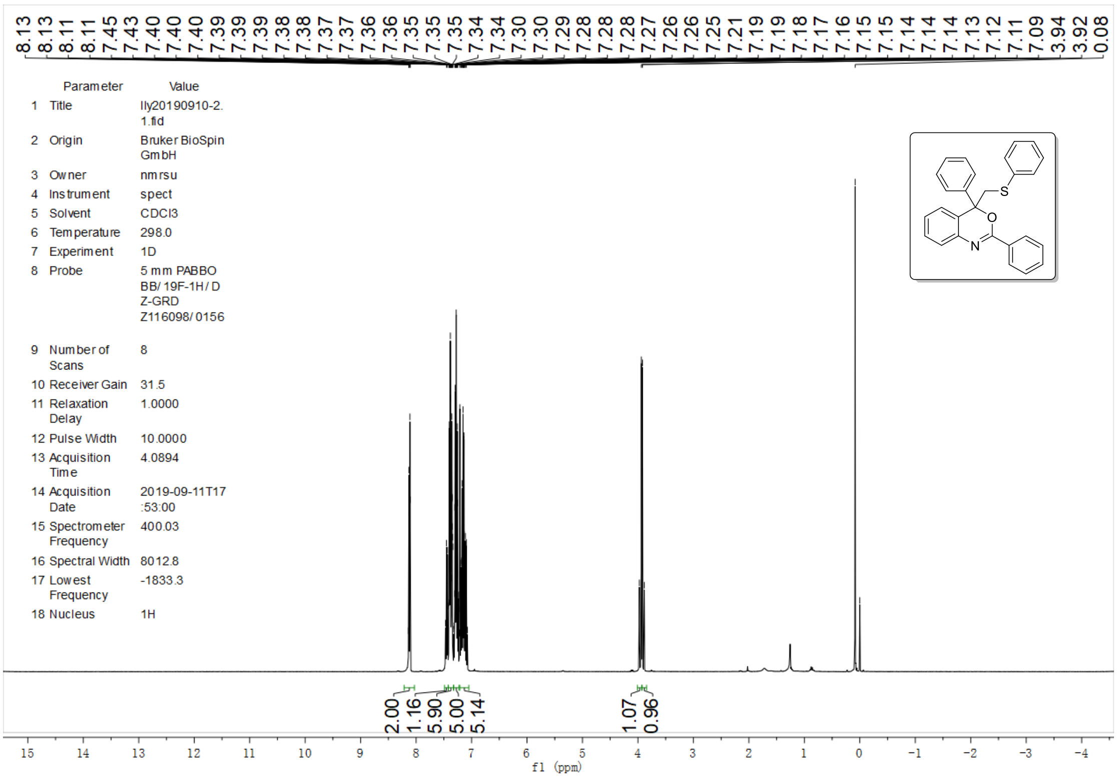

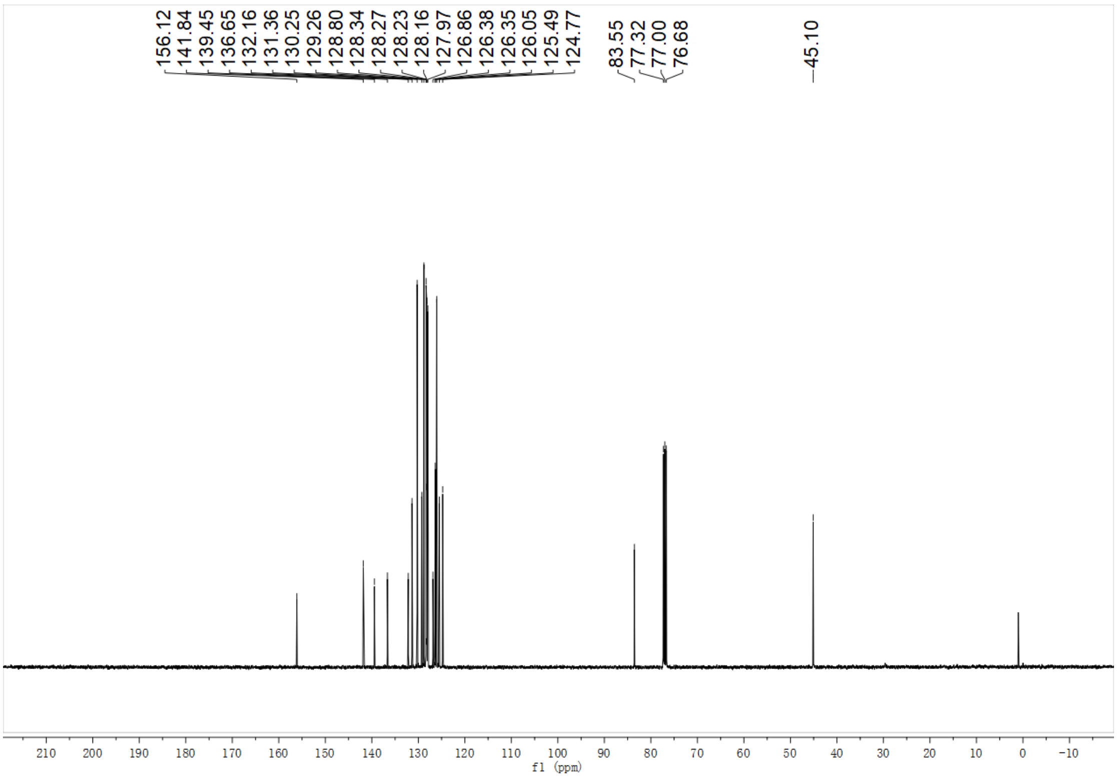

6-fluoro-2,4-diphenyl-4-((phenylthio)methyl)-4*H*-benzo[*d*][1,3]oxazine (3ba)

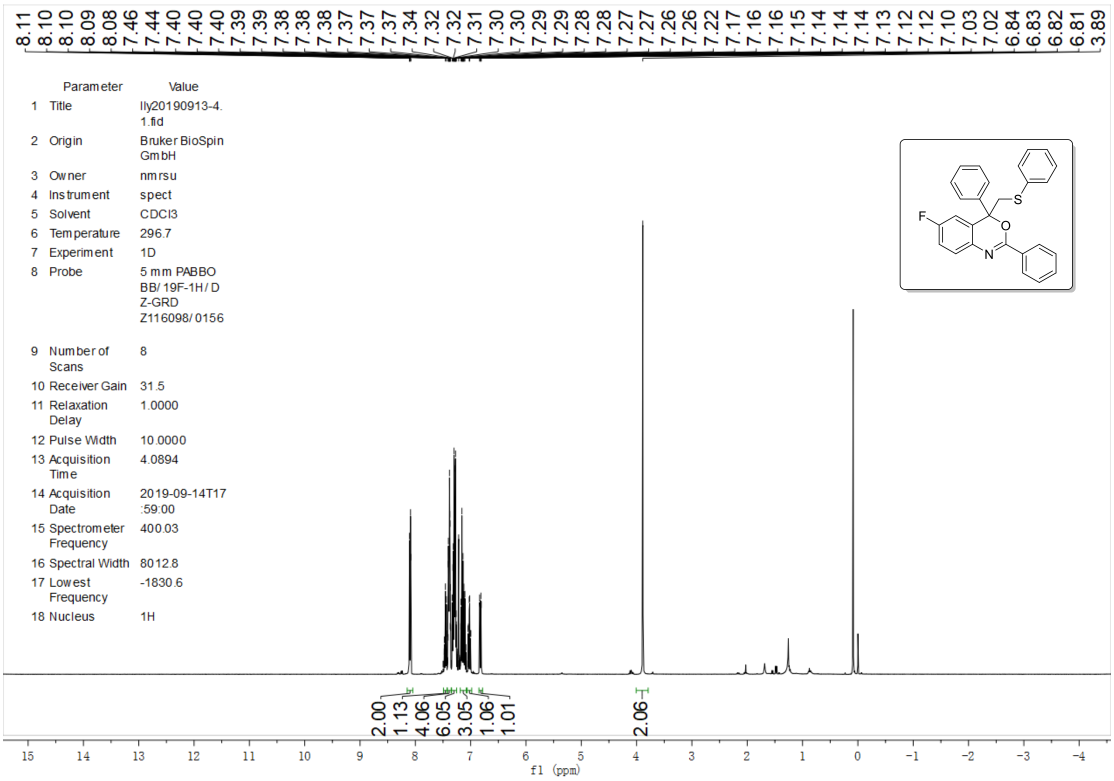

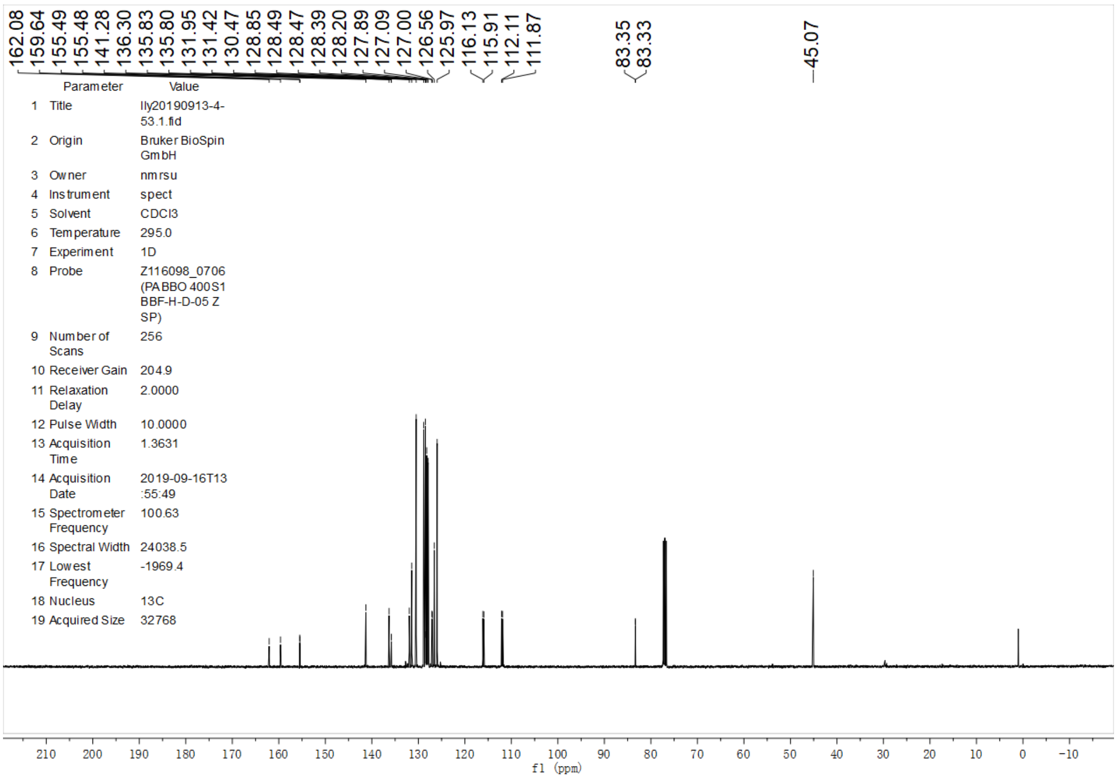

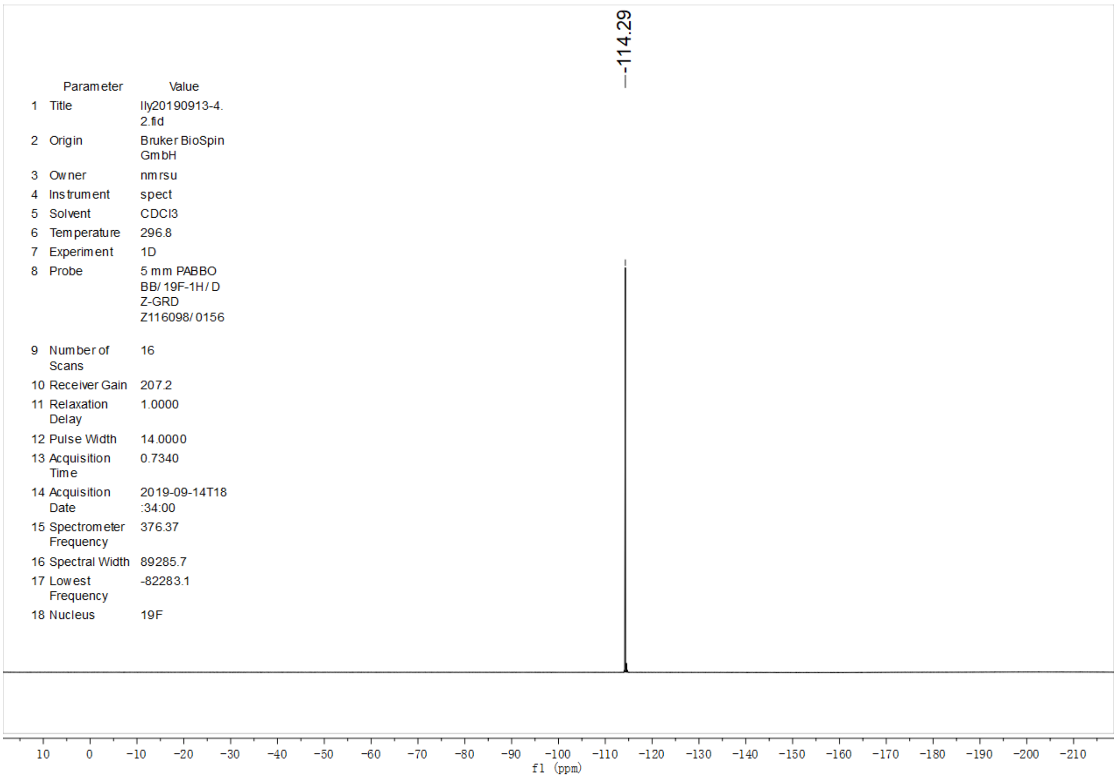
-114.29
Parameter Value
1 Title lly20190913-4.2.fid
2 Origin Bruker BioSpin GmbH
3 Owner nmrsu
4 Instrument spect
5 Solvent CDCl3
6 Temperature 296.8
7 Experiment 1D
8 Probe 5 mm PABBO BB/ 19F-1H/ D Z-GRD Z116098/ 0156
9 Number of Scans 16
10 Receiver Gain 207.2
11 Relaxation Delay 1.0000
12 Pulse Width 14.0000
13 Acquisition Time 0.7340
14 Acquisition Date 2019-09-14T18:34:00
15 Spectrometer Frequency 376.37
16 Spectral Width 89285.7
17 Lowest Frequency -82283.1
18 Nucleus 19F
10 0 -10 -20 -30 -40 -50 -60 -70 -80 -90 -100 -110 -120 -130 -140 -150 -160 -170 -180 -190 -200 -210
f1 (ppm)

6-chloro-2,4-diphenyl-4-((phenylthio)methyl)-4*H*-benzo[*d*][1,3]oxazine (3ca)

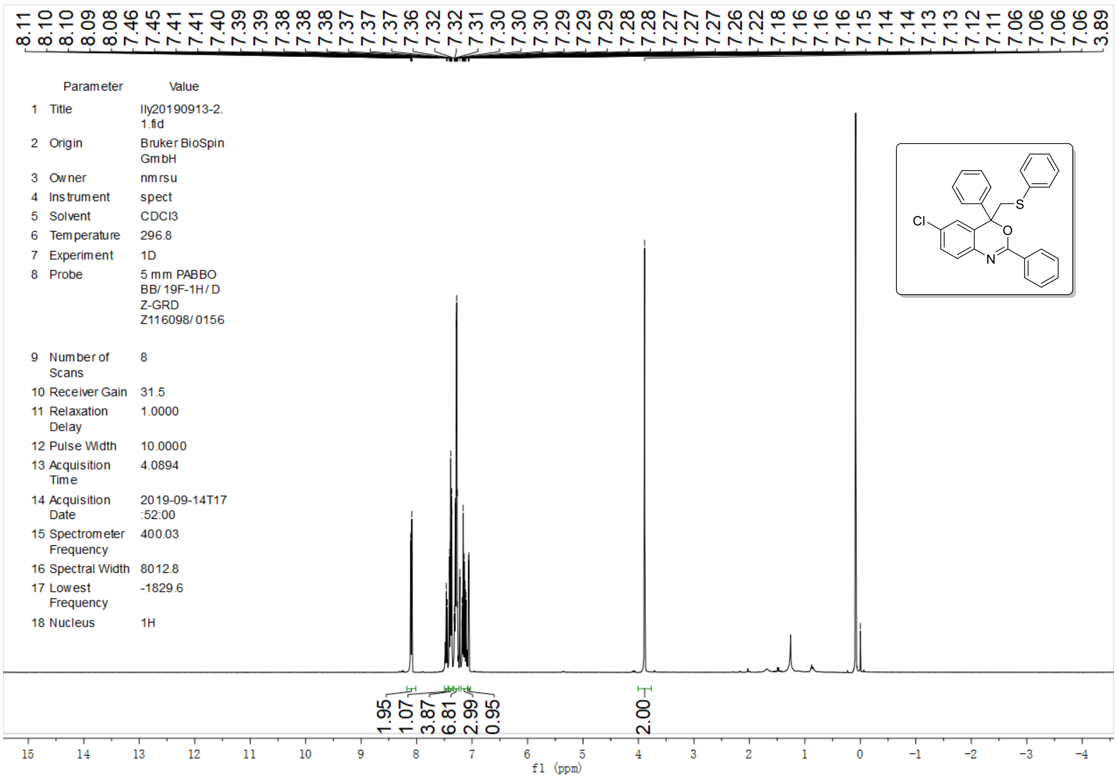

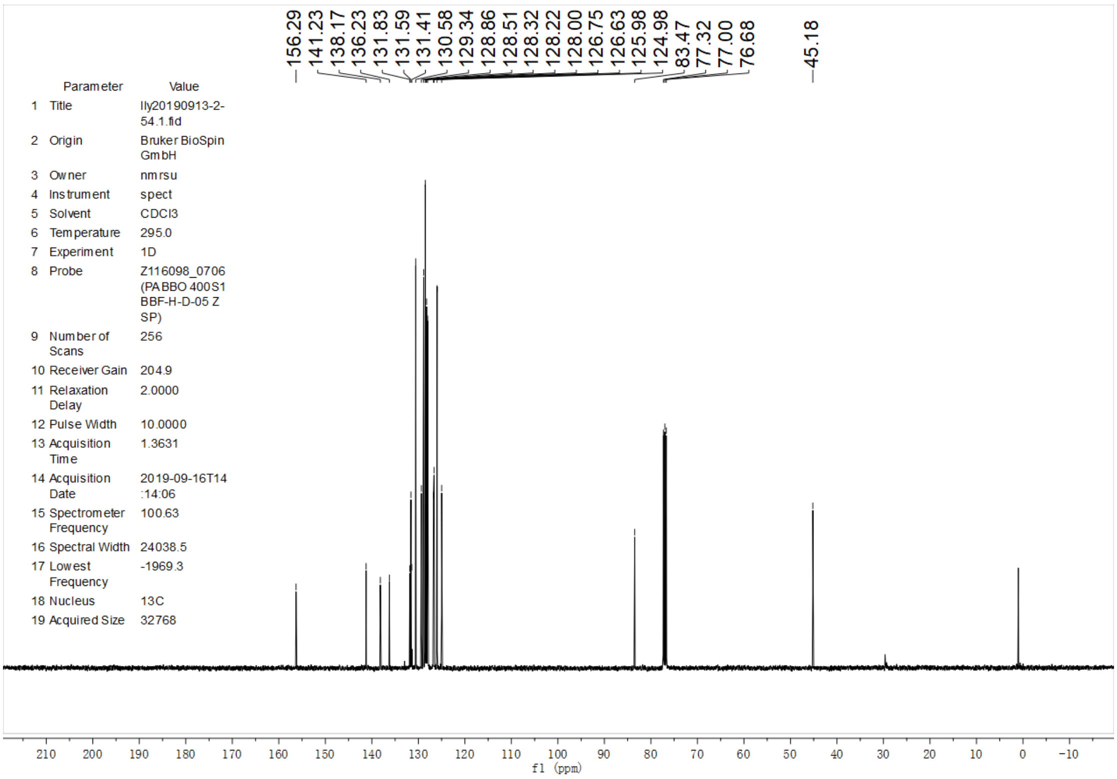

6-bromo-2,4-diphenyl-4-((phenylthio)methyl)-4*H*-benzo[*d*][1,3]oxazine (3da)

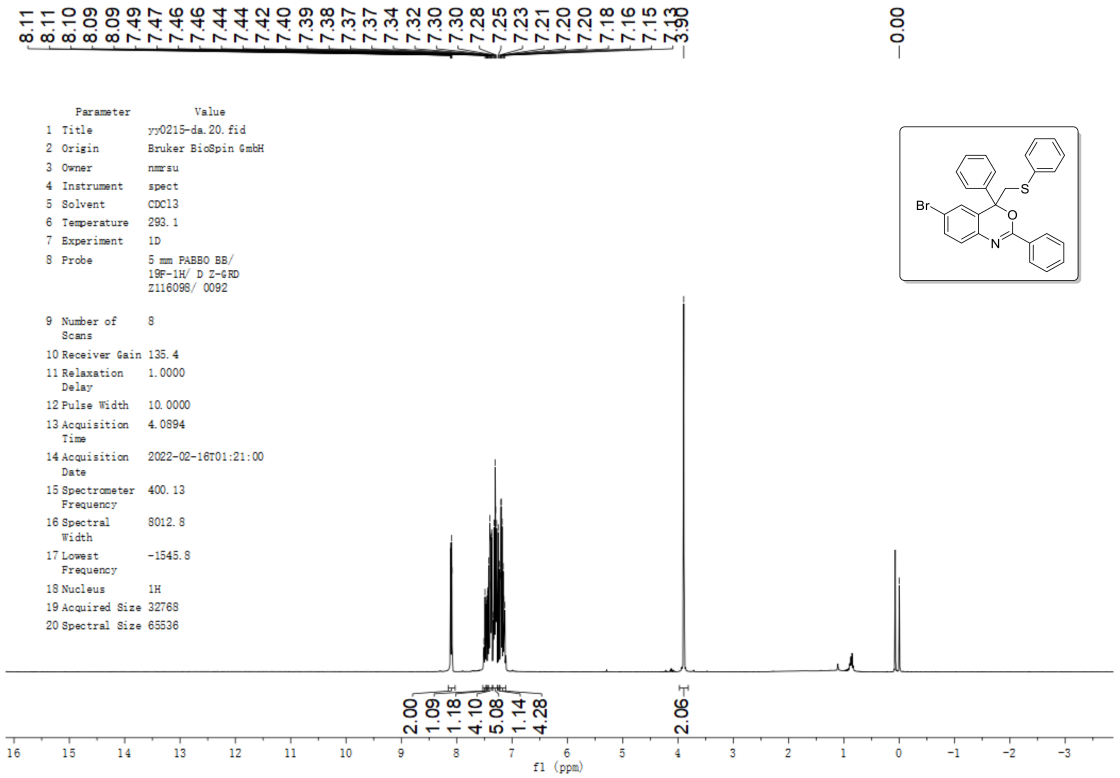

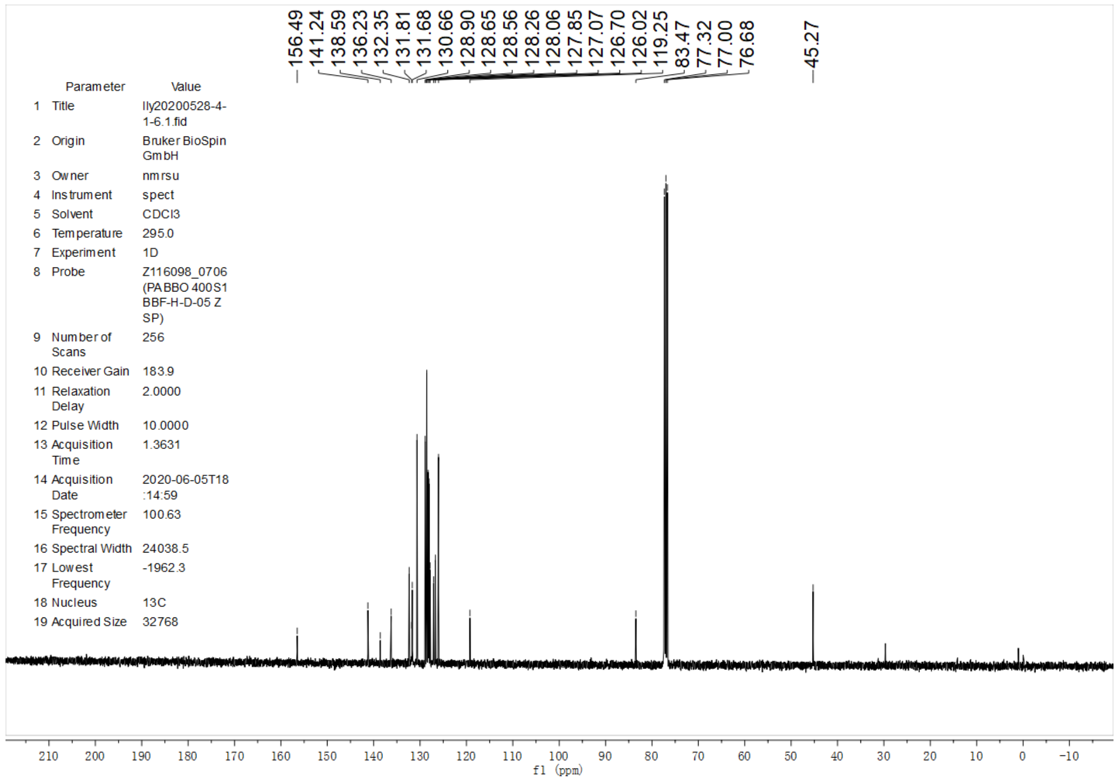

6-nitro-2,4-diphenyl-4-((phenylthio)methyl)-4*H*-benzo[*d*][1,3]oxazine (3ea)

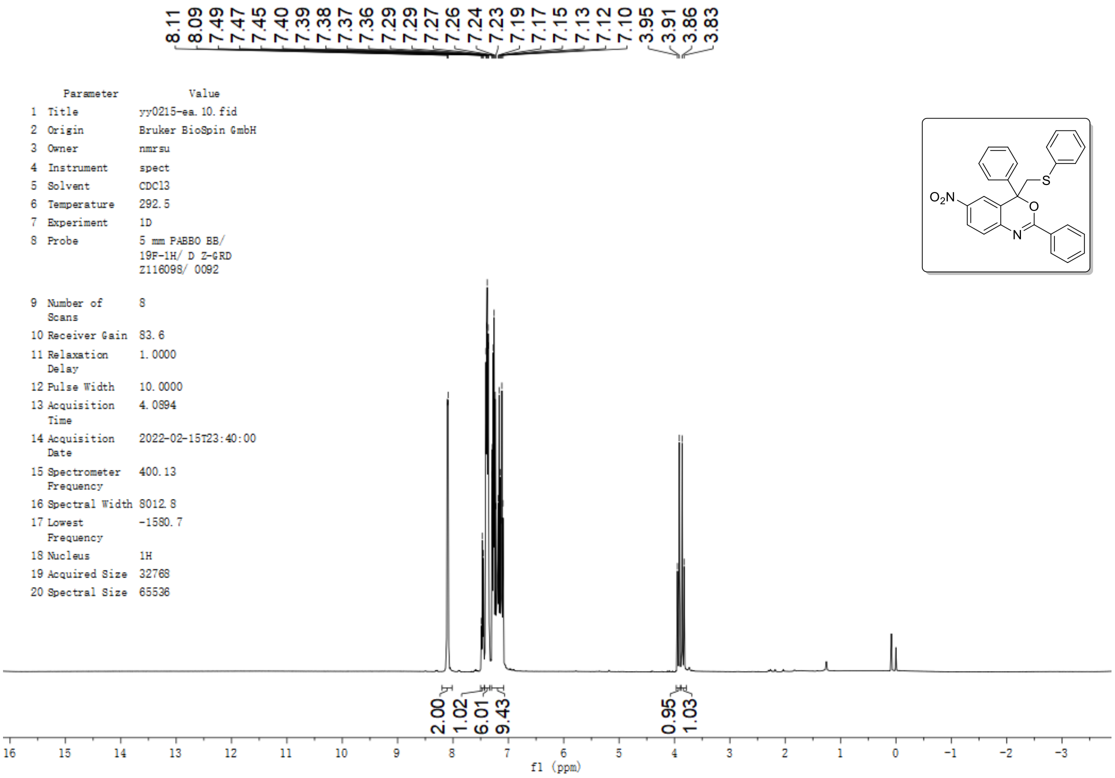

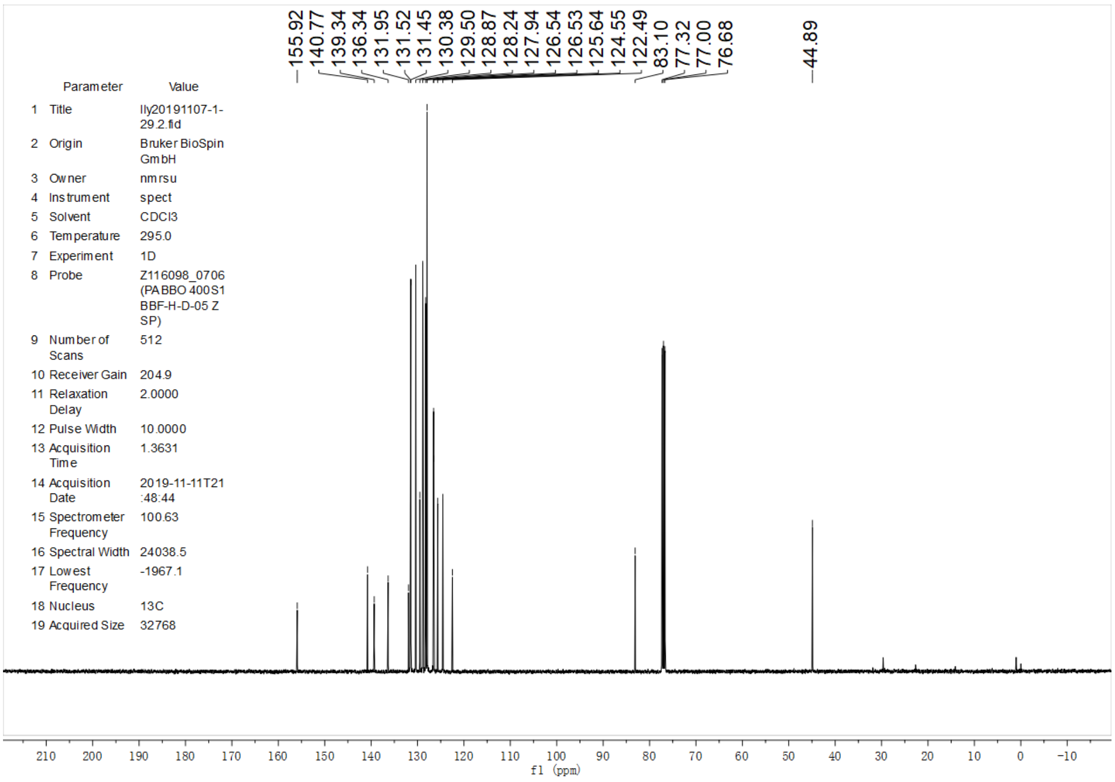

6-methoxy-2,4-diphenyl-4-((phenylthio)methyl)-4*H*-benzo[*d*][1,3]oxazine (3fa)

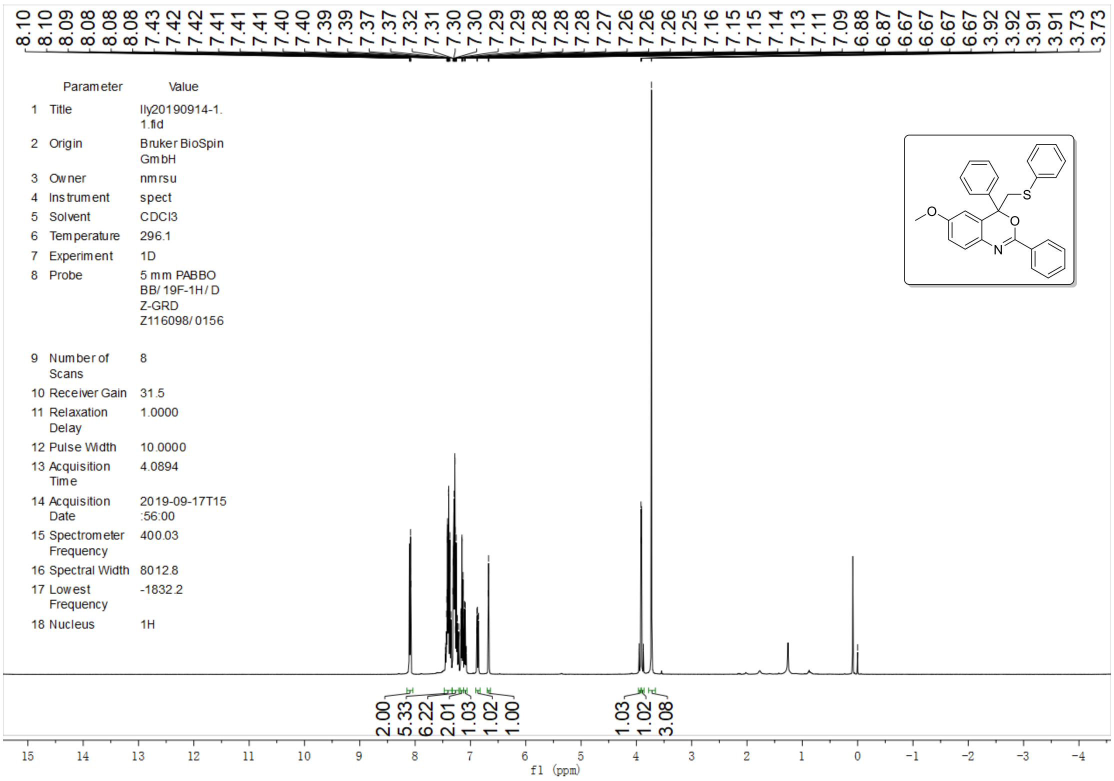

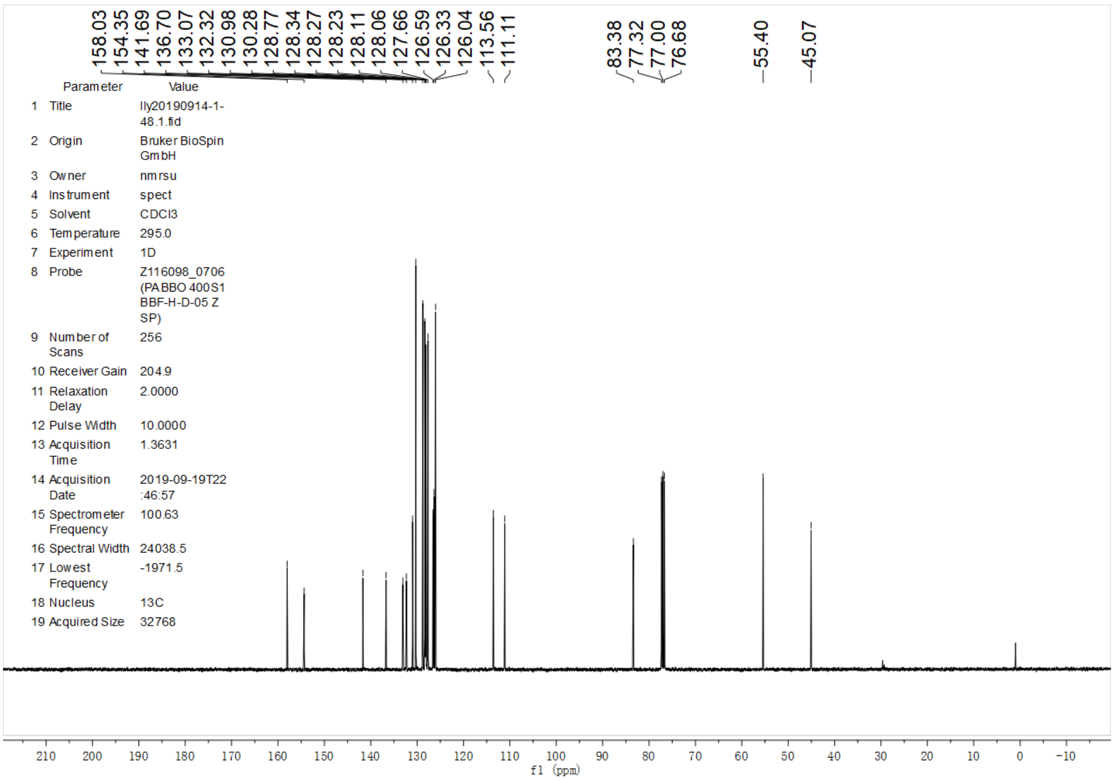

6-methyl-2,4-diphenyl-4-((phenylthio)methyl)-4*H*-benzo[*d*][1,3]oxazine (3ga)

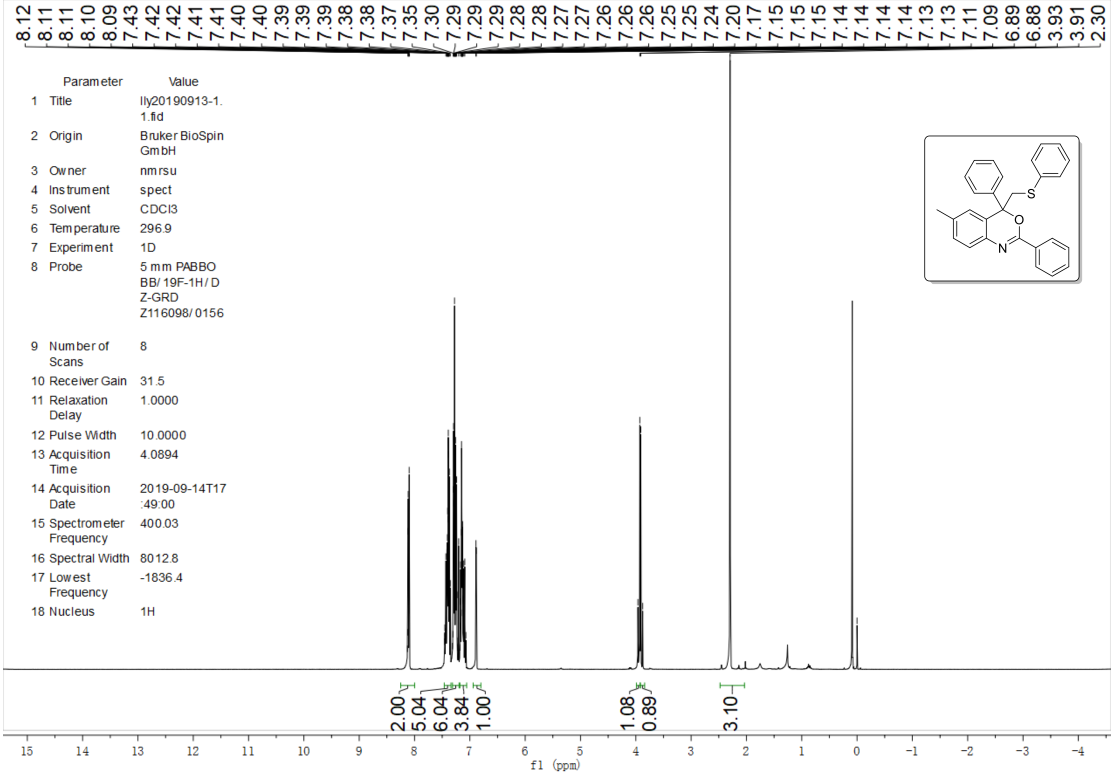

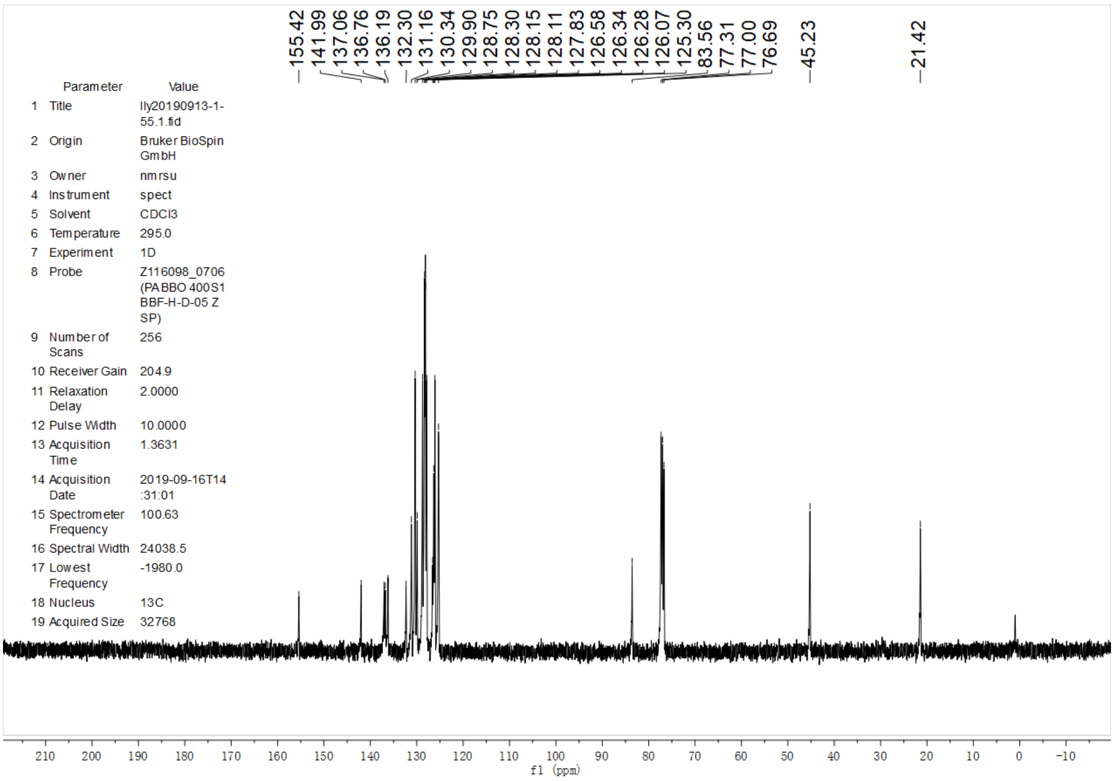

6-butyl-2,4-diphenyl-4-((phenylthio)methyl)-4*H*-benzo[*d*][1,3]oxazine (3ha)

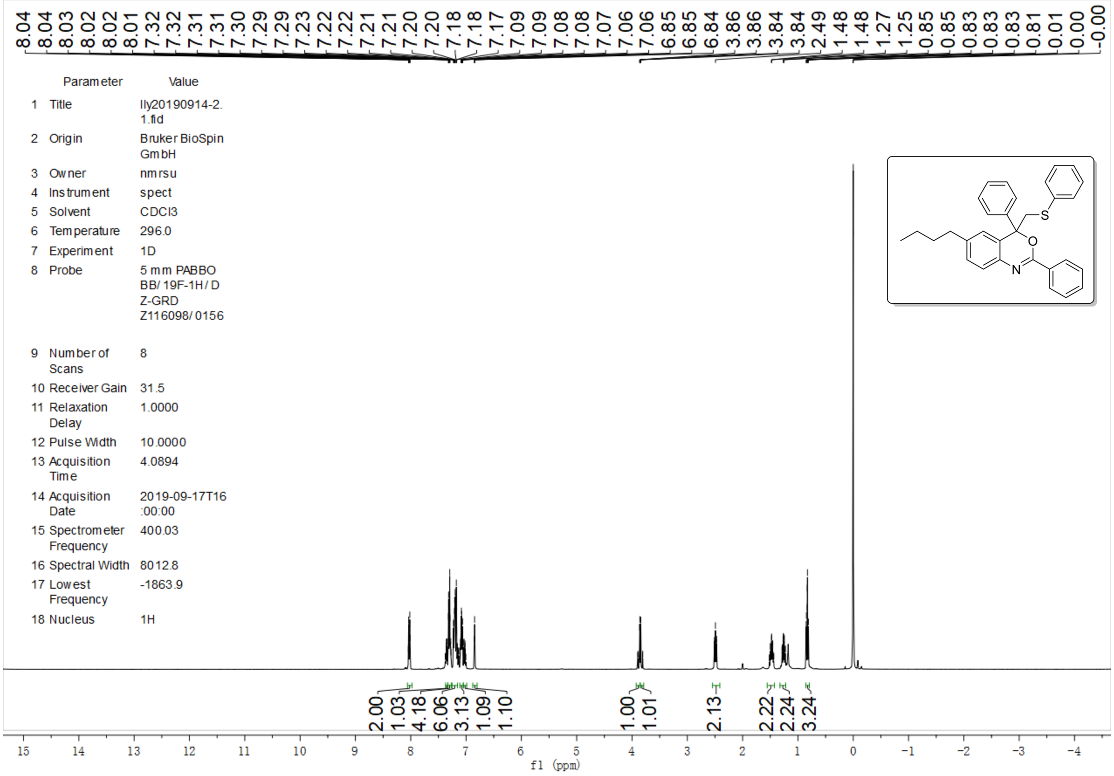

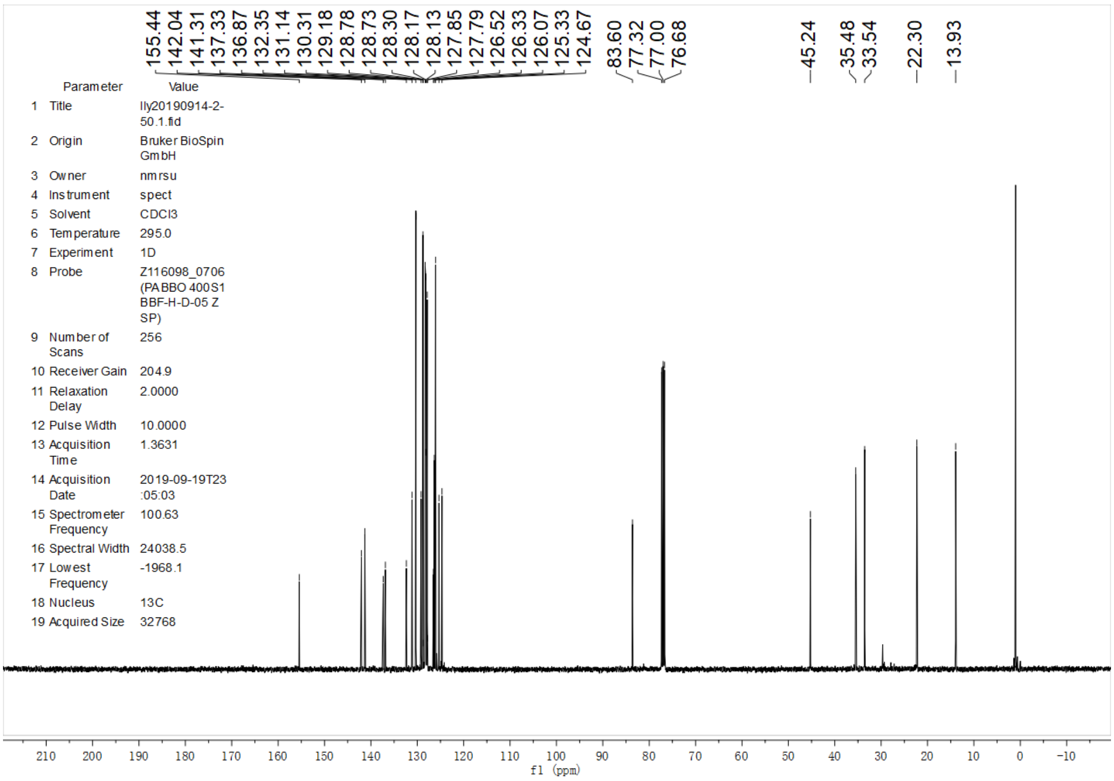

6-(tert-butyl)-2,4-diphenyl-4-((phenylthio)methyl)-4*H*-benzo[*d*][1,3]oxazine (3ia)

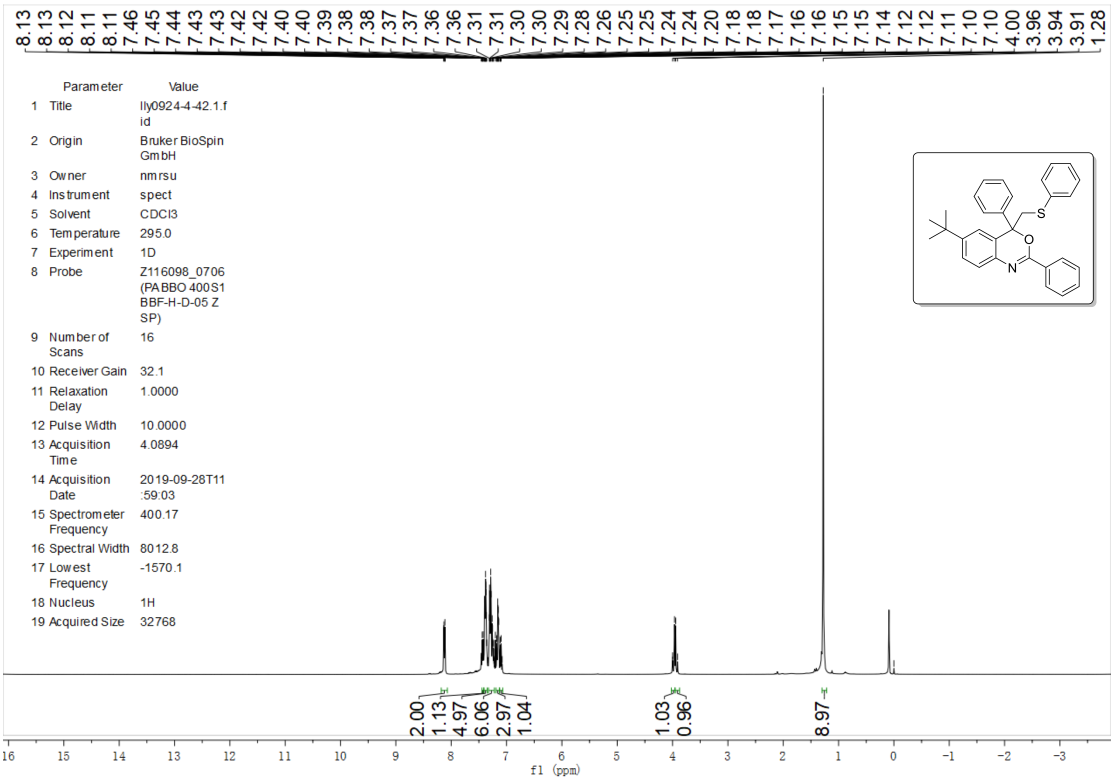

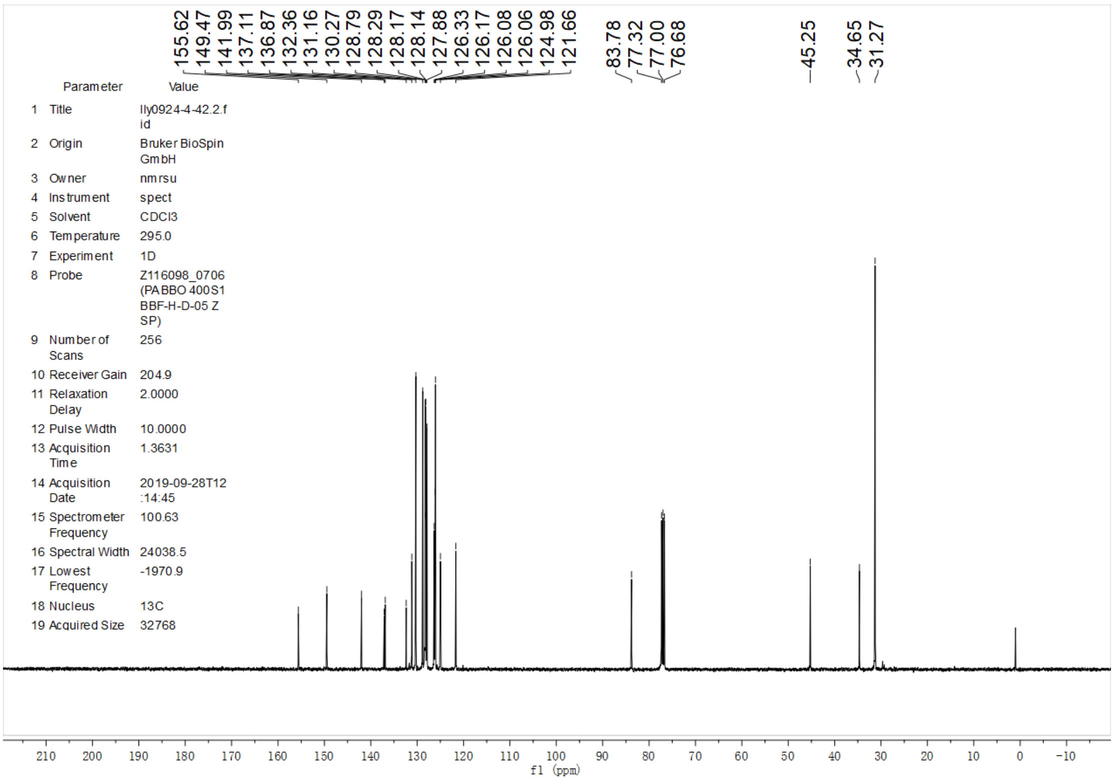

8-methyl-2,4-diphenyl-4-((phenylthio)methyl)-4*H*-benzo[*d*][1,3]oxazine (3ja)

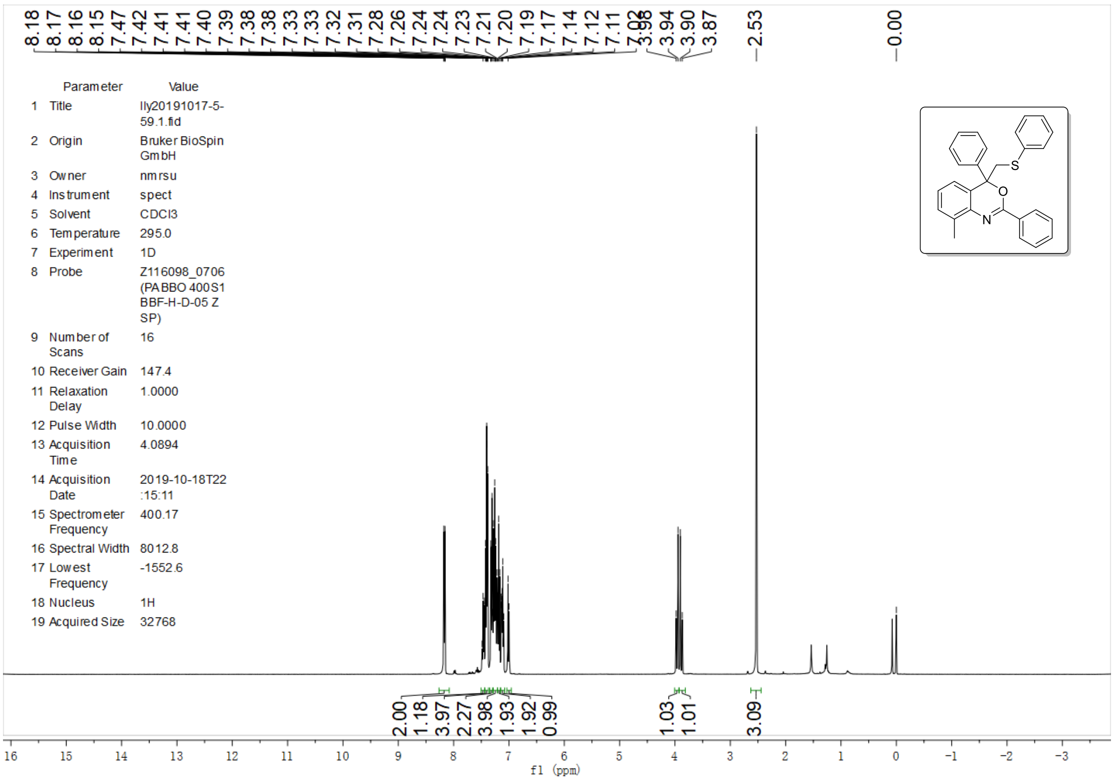

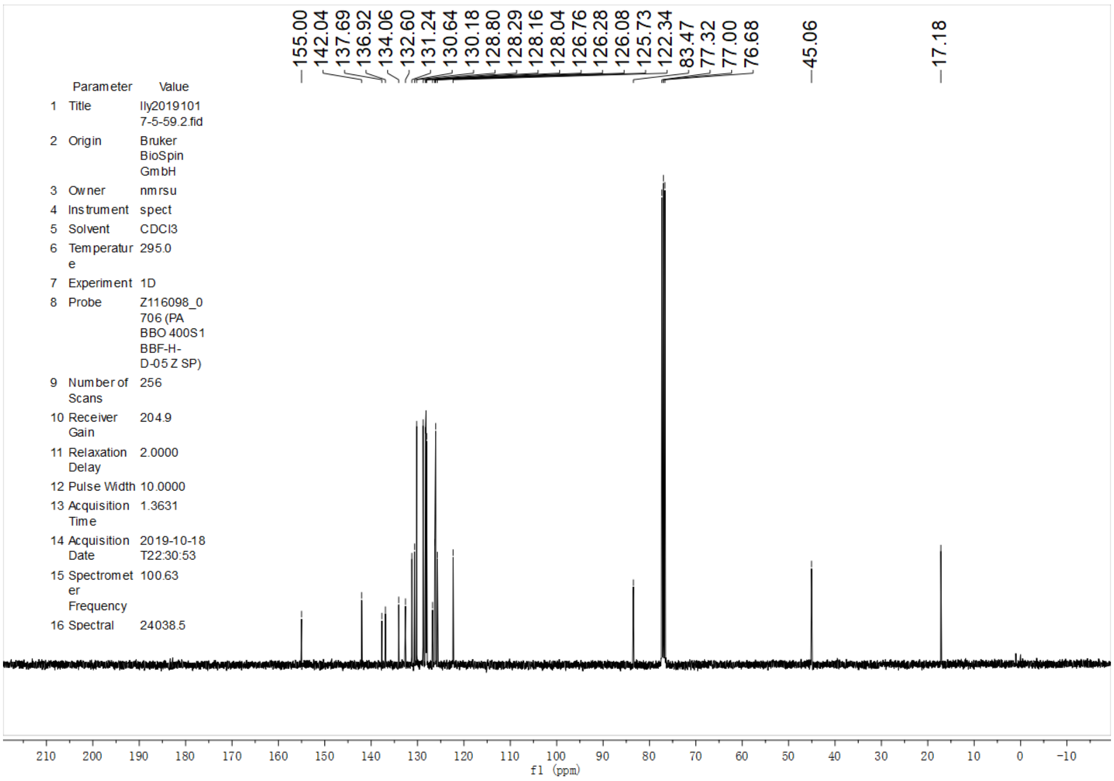

4-(4-fluorophenyl)-2-phenyl-4-((phenylthio)methyl)-4*H*-benzo[*d*][1,3]oxazine (3ka)

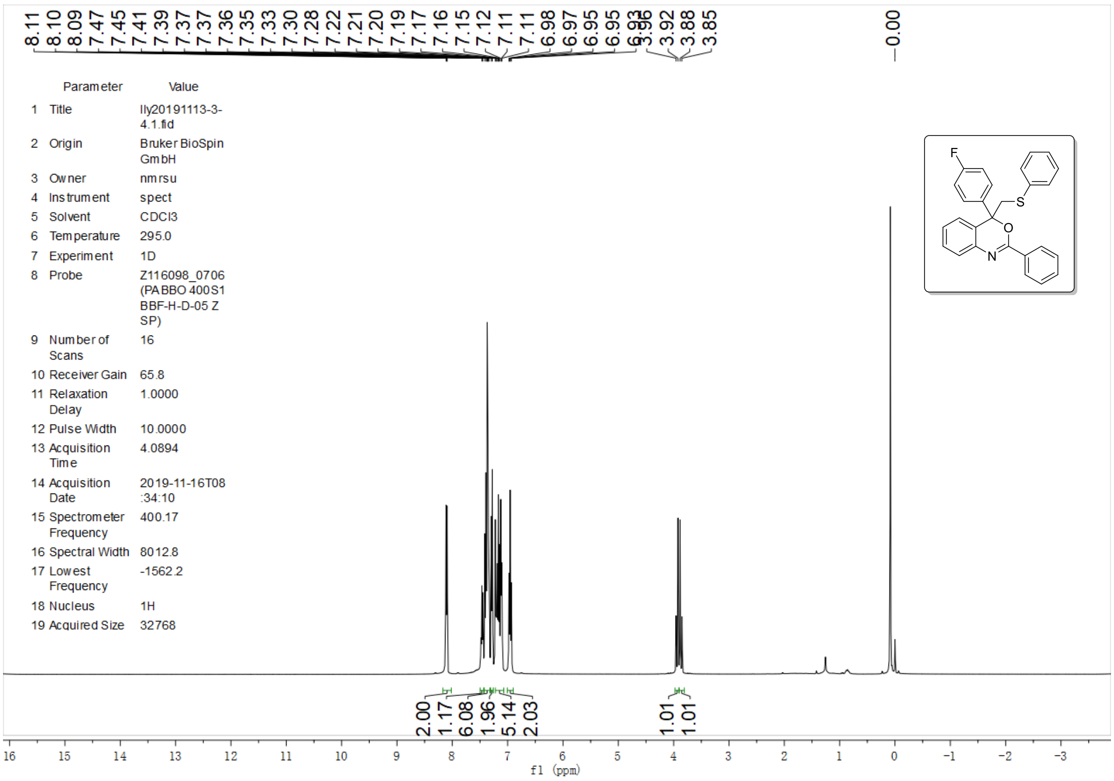

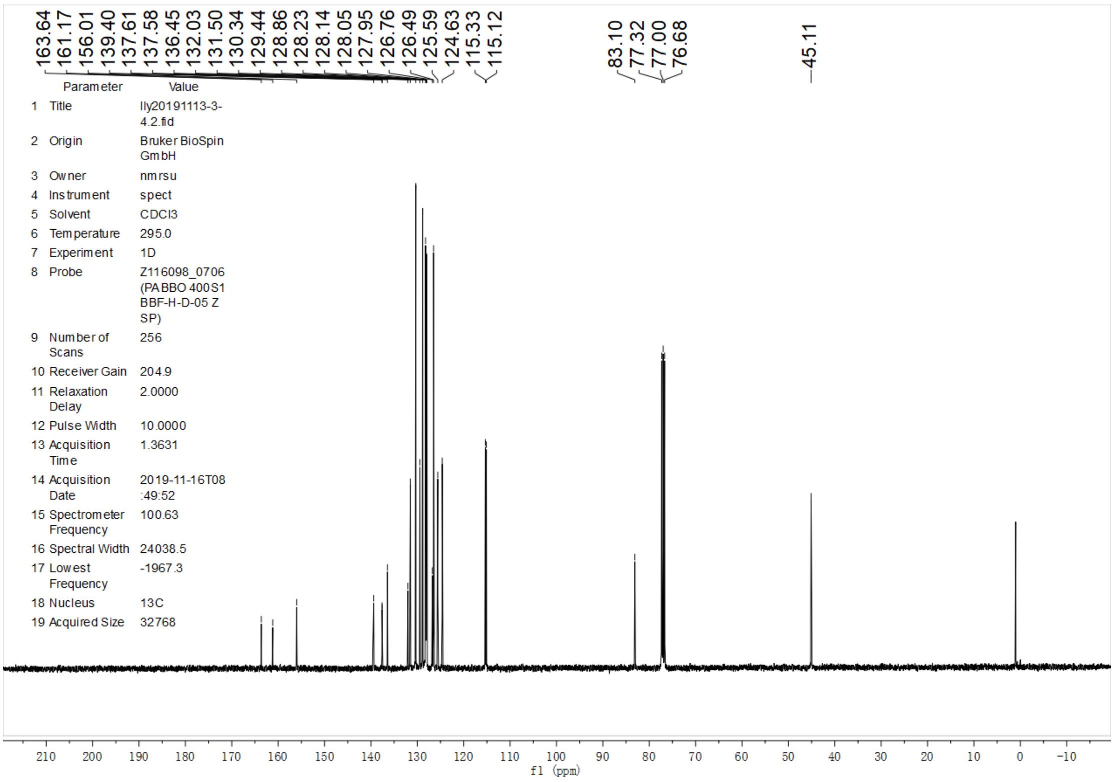

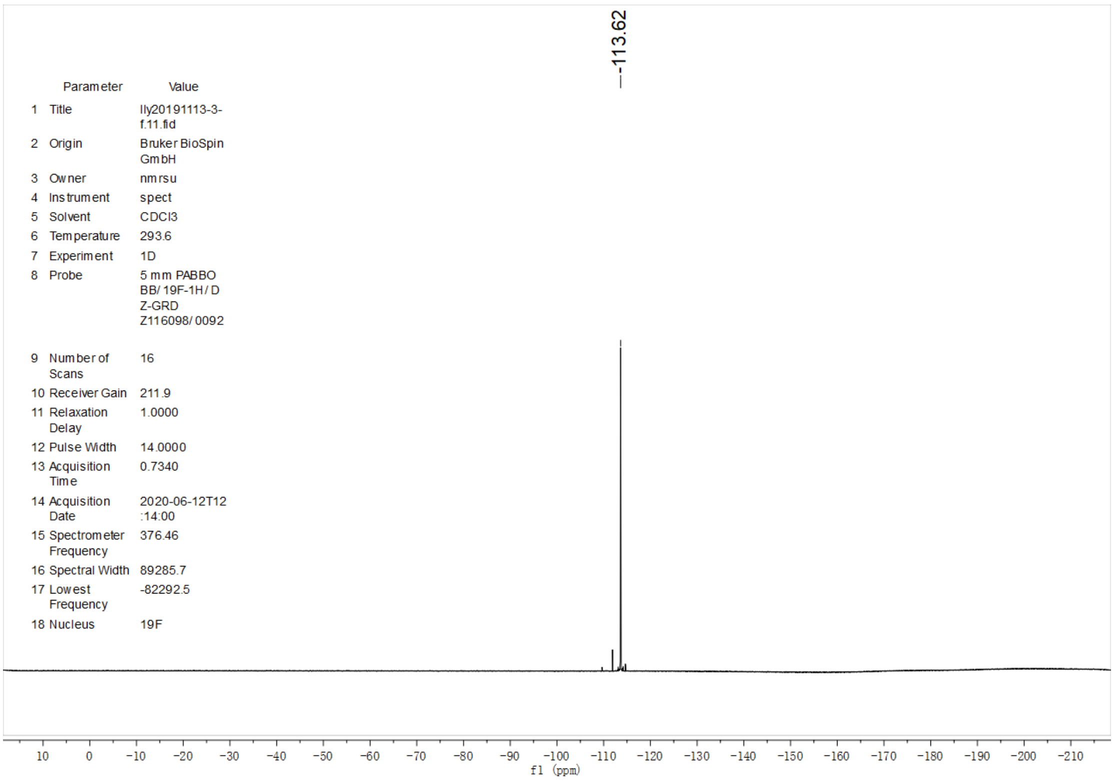

-113.62
Parameter Value
1 Title lly20191113-3-f.11.fid
2 Origin Bruker BioSpin GmbH
3 Owner nmrsu
4 Instrument spect
5 Solvent CDCl3
6 Temperature 293.6
7 Experiment 1D
8 Probe 5 mm PABBO BB/ 19F-1H/ D Z-GRD Z116098/ 0092
9 Number of Scans 16
10 Receiver Gain 211.9
11 Relaxation Delay 1.0000
12 Pulse Width 14.0000
13 Acquisition Time 0.7340
14 Acquisition Date 2020-06-12T12 :14:00
15 Spectrometer Frequency 376.46
16 Spectral Width 89285.7
17 Lowest Frequency -82292.5
18 Nucleus 19F
10 0 -10 -20 -30 -40 -50 -60 -70 -80 -90 -100 -110 -120 -130 -140 -150 -160 -170 -180 -190 -200 -210
f1 (ppm)

4-(4-chlorophenyl)-2-phenyl-4-((phenylthio)methyl)-4*H*-benzo[*d*][1,3]oxazine (3ma)

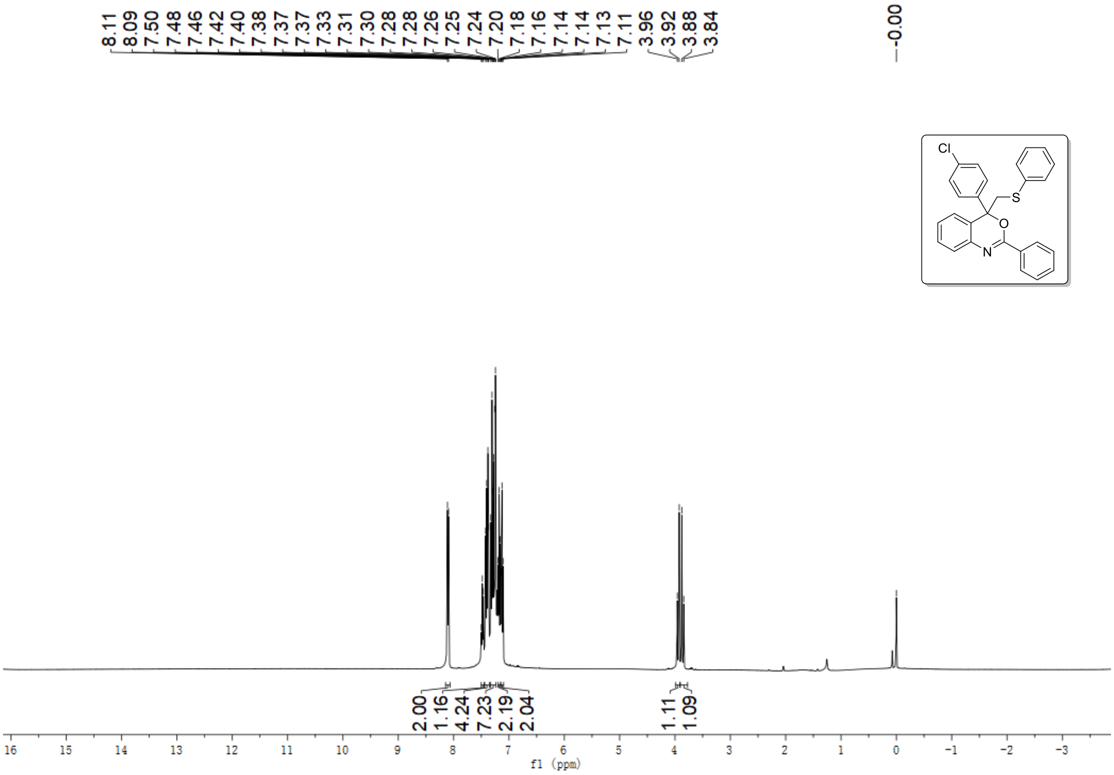

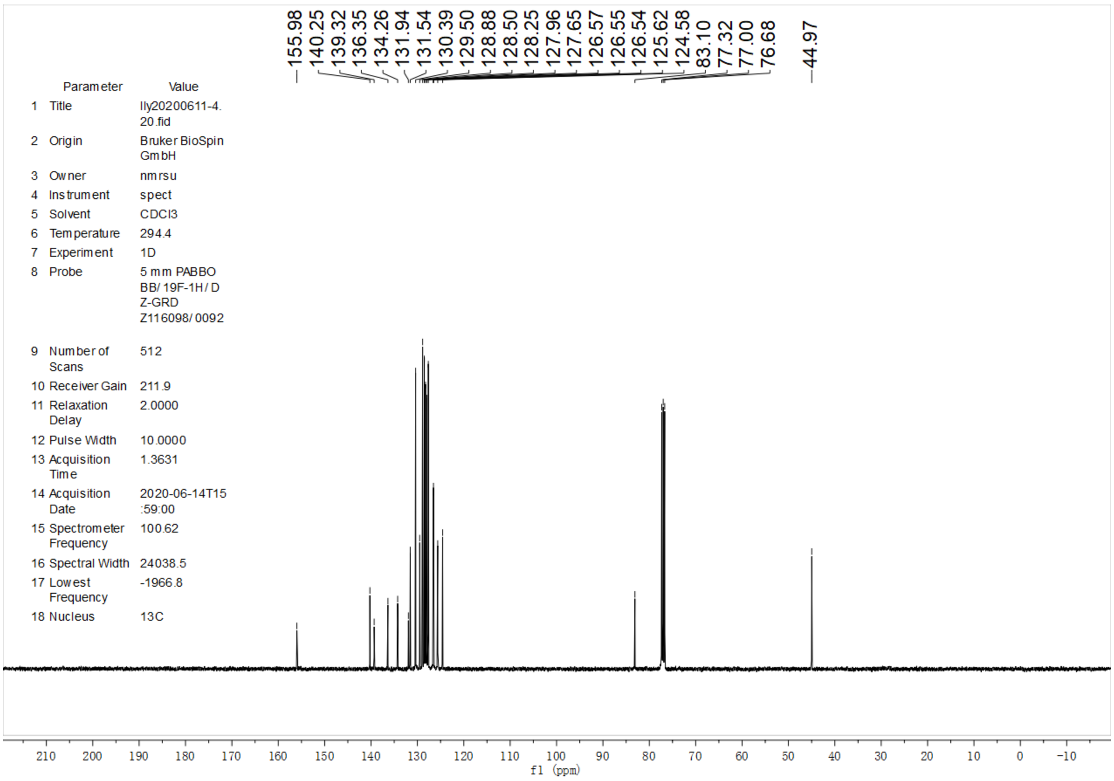

	Parameter	Value
1	Title	lly20200611-4.20.fid
2	Origin	Bruker BioSpin GmbH
3	Owner	nmrsu
4	Instrument	spect
5	Solvent	CDCl3
6	Temperature	294.4
7	Experiment	1D
8	Probe	5 mm PABBO BB/ 19F-1H/ D Z-GRD Z116098/ 0092
9	Number of Scans	512
10	Receiver Gain	211.9
11	Relaxation Delay	2.0000
12	Pulse Width	10.0000
13	Acquisition Time	1.3631
14	Acquisition Date	2020-06-14T15:59:00
15	Spectrometer Frequency	100.62
16	Spectral Width	24038.5
17	Lowest Frequency	-1966.8
18	Nucleus	13C

4-(4-bromophenyl)-2-phenyl-4-((phenylthio)methyl)-4H-benzo[d][1,3]oxazine (3na)

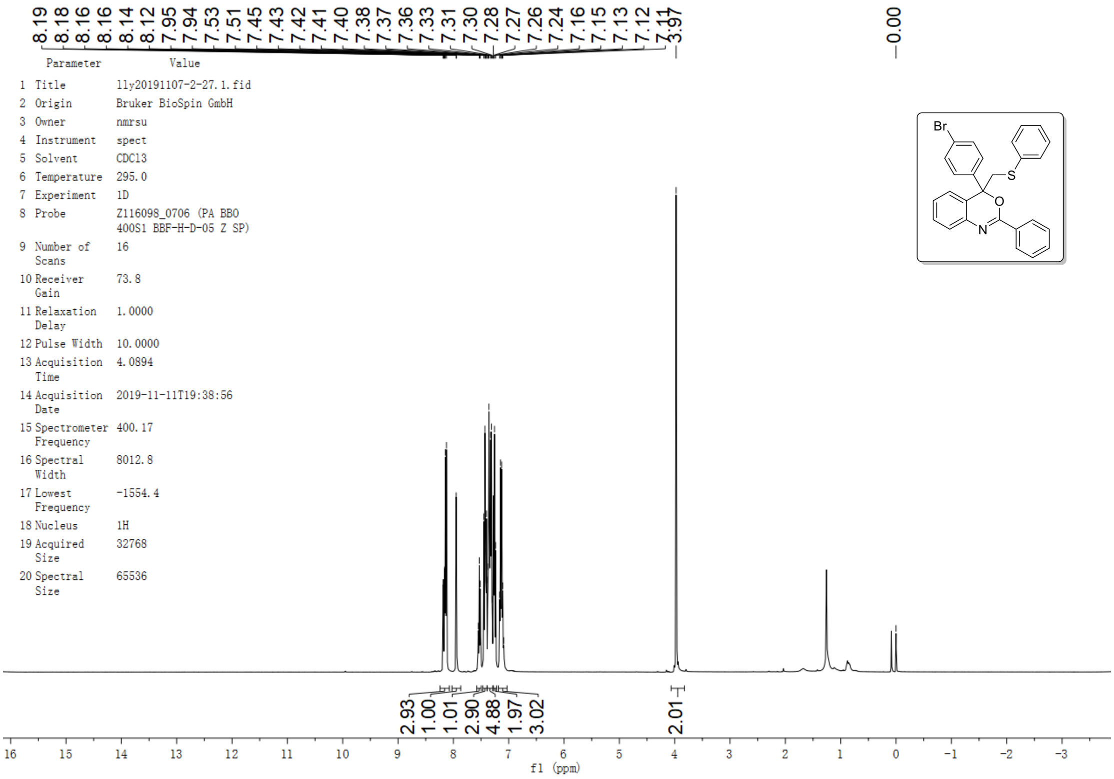

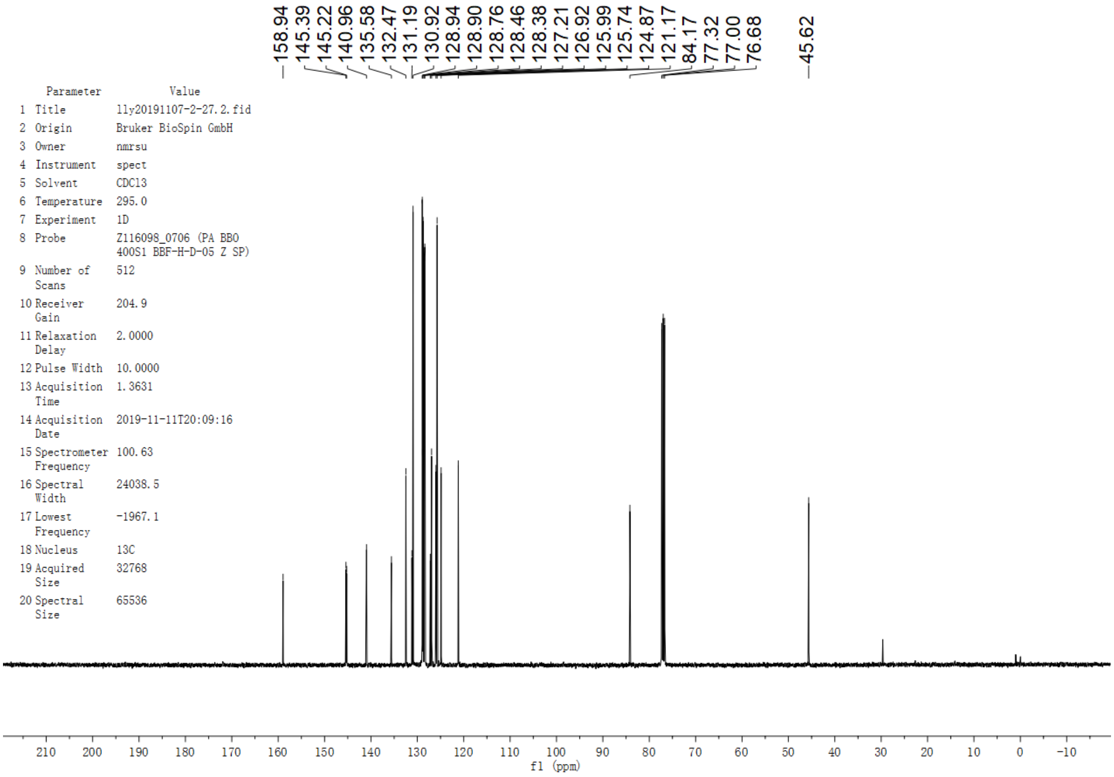

2-phenyl-4-((phenylthio)methyl)-4-(p-tolyl)-4*H*-benzo[*d*][1,3]oxazine (3oa)

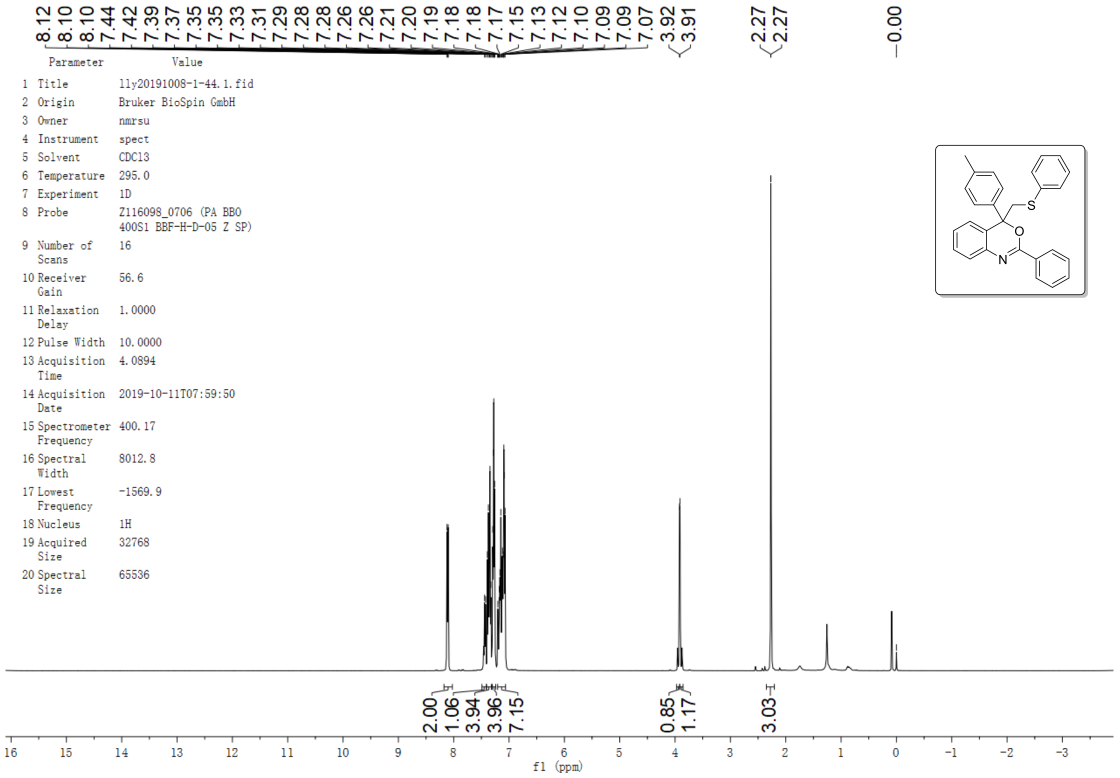

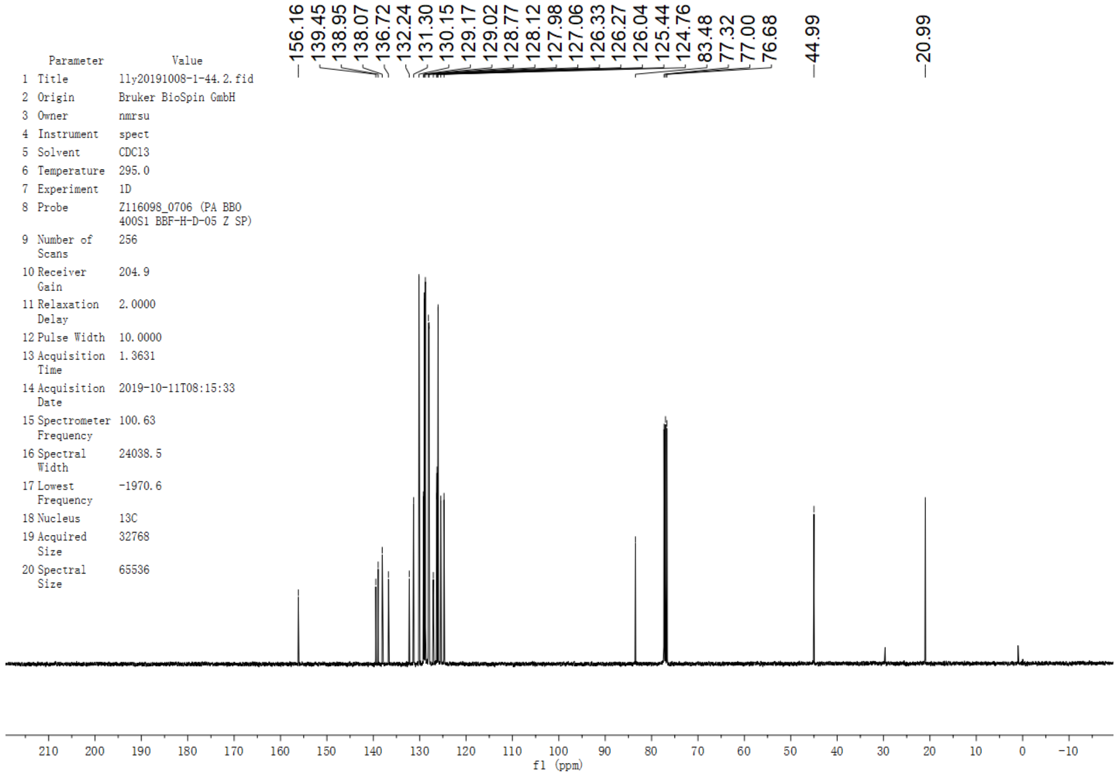

4-phenyl-4-((phenylthio)methyl)-2-(p-tolyl)-4*H*-benzo[*d*][1,3]oxazine (3pa)

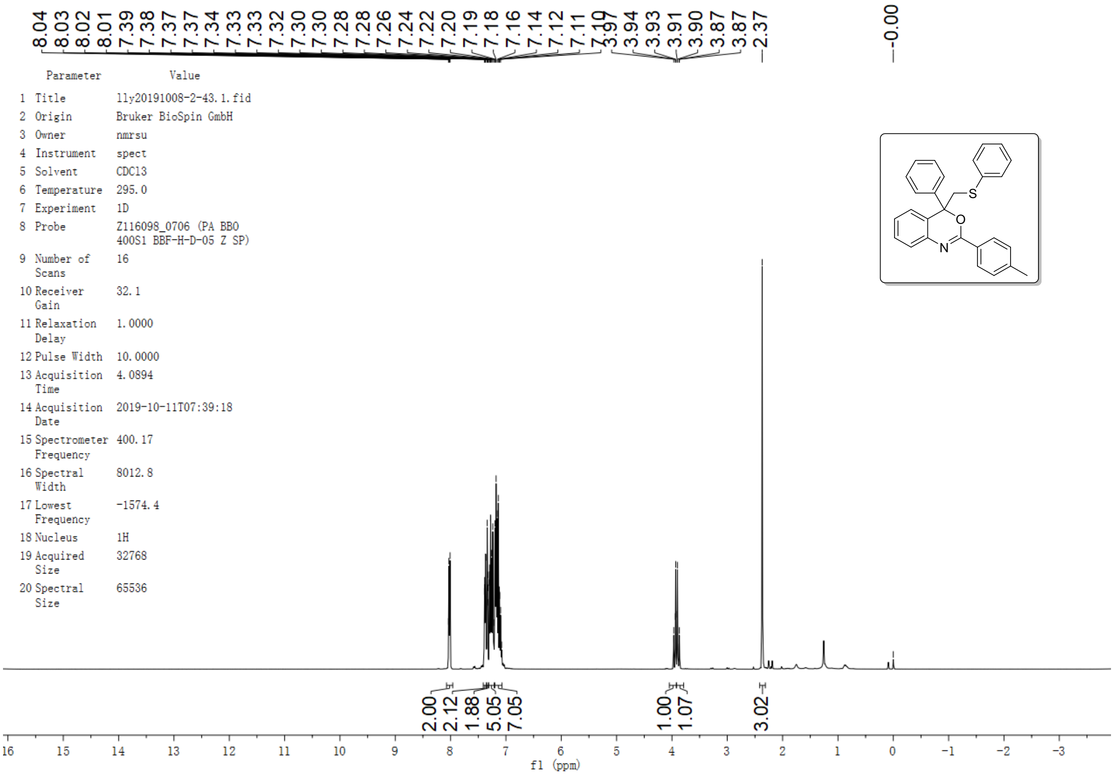

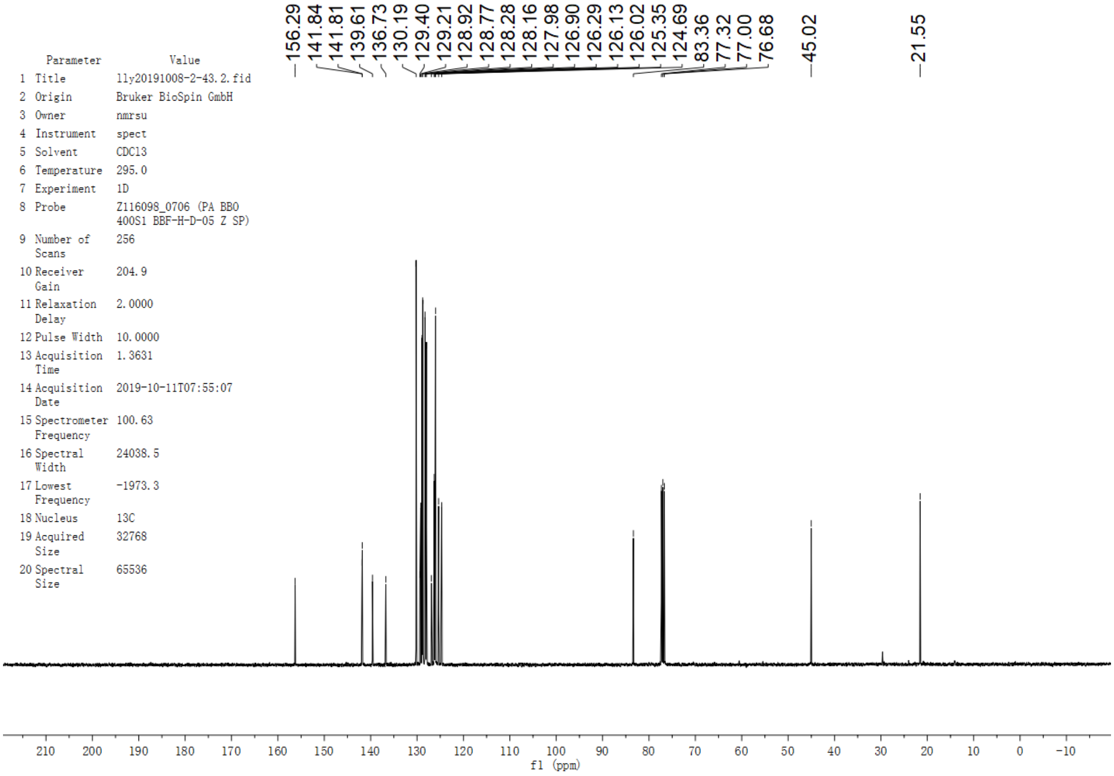

2-methyl-4-phenyl-4-((phenylthio)methyl)-4*H*-benzo[*d*][1,3]oxazine (3qa)

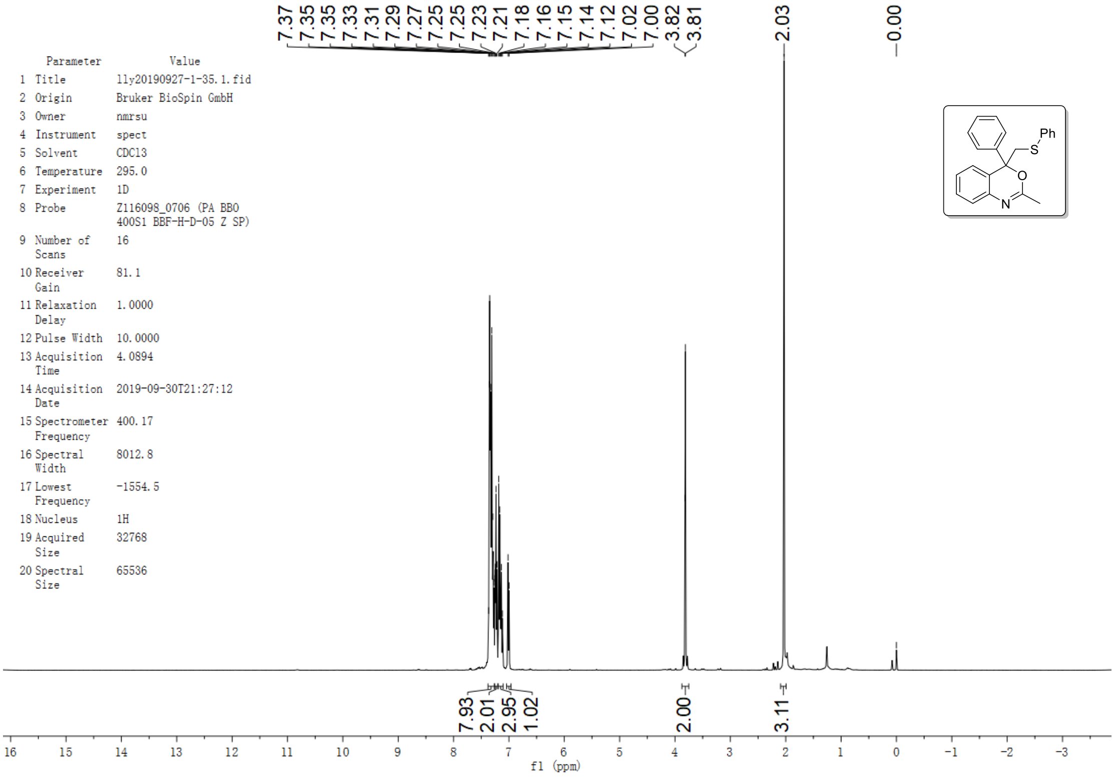

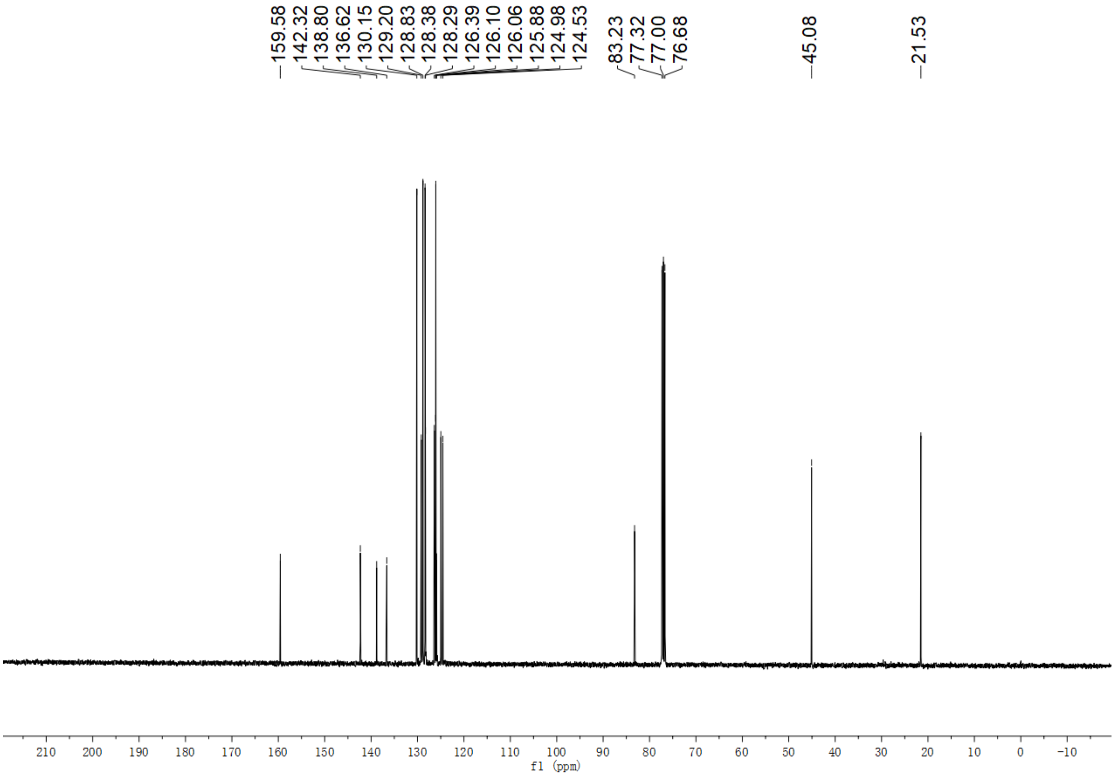

4-(((4-fluorophenyl)thio)methyl)-2,4-diphenyl-4*H*-benzo[*d*][1,3]oxazine (3ab)

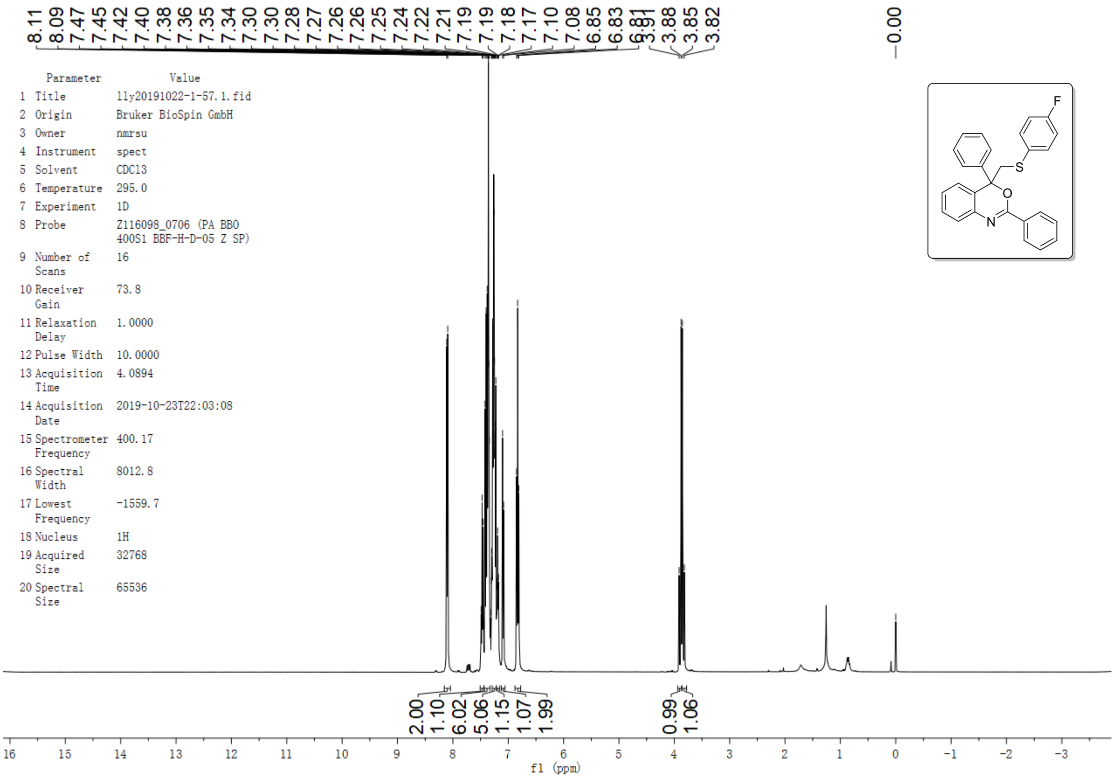

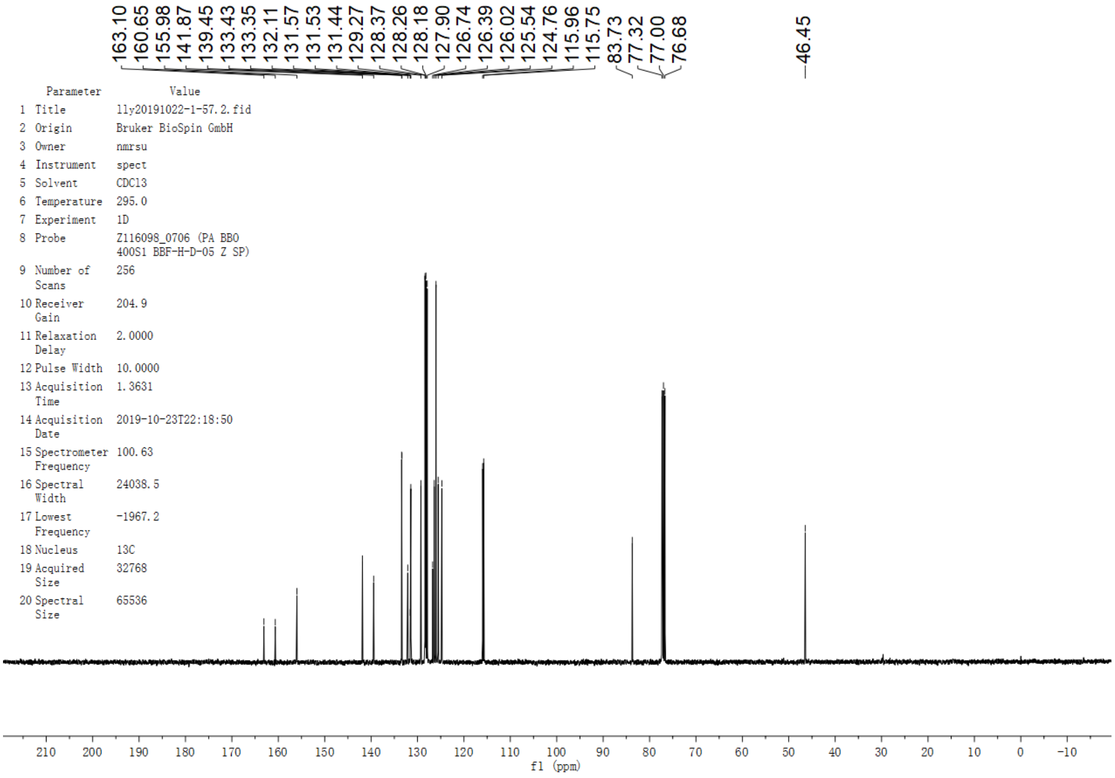

	Parameter	Value
1	Title	lly20191022-1-f.2.fid
2	Origin	Bruker BioSpin GmbH
3	Owner	nmrsu
4	Instrument	spect
5	Solvent	CDCl3
6	Temperature	297.1
7	Experiment	1D
8	Probe	5 mm PABBO BB/ 19F-1H/ D Z-GRD Z116098/ 0154
9	Number of Scans	16
10	Receiver Gain	207.2
11	Relaxation Delay	1.0000
12	Pulse Width	14.0000
13	Acquisition Time	0.7340
14	Acquisition Date	2020-06-17T10:10:00
15	Spectrometer Frequency	376.37
16	Spectral Width	89285.7
17	Lowest Frequency	-82283.1
18	Nucleus	19F
19	Acquired Size	65536
20	Spectral Size	131072

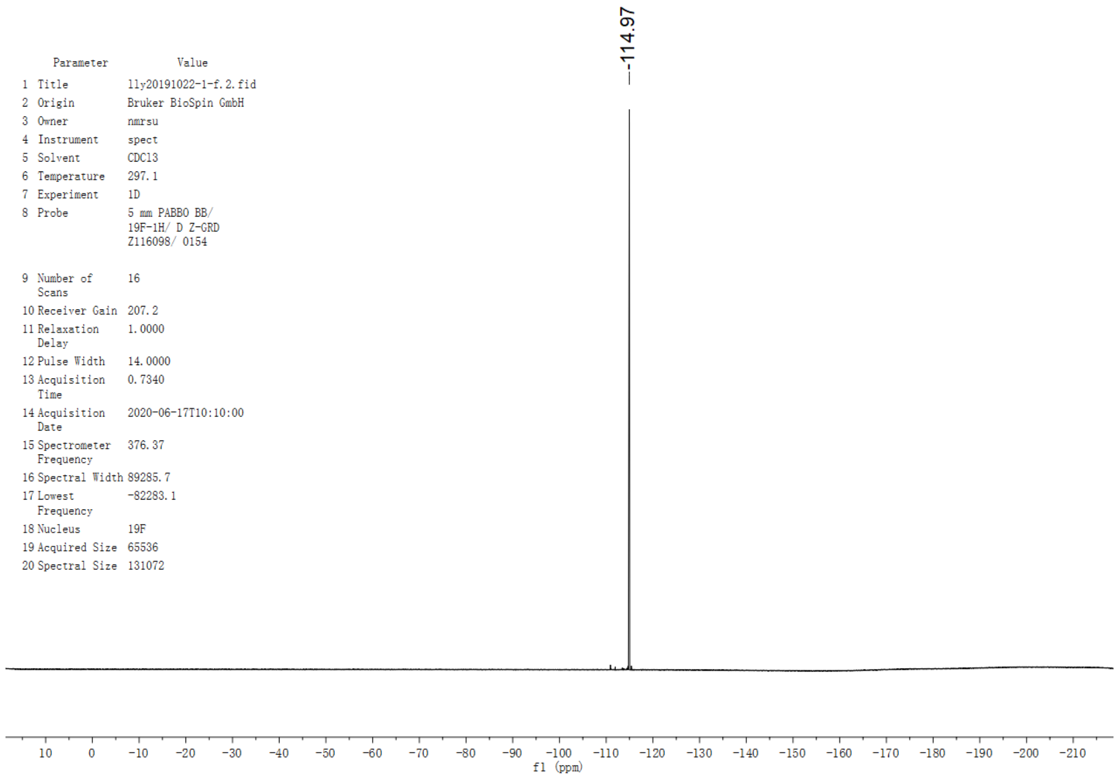

4-(((4-chlorophenyl)thio)methyl)-2,4-diphenyl-4*H*-benzo[*d*][1,3]oxazine (3ac)

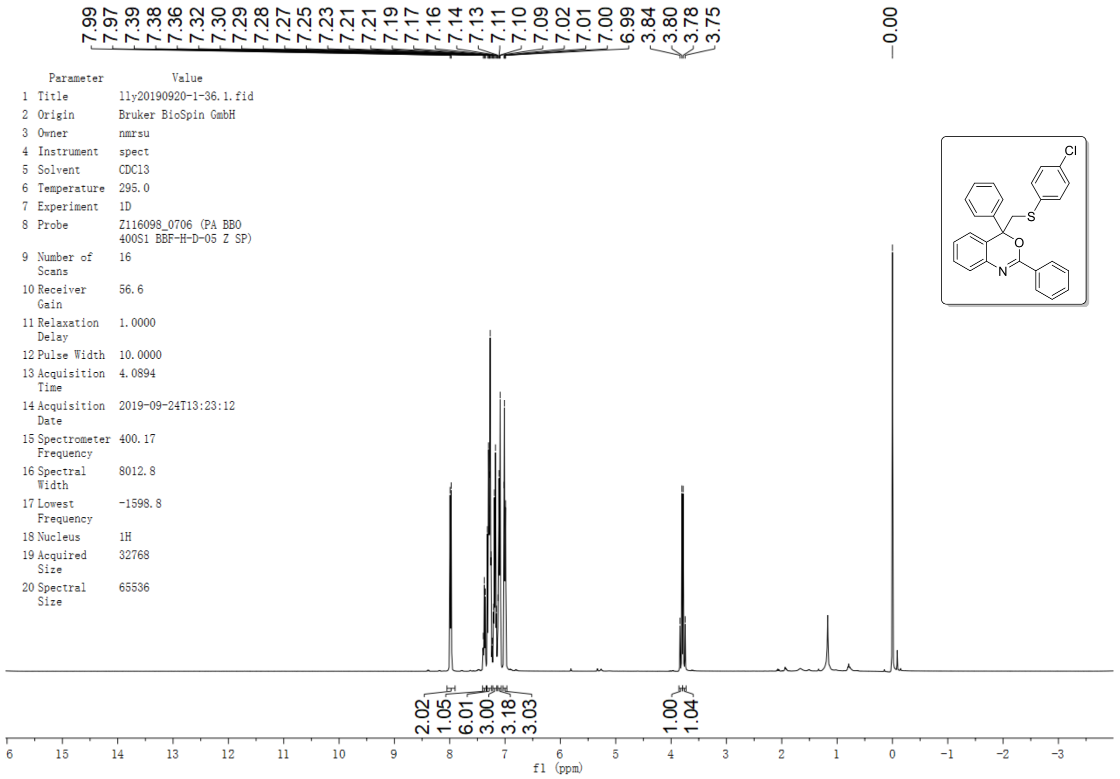

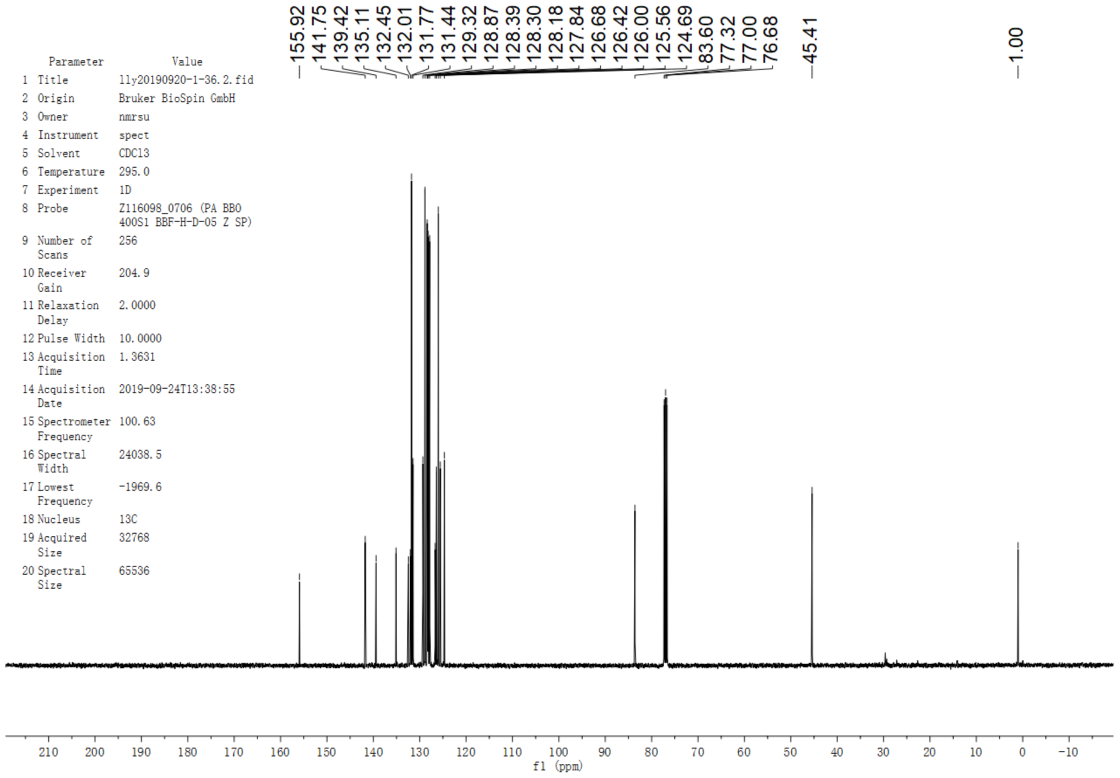

4-(((4-bromophenyl)thio)methyl)-2,4-diphenyl-4*H*-benzo[*d*][1,3]oxazine (3ad)

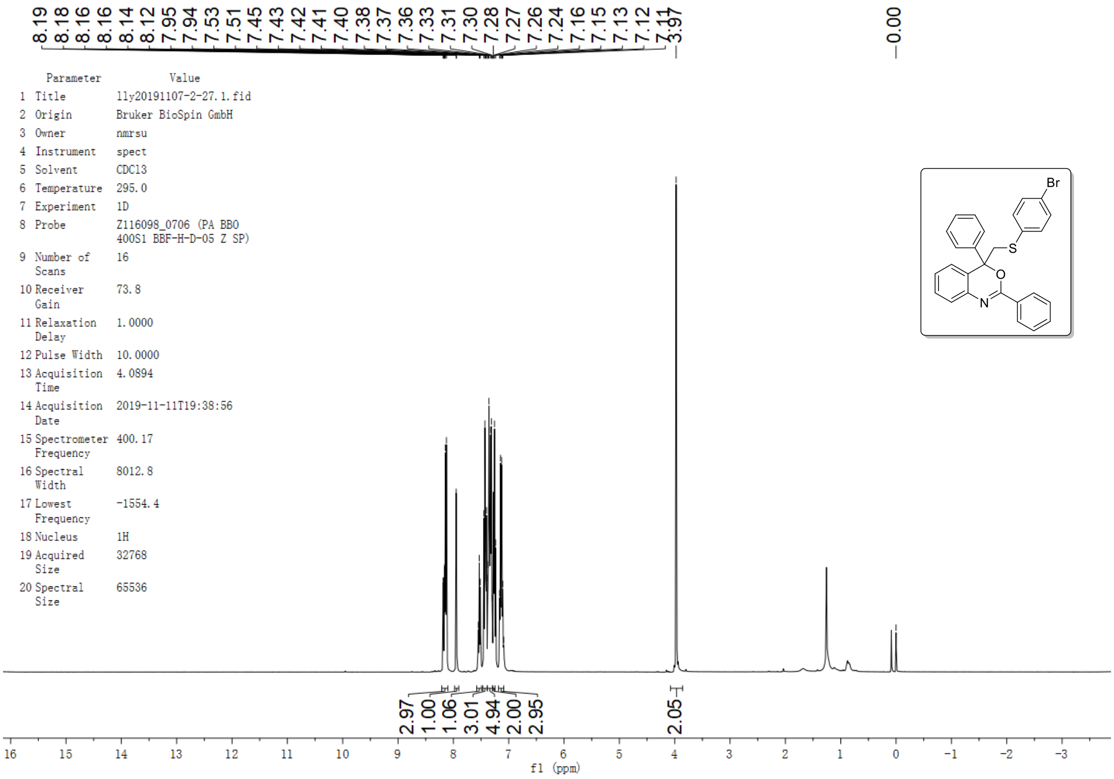

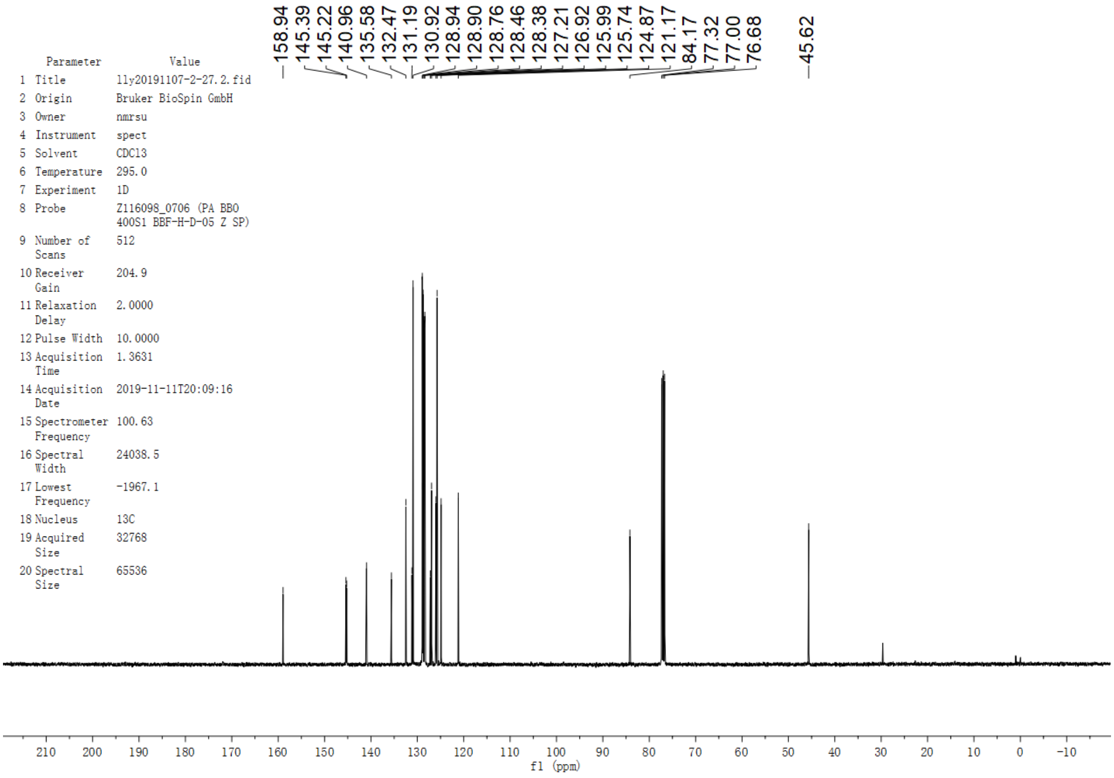

4-(((4-nitrophenyl)thio)methyl)-2,4-diphenyl-4*H*-benzo[*d*][1,3]oxazine (3ae)

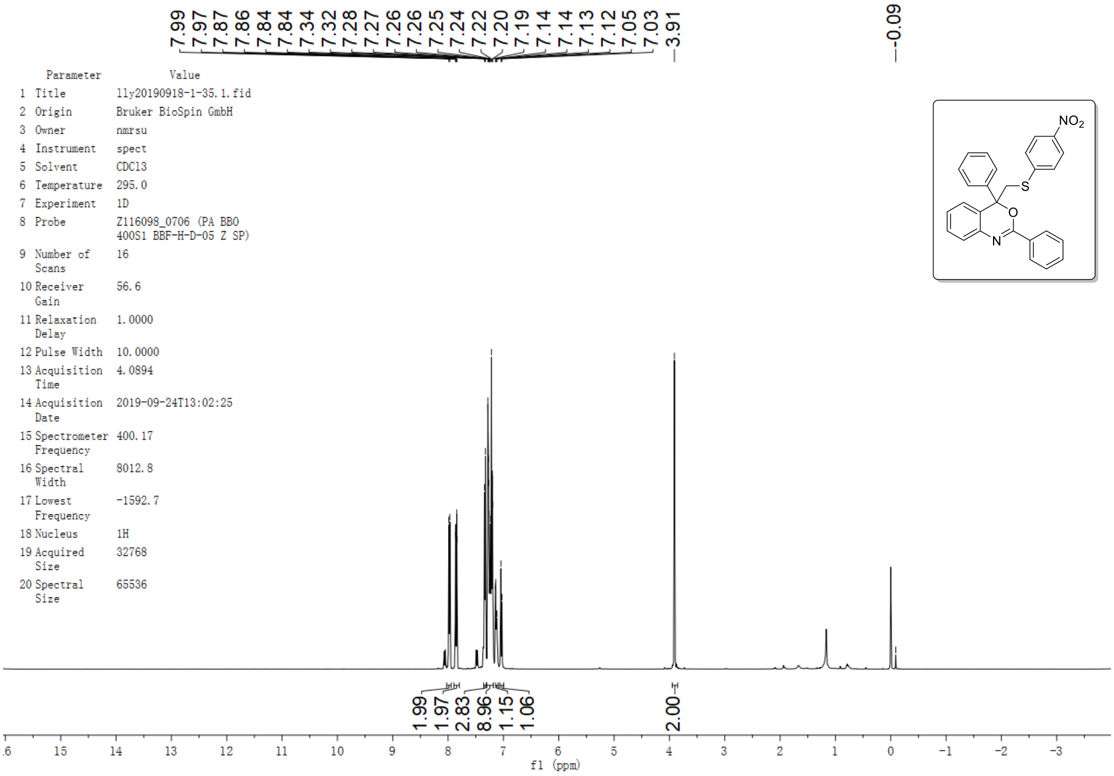

	Parameter	Value
1	Title	lly20190918-1-35.1.fid
2	Origin	Bruker BioSpin GmbH
3	Owner	nmrsu
4	Instrument	spect
5	Solvent	CDCl3
6	Temperature	295.0
7	Experiment	1D
8	Probe	Z116098_0706 (PA BBO 400S1 BBF-H-D-05 Z SP)
9	Number of Scans	16
10	Receiver Gain	56.6
11	Relaxation Delay	1.0000
12	Pulse Width	10.0000
13	Acquisition Time	4.0894
14	Acquisition Date	2019-09-24T13:02:25
15	Spectrometer Frequency	400.17
16	Spectral Width	8012.8
17	Lowest Frequency	-1592.7
18	Nucleus	1H
19	Acquired Size	32768
20	Spectral Size	65536

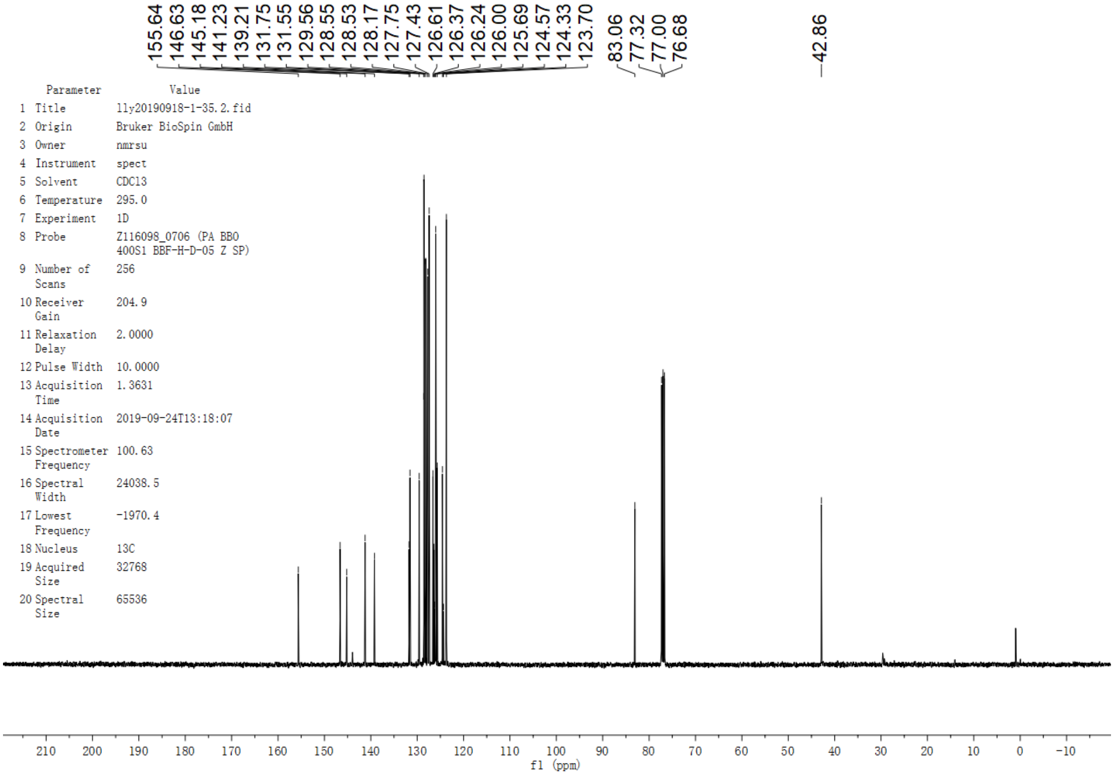

	Parameter	Value
1	Title	lly20190918-1-35.2.fid
2	Origin	Bruker BioSpin GmbH
3	Owner	nmrsu
4	Instrument	spect
5	Solvent	CDCl3
6	Temperature	295.0
7	Experiment	1D
8	Probe	Z116098_0706 (PA BBO 400S1 BBF-H-D-05 Z SP)
9	Number of Scans	256
10	Receiver Gain	204.9
11	Relaxation Delay	2.0000
12	Pulse Width	10.0000
13	Acquisition Time	1.3631
14	Acquisition Date	2019-09-24T13:18:07
15	Spectrometer Frequency	100.63
16	Spectral Width	24038.5
17	Lowest Frequency	-1970.4
18	Nucleus	13C
19	Acquired Size	32768
20	Spectral Size	65536

4-(((4-methoxyphenyl)thio)methyl)-2,4-diphenyl-4*H*-benzo[*d*][1,3]oxazine (3af)

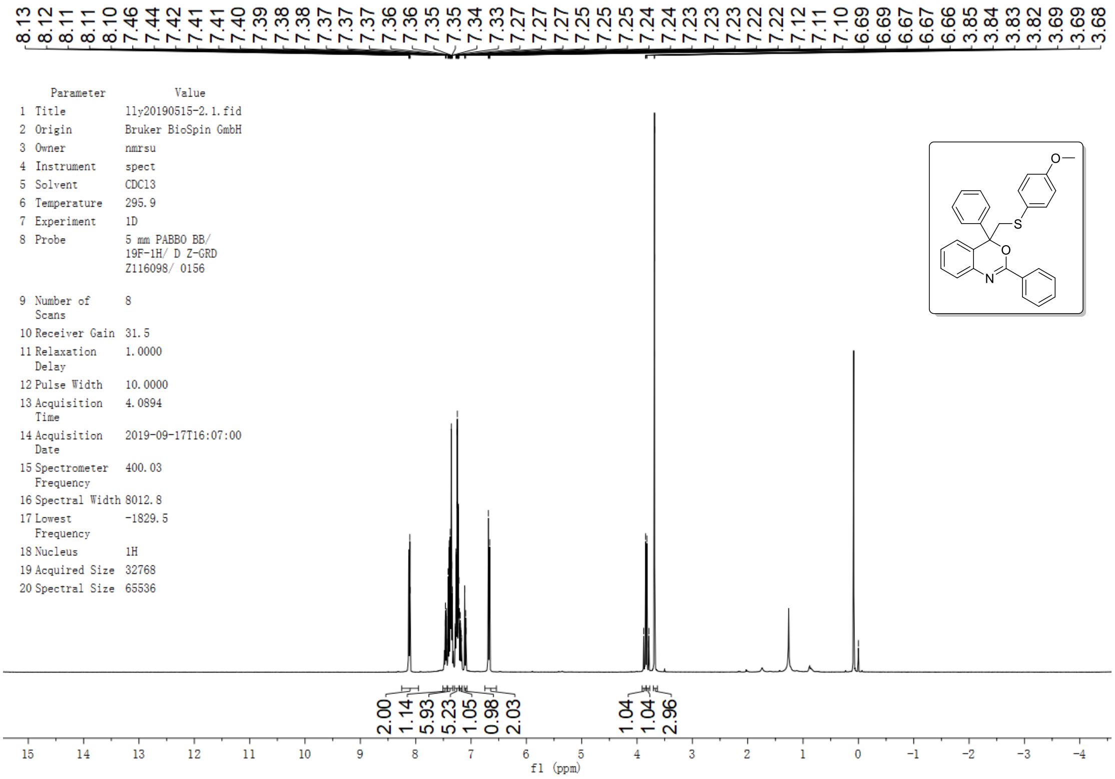

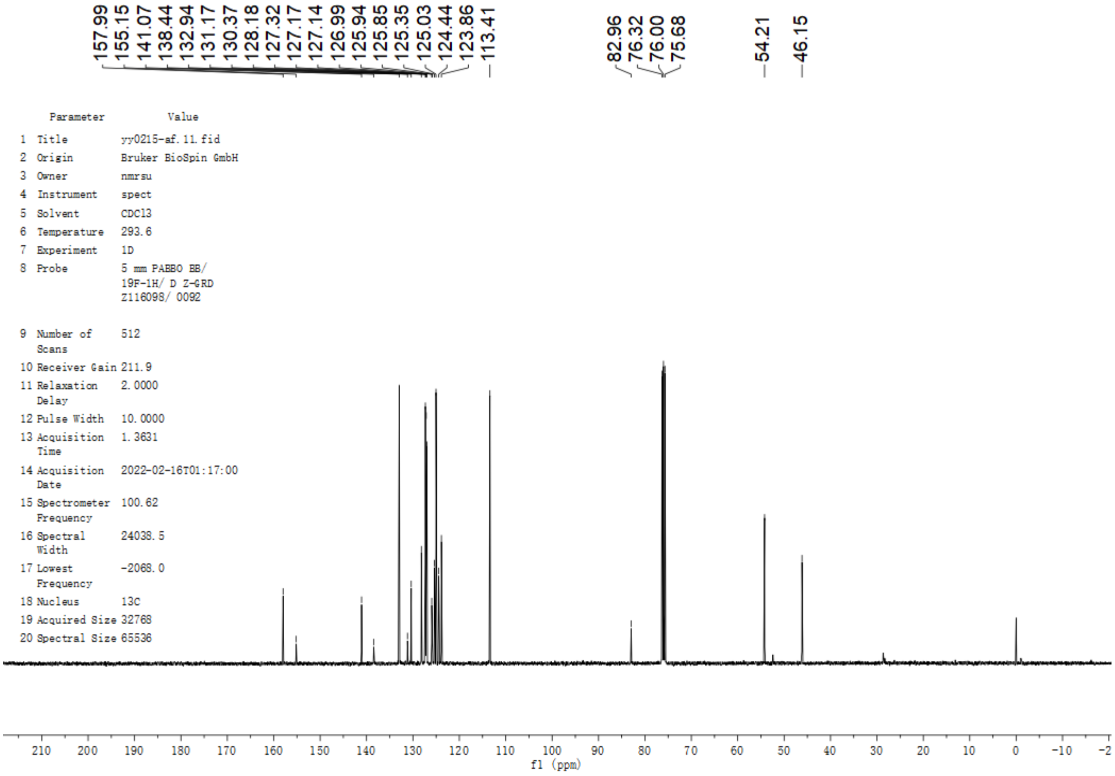

2,4-diphenyl-4-((p-tolylthio)methyl)-4*H*-benzo[*d*][1,3]oxazine (3ag)

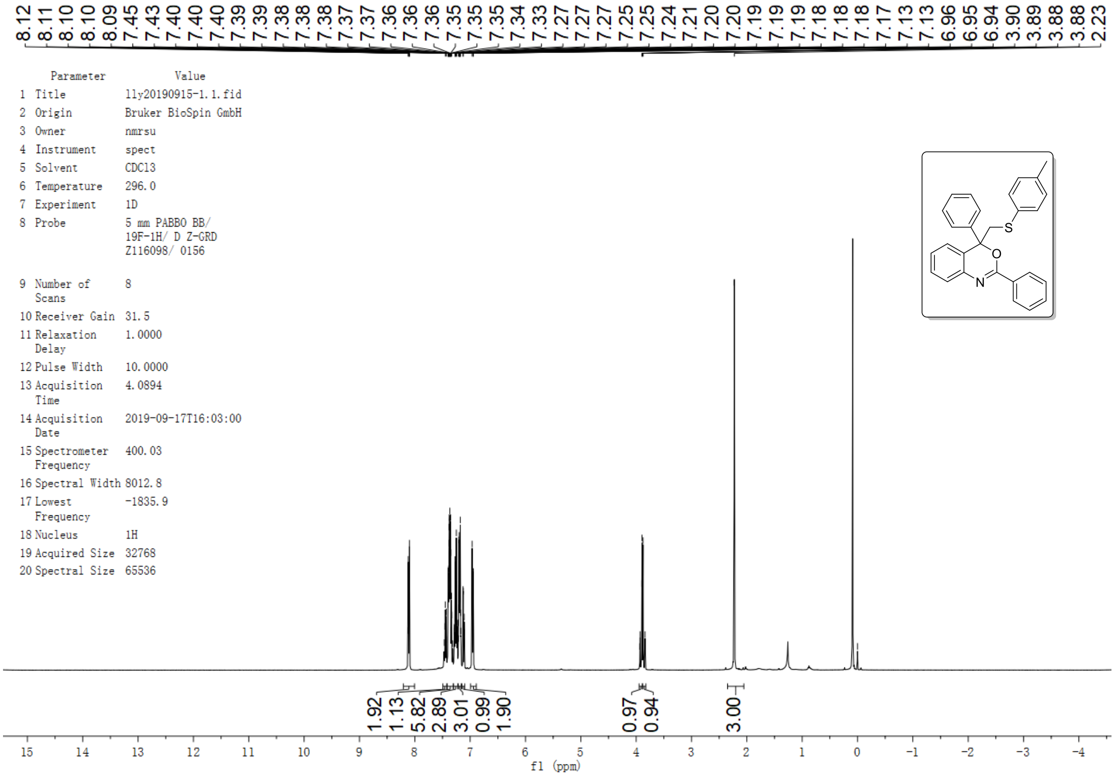

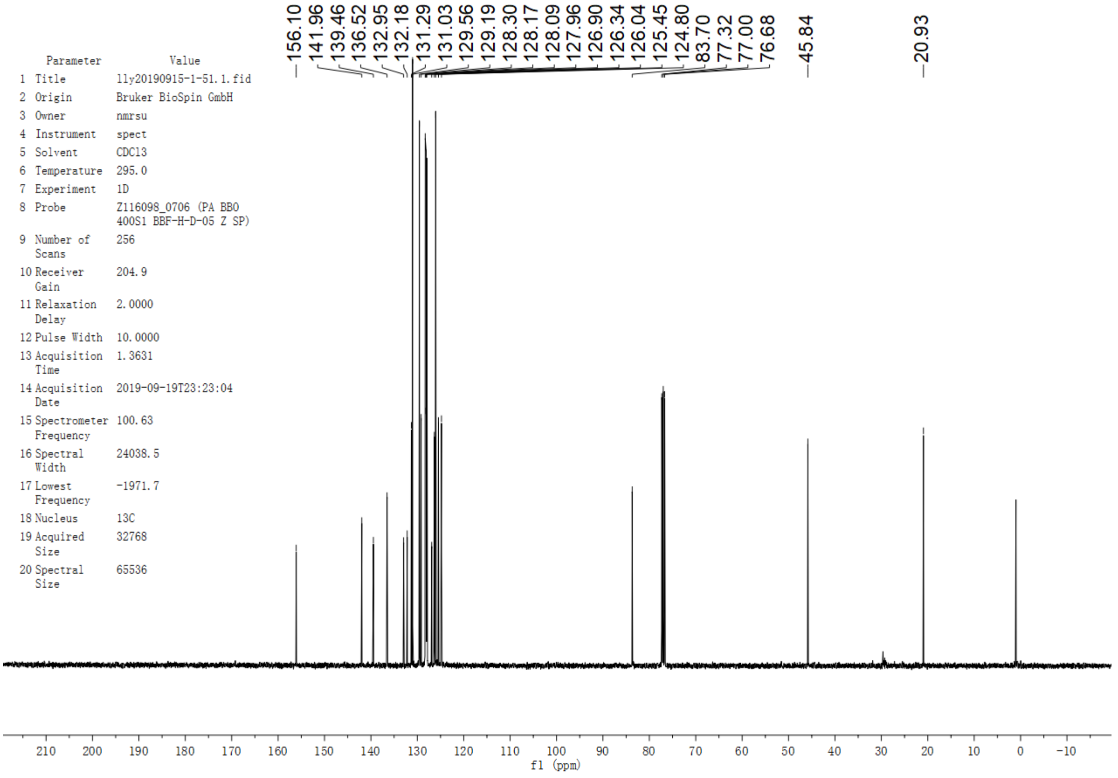

2,4-diphenyl-4-((m-tolylthio)methyl)-4*H*-benzo[*d*][1,3]oxazine (3ah)

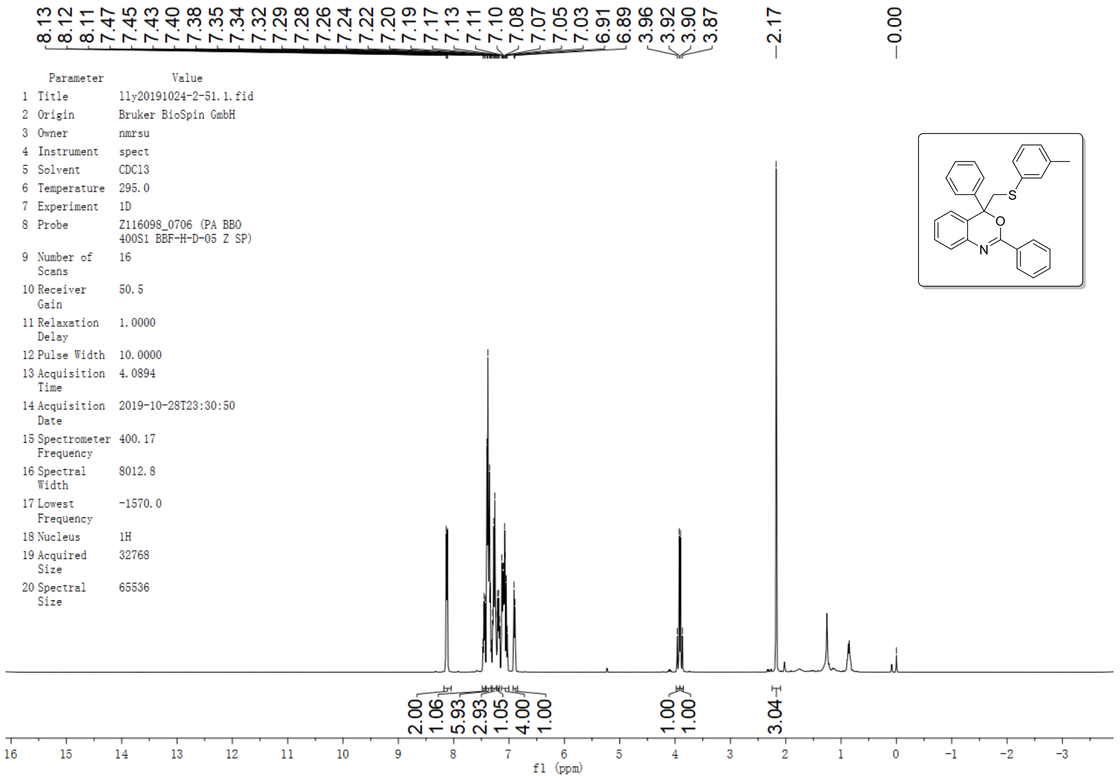

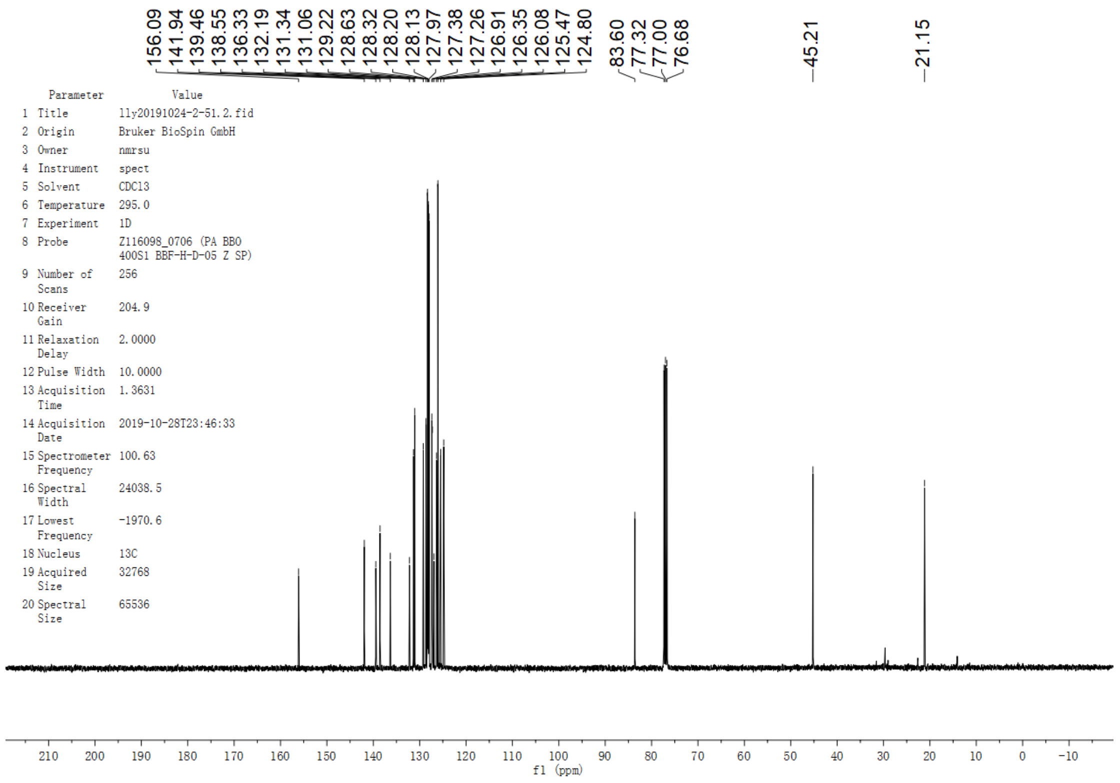

.

2,4-diphenyl-4-((o-tolylthio)methyl)-4*H*-benzo[*d*][1,3]oxazine (3ai)

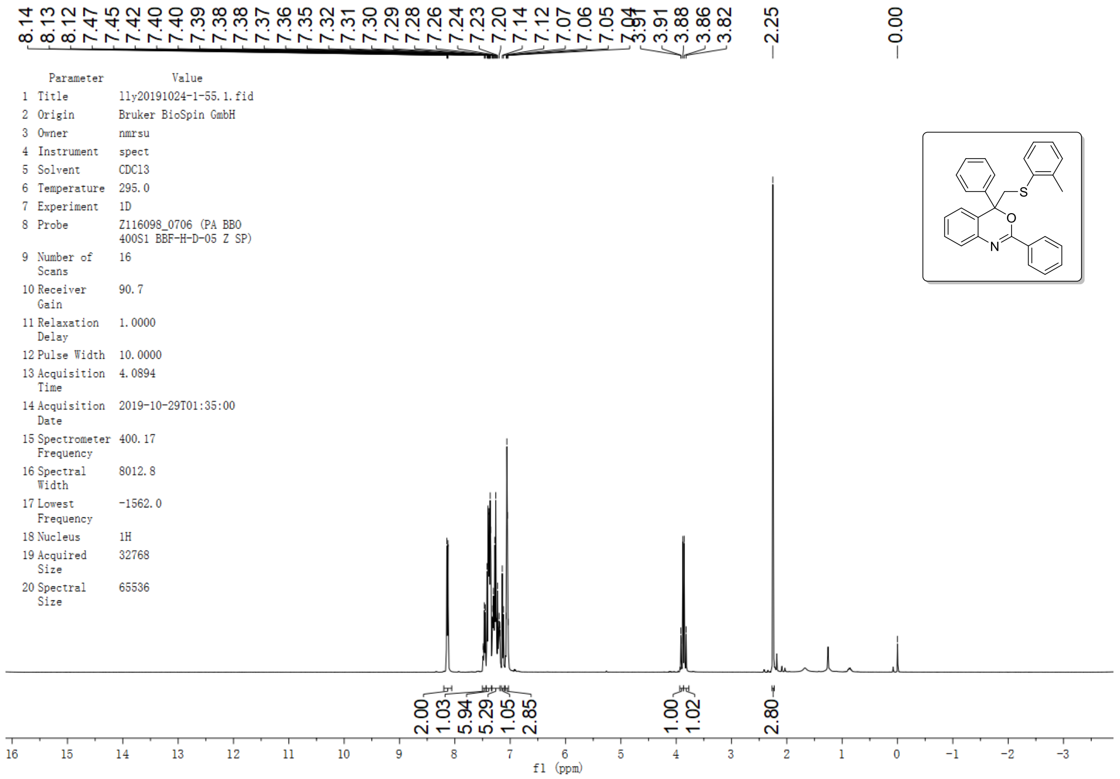

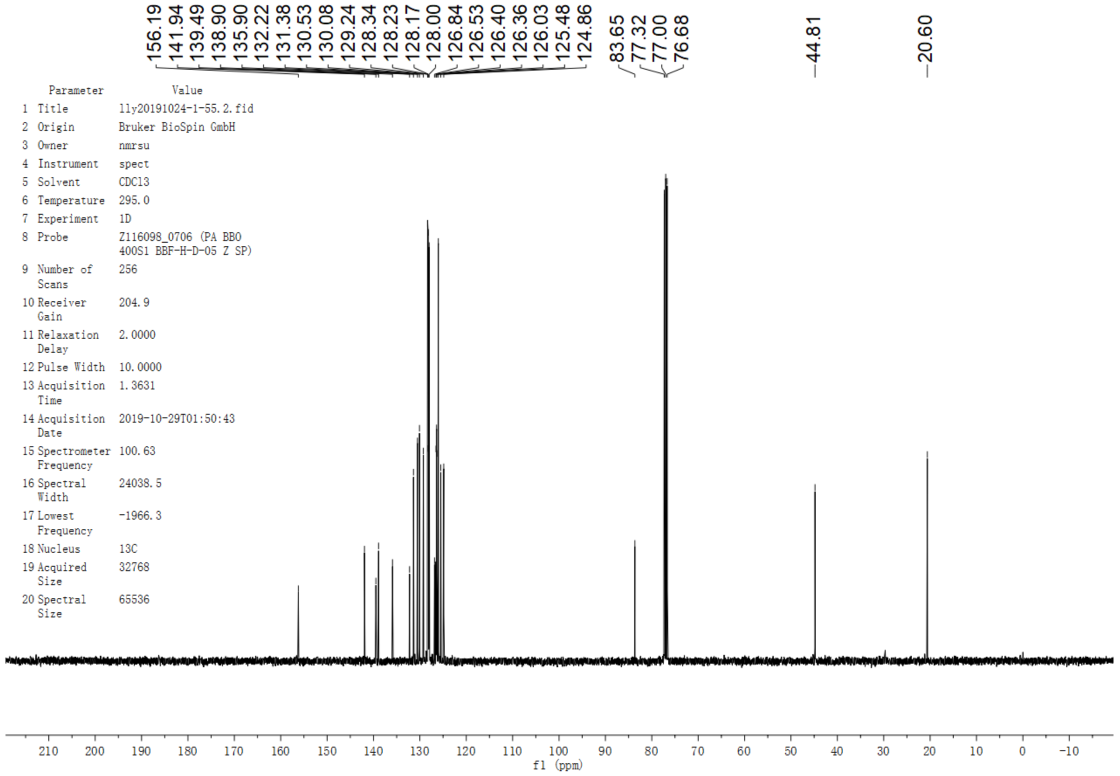

2,4-diphenyl-4-((propylthio)methyl)-4*H*-benzo[*d*][1,3]oxazine (3ak)

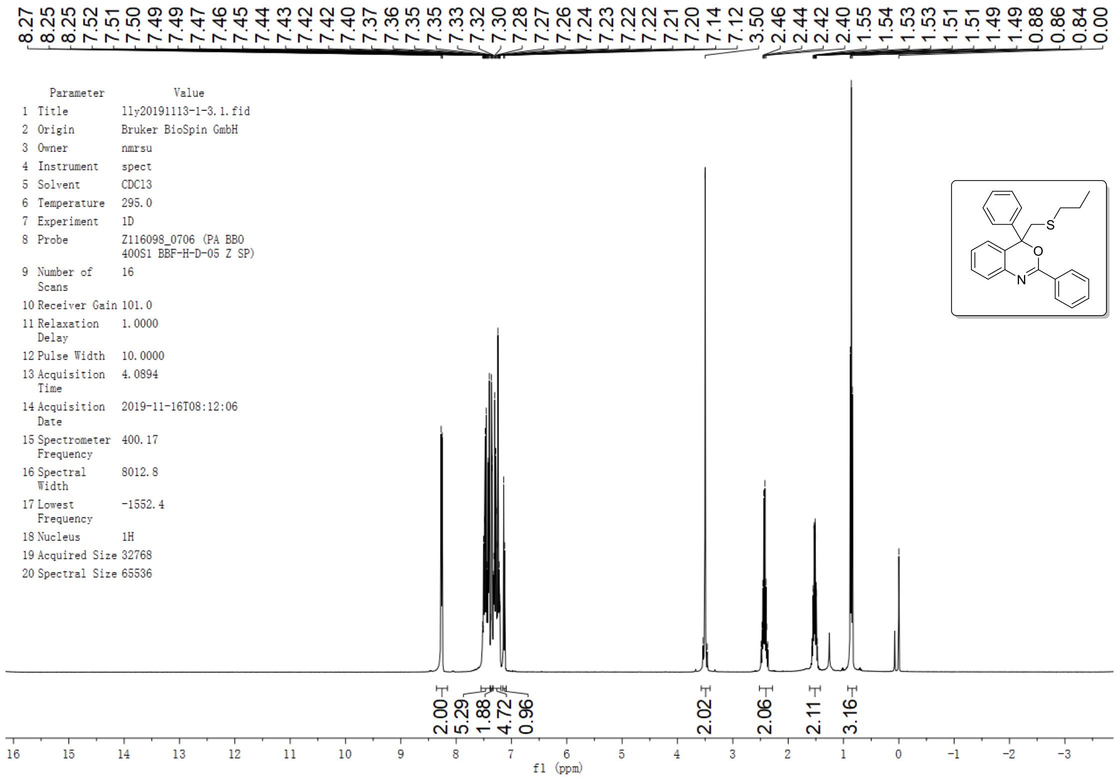

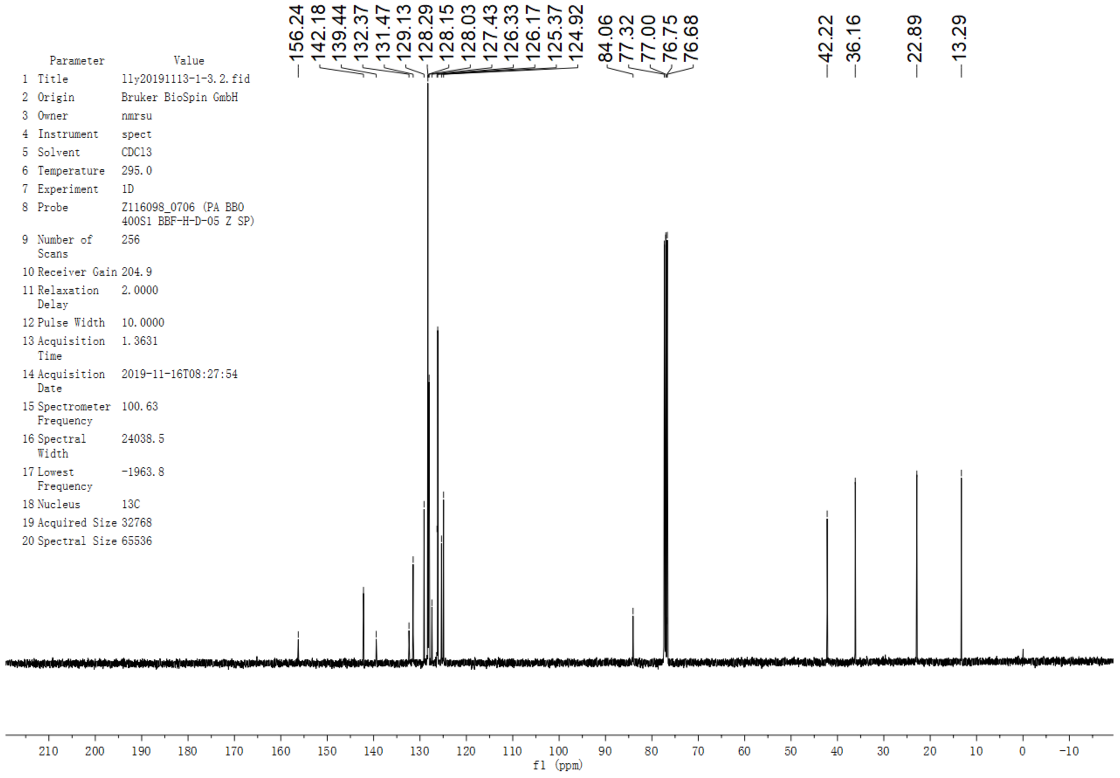

4-((cyclohexylthio)methyl)-2,4-diphenyl-4*H*-benzo[*d*][1,3]oxazine (3al)

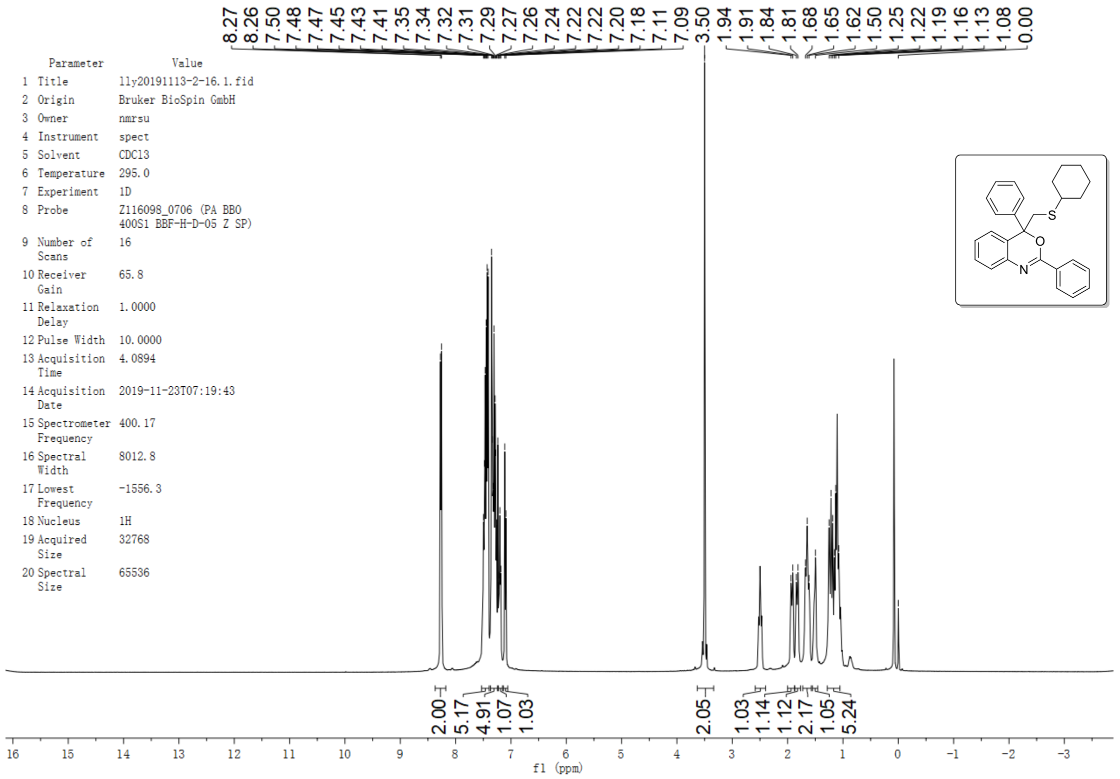

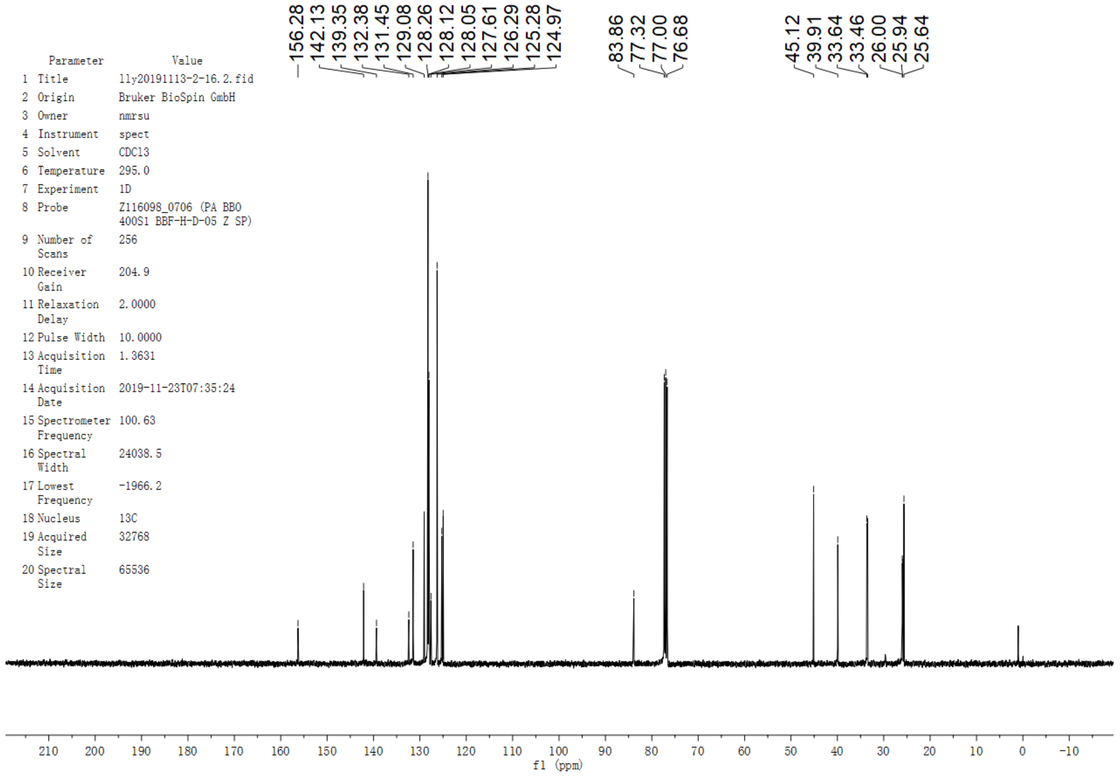

Supplement: RA-012-D2RA01078J-s001 [file RA-012-D2RA01078J-s001.pdf]
